# Identifying Candidate Reference Chemicals for *In Vitro* Testing of the Retinoid Pathway for Predictive Developmental Toxicity

**DOI:** 10.14573/altex.2202231

**Published:** 2022-06-23

**Authors:** Nancy C. Baker, Jocylin D. Pierro, Laura W. Taylor, Thomas B. Knudsen

**Affiliations:** 1 Leidos, Research Triangle Park, NC, USA; 2 Center for Computational Toxicology and Exposure, U.S. Environmental Protection Agency, Research Triangle Park, NC, USA

## Abstract

Evaluating chemicals for potential *in vivo* toxicity based on their *in vitro* bioactivity profile is an important step toward animal-free testing. A compendium of reference chemicals and data describing their bioactivity on specific molecular targets, cellular pathways, and biological processes is needed to bolster confidence in the predictive value of *in vitro* hazard detection. Endogenous signaling by all-trans retinoic acid (ATRA) is an important pathway in developmental processes and toxicities. Employing data extraction methods and advanced literature extraction tools, we assembled a set of candidate reference chemicals with demonstrated activity on ten protein family targets in the retinoid system. The compendium was culled from Protein Data Bank, ChEMBL, ToxCast/Tox21, and the biomedical literature in PubMed. Finally, we performed a case study on one chemical in our collection, citral, an inhibitor of endogenous ATRA production, to determine whether the literature supports an adverse outcome pathway explaining the compound’s developmental toxicity initiated by disruption of the retinoid pathway. We also deliver an updated Abstract Sifter tool populated with these reference compounds and complex search terms designed to query the literature for the downstream consequences to support concordance with targeted retinoid pathway disruption.

## 1 Introduction

Opportunities exist for refining and supplanting current developmental and reproductive toxicity (DART) testing protocols with *in vitro* data and *in silico* models that advance alternatives to animal testing (Scialli et al., 2018). The term “new approach methods” (NAMs) has been recently adopted in reference to any non-animal technology, methodology, approach, or combination thereof that can be used to provide information on chemical hazard and risk assessment that reduces the use of intact animals (USEPA, 2018).

Regulatory interest in the retinoid system is growing because of the increasing recognition that the retinoid pathway plays a critical role in many biological functions, especially during embryofetal development, and because of the growing number of environmental chemicals suspected to disrupt the pathway (Grignard et al., 2020; Mark et al., 2009; Duester, 2008). In 2012, the OECD Test Guidelines Programme in a Detailed Review Paper (DRP 178) identified a need for harmonized regulations on the retinoid system for toxicity screening and evaluation. The Endocrine Disruption Testing and Assessment Advisory Group (EDTA AG) initiated work on a DRP to review knowledge on the retinoid signaling pathway in multiple organ systems, which was subsequently narrowed to four areas: Overview, Reproductive System (Annex A), Skeletal Patterning (Annex B), and CNS Development (Annex C) (OECD, 2021, 2014). Currently, there are no validated assays in OECD guidelines probing the retinoid system despite its critical role in development. The ongoing work has resulted in a number of publications (Knudsen et al., 2021; Grignard et al., 2020; Damdimopoulou et al., 2019; Chen, H. et al., 2020). Researchers who develop or employ NAMs for DART testing of the retinoid pathway will need a comprehensive set of reference chemicals for testing and to vet and establish confidence in their assays (Judson et al., 2019). Here, our primary research aim was to compile information from the open literature and public databases to build a collection of chemical compounds with demonstrated activity against key targets in the retinoid pathway. The compilation is delivered as a list of compounds and assays linked to the publications in which they were identified. The results will serve as a resource for researchers developing NAMs for the retinoid system.

**Fig. 1:**
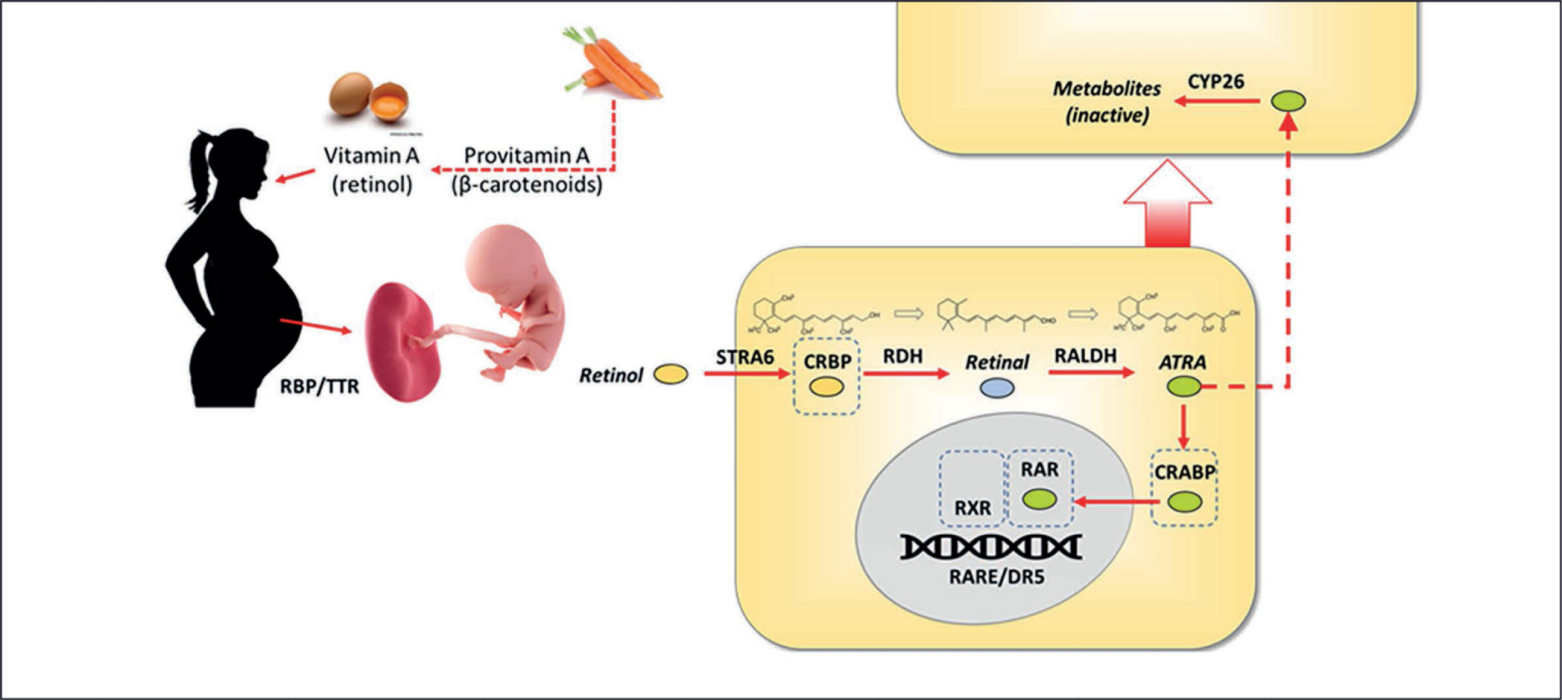
Diagram of retinoid pathway in human pregnancy In development, dietary Vitamin A (retinol) is transferred to the fetus through the placenta into the bloodstream. Dehydrogenase enzymes (RDH and RALDH) transform retinol to retinal and then to the active molecule ATRA. Transport proteins such as STRA6, CRBP, and CRABP move retinol and its forms (retinal, ATRA) from the bloodstream into the nucleus where ATRA binds the retinoic acid receptor / retinoid X receptor complex to initiate gene transcription.

While much retinoid toxicity research focuses on functional disruption of signaling by all-trans retinoic acid (ATRA), this work surveys ten significant targets in the retinoid pathway (Fig. 1, Tab. 1). The proteins and enzymes that mediate retinoid transport, metabolism, and transcription can influence levels of ATRA activity, thereby regulating – or dysregulating – other key pathways in development (Metzler and Sandell, 2016).

In the blood, dietary retinol (vitamin A) circulates bound to a complex containing retinol binding protein (RBP, sometimes referred to as plasma or serum retinol binding protein) and transthyretin (TTR – *transporter* of *thyroxin* and *retinol*) (Mujawar et al., 2014). This complex breaks apart when retinol leaves the complex at the cell surface to bind to STRA6, the cell membrane receptor stimulated by retinoic acid 6, for transport into the cell. STRA6 plays dual physiological roles as a transporter and a cell surface receptor that upon binding sets off a signaling cascade (Berry et al., 2012). Once in the cell, retinol is bound by cellular retinol binding proteins (CRBPs) (Kelly and von Lintig, 2015; Noy, 2016; Napoli, 2016). CRBPs regulate retinoid biology through dual intracellular functions, both as a transport mechanism and as a sink or storage for retinol (Napoli, 2017).

Before it can become biologically active, retinol must be transformed first to retinal and then from retinal to ATRA (Fig. 1). The first step is performed by enzymes in the alcohol dehydrogenase (ADH) family, specifically retinol dehydrogenase (RDH) members. The second oxidation step in the retinoid pathway converts retinal (retinaldehyde) to ATRA, the active signaling molecule. The metabolism of aldehydes – endogenous and exogenous – is the role of enzymes in the aldehyde dehydrogenase (ALDH) superfamily, specifically the retinal/retinaldehyde (RALDH) forms.

In the cytoplasm, cellular retinoic acid binding proteins 1 and 2 (CRABP1 and CRABP2) bind ATRA with high affinity and deliver this molecule to the nucleus. It is thought that CRABP1 shuttles ATRA to metabolic enzymes (e.g., cytochrome P450s) to buffer against ATRA excess, and CRABP2 transports ATRA into the nucleus and delivers it to the RAR/RXR receptor complex to regulate gene expression (Napoli, 2017; Wei, 2016).

Retinoic acid 4-hydroxylases comprise a subfamily of cytochrome P450 enzymes that break down ATRA. The three known isoforms, CYP26A1, CYP26B1, and CYP26C1, all metabolize ATRA efficiently but differ in their tissue localization. In the developing embryo, they exhibit differential cell-specific developmental regulation (Helvig et al., 2011), an action that in part accounts for regional ATRA gradients.

The three retinoic acid receptor forms are alpha (RARa), beta (RARb), and gamma (RARg). All isoforms heterodimerize with the retinoid X receptors (RXR) to regulate transcription at the retinoic acid response element (RARE) binding sites in ATRA-responsive genes. There is evidence for at least 27 genes under direct control of the RAR/RXR complex and many more under indirect control (Balmer and Blomhoff, 2002; Grignard et al., 2020). Compounds that bind one of the RAR receptors often bind one or more of the other isoforms.

The RARs form dimers with the RXRs, and while this activity is critical to downstream gene expression in the retinoid pathway, RXRs also form dimers with other receptors, e.g., peroxisome proliferator-activated receptors (PPAR), liver X receptors, and farnesoid X receptors (Brtko and Dvorak, 2020; Knudsen et al., 2021). Because of the toxicological and metabolic complexity of RXR biology, RXRs were not considered in-scope for this work in order to focus on the RAR-mediated pathway alone. Other molecules play roles in the retinoid pathway and could arguably be included in Figure 1 and this study. Metabolizing enzymes such as the cytochrome P450 isoforms CYP1A1 and CYP1B1 and glucuronosyltransferases have been linked to ATRA metabolism (Rowbotham et al., 2010; Maguire et al., 2020; Li, D. et al., 2017). These enzymes are less studied in the context of the retinoid pathway and, except for including CYP1A1 ToxCast results, they have been omitted from this work.

While reference chemicals are key to building confidence in *in vitro* assays (Judson et al., 2019), establishing confidence in the reference chemicals is also important. For example, evidence that a compound known to disrupt a protein target in the retinoid pathway also causes the adverse effects associated with altering that target activity will increase the confidence in that chemical as a potential reference compound in retinoid assays. In other words, demonstrating that a chemical not only shows activity in an *in vitro* assay on a target in the retinoid system but that the chemical has evidence linking it to the other key events and outcomes in an adverse outcome pathway (AOP) will bolster confidence in the chemical as a retinoid disruptor (Villeneuve et al., 2014).

**Fig. 2:**
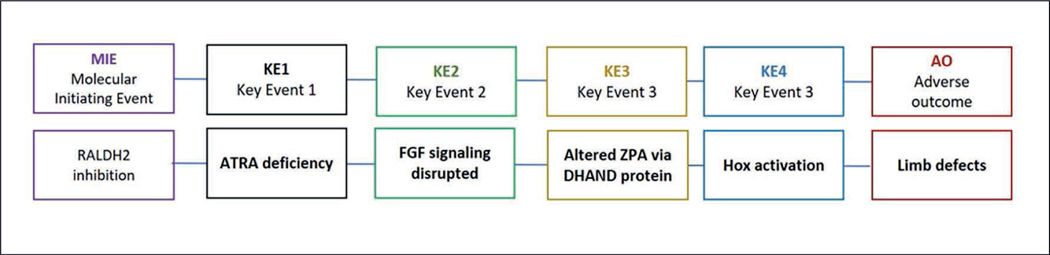
Provisional AOP for limb defects caused by RALDH2 inhibition The molecular initiating event (MIE) is inhibition of RALDH2, which in turn decreases the synthesis of ATRA (Key Event or KE), followed by disruption of the balance in the fine-tuned gradients of ATRA and its signaling antagonist FGF8 in the limb bud (Knudsen et al., 2021). The imbalance affects the zone of polarizing activity (ZPA) and expression of DHAND (heart and neural crest derivatives expressed) protein and is followed by alterations in RARa/RARg regulated expression of Homeobox genes (*Hox*) (Niederreither et al., 2002). The imbalance disrupts conditions for normal limb growth, particularly digit formation.

Limb development is one of the developmental processes in which the retinoid pathway is crucial, and disruption of that pathway causes defects. ATRA gradients direct the morphology of the developing limb where a precisely orchestrated interplay of ATRA and fibroblast growth factor (FGF) gradients control the normal development of the stylopod, zeugopod, and autopod regions (Knudsen et al., 2021). Exogenous ATRA disrupts this gradient balance and causes limb defects, including phocomelia and digital defects in mice, rats, and chicks (Kochhar, 1973; Wiley, 1983; Yu et al., 2003). Excess ATRA caused by absence or down-regulation of the ATRA metabolizing enzyme CYP26 also has limb effects. Cyp26b1^−/−^ mutant mice lack the CYP26 enzyme that eliminates ATRA, causing excess levels of the morphogen. These mice exhibit multiple forelimb, hindlimb, and digit deformities (Yashiro et al., 2004). Too little ATRA in locations where it is required is also teratogenic (Lee, G. S. et al., 2004). Reducing synthesis of ATRA through imbibition of RALDH2 has been associated with limb defects: Mouse knockouts of RALDH2 result in several abnormal limb phenotypes including small or absent forelimb and hindlimb buds, abnormal digits, and syndactyly (Niederreither et al., 2002; Vermot et al., 2005). We previously assembled a provisional AOP describing a pathway starting with RALDH2 down-regulation and leading to limb defects as the adverse outcome (Fig. 2).

A chemical with a substantial history of use or environmental exposure may indeed have other published supporting evidence of its molecular activity, including effects on cells, tissue, organs, and adverse outcomes in an intact organism or population. Our second aim was to gather and assess the literature support for our chemical set causing adverse outcomes associated with retinoid disruption. We draw connections between the chemicals and outcomes, first broadly by querying the biomedical literature for areas of developmental toxicity co-occurring with the candidate reference chemicals, and then we focus on one chemical, citral, an inhibitor of endogenous ATRA production, to perform an in-depth literature review looking for evidence that the chemical participates in an AOP linking RALDH inhibition to developmental limb defects through the steps illustrated in the AOP in Figure 2.

## 2 Methods

The molecular targets in the retinoid pathway that are the focus of this work are listed and briefly described in Table 1. While the primary interest is human data, information on model organisms from different vertebrate species is considered.

### Databases mined for chemical retinoid system disruptors/modulators

The information on the retinoid system is abundant – a search using the term “retinoid” in PubMed returns over 64,000 entries – and each of the proteins and enzymes in the retinoid pathway has substantial literature on its own. To make the project tractable, we started with structured data in publicly available databases that describe *in vitro* assay results of a chemical’s activity against one of the pathway targets. These databases included Protein Data Bank (PDB) (Berman et al., 2003), ChEMBL (Gaulton et al., 2017), and ToxCast/Tox21 (Richard et al., 2016; Judson et al., 2010). PDB is a repository of protein structures, often bound with small molecule ligands. Some ligands are present to stabilize the protein for crystal formation, but sometimes the researchers test potential drug candidates for binding specificity against a target. ChEMBL is a source of assays and chemical data curated from publications, and ToxCast/Tox21 is a repository of high-throughput assay data. We elected not to use PubChem at this point because its data are deposited by contributors and not curated (Kim, S. et al., 2019). The other source of information was literature occurrences of chemical-target interactions in PubMed’s Medical Subject Heading (MeSH) terms. Database retrieval was performed originally in 2020 and reviewed for additional records in May 2022.

For each target, we queried PDB (Berman et al., 2003) using the protein name or official identifier as a query term. Results from PDB were downloaded and are presented in Table S1^1^. To condense these results, some information was summarized or excluded (e.g., the chain information for each entry). Hyperlinks for the PDB Identifier (PDB ID) and the PubMed Identifier (PMID) literature record are included in the table. If multiple ligands were used for crystallization, the chemical names are separated by a forward slash (/).

ChEMBL was also queried for each target (Gaulton et al., 2017). Resulting assay records were downloaded using web services and formatted into Table S2^1^. The table contains the assay description and hyperlinks to the assay at ChEMBL and to PubMed when a PMID is provided.

When available for a target, the ToxCast and Tox21 assays were downloaded and included in this study (Richard et al., 2016; Judson et al., 2010). The ToxCast/Tox21 portfolio includes > 600 high-throughput screening (HTS) *in vitro* assays for a wide variety of biochemical and cellular responses, including three retinoid pathway receptor targets and the retinoid pathway (described in Tab. S18^1^). The ToxCast/Tox21 chemical universe comprises environmental toxicants such as pesticides as well as chemicals in consumer products, cosmetics, and pharmaceuticals (Richard et al., 2016). These data were downloaded from the EPA CompTox Chemicals dashboard^2^ (Williams et al., 2017) in December 2019. In this database, bioactivity is recorded as a “hit” based on AC50 (concentration resulting in 50% change in maximum activity observed) or related measurements, as well as automated concentration curve responses generated in the ToxCast data pipeline (Filer et al., 2017). For this work, we limited hits to those chemical-assay pairs where AC50 < 2.0 μM to allow focus on the most potent chemicals. Next, each record was reviewed and curated to remove equivocal hits. We inspected the cytotoxicity values and caution flags associated with each automated hit call and included only hits that were associated with fewer than three caution flags (Filer et al., 2017; Judson et al., 2010). On the EPA CompTox Chemicals Dashboard, these flags are found below the curve charts for each chemical-assay pair, and the cytotoxicity cutoff line is colored red. The chemicals remaining after the curation step were inserted into tables with the AC50 values and chemical identifiers used by the EPA CompTox Chemicals Dashboard (DSSToxIDs) hyperlinked to the dashboard. For the retinoid pathway assays in Tox21, in addition to the filtering steps applied to all ToxCast/Tox21 results, additional data quality filters were applied to remove activity hit calls flagged for questionable quality. Results retained were for compounds active at concentrations below the cytotoxicity cutoff that had fewer than three caution flags and did not have either of the caution flags: “Less than 50% efficacy” or “AC50 less than lowest concentration tested”.

Next, the EPA’s literature database of MeSH terms extracted from PubMed (Judson et al., 2019; Baker and Hemminger, 2010) was queried for chemical annotations associated with the MeSH annotations of the protein targets. These target-chemical annotation pairs were identified and exported to an Excel workbook^3^ and subsequently reviewed and curated for chemicals of interest.

### Connecting the chemicals to the biological literature

The target-chemical sets from each of these retrieval steps were inserted into the PubMed Abstract Sifter^4^ literature mining tool (Baker et al., 2017) CuratedChemicals sheet. Using the batch search capability of the EPA CompTox Chemicals Dashboard, the DSSTox identifier for each chemical was retrieved and added as a hyperlink. A query string consisting of the chemical name, CAS-RN, and MeSH synonym (if found) were added to the sheet. The functionality of the Abstract Sifter is described in the user guide^4^ in detail and will not be reproduced here.

A previous study compiled potential AOPs that cause skeletal defects from retinoid pathway disruption during embryonic development (Knudsen et al., 2021). We built PubMed queries designed to retrieve the citations describing each of these AOP key event’s biological activity. For example, the complex query designed to find citations describing limb defects reads “(Limb deformities, Congenital[tw] OR limb defects OR polydactyly OR Brachydactyly OR digits OR Fingers/abnormality OR toes/abnormality OR limb bud/drug effects OR Ectromelia OR amelia OR hemimelia OR phocomelia OR sirenomelia)) AND (embryo OR fetus OR fetal OR embryonic)”. The queries for each step in the skeletal disruption AOPs were stored on the Pathway_Queries sheet of the Abstract Sifter.

The connections between the chemical corpus and the literature describing biological activity were explored using the Landscape sheet of Abstract Sifter. Chemical entities were copied from the CuratedChemicals sheet to the Landscape sheet column C, and pathway queries were copied to the Landscape sheet into Row 3. The article counts resulting from queries composed of the chemicals and the biological queries were retrieved and results sorted.

### Citral case study and AOP for developmental limb defects

We selected one chemical – citral – and performed a case study to determine whether there was literature evidence to support a complete putative AOP starting with RALDH inhibition and leading to developmental limb defects. Citral was chosen because it has been used for several decades in experiments to disrupt the retinoid pathway through blocking RALDH (Connor and Smit, 1987; Kikonyogo et al., 1999). Composed of two isomers, nerol and geraniol, citral is a naturally occurring substance in the oils of several plants such as lemon verbena. The first article about citral in PubMed, describing the application of citral in glaucoma, was published in 1949 (Kaminskaia, 1949). Since then, there have been articles describing its potential use in cancer, glaucoma, parasitic diseases, atherosclerosis, fungal diseases, and as an insect repellant. It has been tested for safety because it is commonly used in perfumes and cosmetics for its lemon aroma. Because of its many uses, citral has accumulated a substantial literature presence. The Abstract Sifter literature mining tool (Baker et al., 2017) was used to search PubMed for literature evidence connecting citral to each of the key events in the putative developmental AOP in Figure 2.

## 3 Results

The candidate reference chemicals identified in the following work can be found in the accompanying Abstract Sifter tool^5^ organized by target on the RefChemSet sheet, in a simple list on the AllChems sheet, and on the Landscape sheet, shown with sample toxicity-related queries and resulting article counts.

### 3.1 Collection of data on the specific targets in the retinoid pathway

#### 3.1.1 Retinol binding protein – serum/plasma (RBP)

The structure of serum RBP has been studied extensively, resulting in many PDB entries, which are summarized in Table S1^1^. Some of these crystal structures include transthyretin and retinol; retinoids are the most commonly used ligands. ChEMBL contains 22 assays for serum RBP curated from seven publications. Descriptions of these assays are given in Table S2^1^. Most of the assays are binding assays against recombinant human serum RBP. Researchers interested in RBP as a potential therapeutic target have identified retinoid and non-retinoid (without retinoid structure) compounds that bind RBP and inhibit its activity (Cioffi et al., 2014; Wang, Y. et al., 2014). Retinol is the physiological ligand and is the *de facto* reference chemical in many assays. Other chemicals of interest are described below.

Fenretinide is a synthetic retinoid used as a positive control in some ChEMBL studies. This drug disrupts the RBP-TTR complex by competing with retinol in binding to RBP. Because the drug is associated with a number of side effects, other non-retinoids that bind RBP have been sought (Racz et al., 2018). Compound A1120 was one such non-retinoid found to bind RBP in a way that disrupted the association and interaction of RBP and TTR. It was originally developed as a treatment for diabetes, but has more recently been studied as a potential treatment for macular degeneration (Cioffi et al., 2014; Hussain et al., 2018; Wang, Y. et al., 2014). With A1120 as a starting point, a set of compounds was rationally designed and tested by Racz and colleagues for RBP activity, leading to the identification of BPN-14136, a non-retinoid that effectively binds RBP, disrupts the RBP-TTR complex, and thus blocks the *in vivo* delivery of retinol to cells (Racz et al., 2018). Additionally, in studies in women with gestational diabetes, the drug sitagliptin has been found to downregulate RBP protein levels (Sun et al., 2017).

#### 3.1.2 Receptor stimulated by retinoic acid 6 (STRA6)

STRA6 structure is well-described and its function has been characterized in assays testing for the uptake of retinol into cells (Breen et al., 2015; Chen, Y. et al., 2016a; Kawaguchi and Sun, 2010). The PDB entries investigate STRA6 (Tab. S3^1^) bound to endogenous substances, primarily the protein calmodulin. The binding of STRA6 and calmodulin is thought to be an important regulatory step of the retinoid pathway and a potentially important target for pharmaceuticals (Zhong et al., 2020).

Mutations in *Stra6* are associated with severe developmental defects (Pasutto et al., 2007) and diseases such as diabetes (Chen, C. H. et al., 2019); however, there are no records in ChEMBL or PubChem for STRA6 nor literature connecting it with drug therapies or chemical toxicity.

#### 3.1.3 Cellular retinol binding proteins (CRBP)

CRBP has two main forms with the official gene symbol *Rbp1* (commonly reported as CRBP1 or CRBP-I) and *Rbp2* (commonly reported as CRBP2 or CRBP-II). Their structure has been explored through crystal structures and other methods. PDB has over 50 records for CRBPs (Tab. S4^1^); however, there is less information on CRBP1 and CRBP2 binding data or functional assays in ChEMBL or ToxCast datasets.

In the PDB entries, retinol and retinal are the most common ligands. Recently, interest has grown in CRBP as a pharmacological target, particularly in ocular disorders and diseases. While retinoid derivatives are often tested (e.g., retinylamine), the search for analogues or antagonists has focused on non-retinoid compounds to avoid adverse effects associated with retinoids. High-throughput screening (HTS) methodology was used to measure displacement of retinol from CRBP1 to test a library of over 900 compounds (Silvaroli et al., 2019). A cannabidiol derivative called abnormal cannabidiol (abn-CBD) was identified as a potent ligand and inhibitor of CRBP1 (6E5L), and its derivatives such as cannabidioricin (6E6M) and 2-AG (2-arachidonoylglycerol) were also strong ligands. In recent work, a series of bioactive lipids was found to bind to CRBP2 with an affinity comparable to retinol, including the endocannabinoids 2-arachidonoylglycerol, 2-arachidonoylglycerol, 2-oleoylglycerol, and 2-lineoylglycerol (Lee, S. A. et al., 2020; Silvaroli et al., 2021).

#### 3.1.4 Cellular retinoic acid binding protein (CRABP1 and CRABP2)

While the structures of CRABP1 and CRABP2 are similar, their localization and functions appear to be distinct (Donovan et al., 1995; Ruberte et al., 1992; Wei, 2016). This distinction holds in the differing associations between the proteins and diseases, particularly cancer (Favorskaya et al., 2014). Whereas elevated CRABP1 is associated with poor outcomes in breast cancer (Liu, R. Z. et al., 2015), elevated CRABP2 has the opposite correlation. In some cancers, such as Wilms tumors, the pattern is reversed and a poor outcome is associated with high CRABP2 (Takahashi et al., 2002).

The associations between each CRABP form and disease have motivated the search for compounds that specifically bind each protein. PDB has many entries for CRABP2 and some for CRABP1 (Tab. S5^1^), and ChEMBL has assays for both proteins (Tab. S6^1^). The compounds tested have mainly been new synthetic retinoids. With the identification of these compounds, researchers hope to find drugs that have some of the activity of ATRA, but without the adverse outcomes, particularly teratogenesis. Fogh et al. (1993) measured the binding affinity of a series of retinoid compounds against CRABP1 and CRAPB2. The active compounds’ results are listed in Table 2. The binding affinity of a series of retinoids to CRABP1 and CRAPB2 (Chaudhuri et al., 1999) is also summarized in Table 2. The chemical set includes AM80 (tamibarotene), a known RAR alpha agonist (Kagechika et al., 1988) that is available commercially and was tested for chemotherapeutic efficacy (Anonymous, 2004). The set also includes TTNPB, a synthetic retinoid that is more active than ATRA and has been used in the mouse limb bud assay (Pignatello et al., 1997). There are ChEMBL data for isotretinoin and alitretinoin in addition to newly synthesized compounds with undefined names. Finally, 4-amino-2-trifluoromethyl-phenyl retinate is an ATRA derivative that has been used in cell assays (Wang, B. et al., 2013) and was found to regulate CRABP2 (Ju et al., 2018).

#### 3.1.5 Retinoic acid 4-hydroxylases (CYP26 subfamily)

Retinoic acid 4-hydroxylases comprise a subfamily of cytochrome P450 enzymes that break down ATRA. The three known subforms, CYP26A1, CYP26B1, and CYP26C1, all metabolize ATRA efficiently but differ in their tissue localization. In the developing embryo, they exhibit differential cell-specific developmental regulation (Helvig et al., 2011), an action that in part accounts for regional ATRA gradients.

Recent interest in the CYP26 subfamily has focused on pharmacological manipulation of endogenous retinoids in the treatment of a number of diseases, particularly cancer (Bruno and Njar, 2007; Thatcher et al., 2011). PDB has no structural entries for any of the CYP26 forms, although its primary genomic structure has been investigated (Foti et al., 2016a). ChEMBL has 29 assay entries for CYP26A1 and two entries for CYP26B1, summarized in Table S7^1^. The most common active chemicals were liarazole, talarozole, ketoconazole, fenretinide, and bexarotene. The azole ring system is a common feature among compounds that disrupt ATRA signaling at CYP26 but lack a typical retinoid chemical structure.

A survey of the literature reveals other interesting assays. Helvig et al. (2011) developed a binding assay for the binding pocket of CYP26A1, CYP26B1, and CYP26C1 that allowed comparison of affinity measures for ATRA, 9-cis-retinoic acid, 13-cis-retinoic acid, and ketoconazole. Thatcher et al. (2011) evaluated a set of 42 compounds for activity against CYP26A1 at two concentrations. The 10 most potent inhibitors, including the three azoles talarozole (R115866), liarozole, and ketoconazole and the PPAR agonists pioglitazone and rosiglitazone, are listed in Table 3. Three other azoles, itraconazole, fluconazole, and voriconazole, were only inhibitory at the higher concentration tested. Foti et al. (2016a) used a homology model to predict how CYP26 would metabolize the drug tazarotenic acid (Agn 190299) and were able to support their prediction by measurement. Based on the observation that the binding region of CYP26 is similar to that of CYP2C8, they tested a set of 29 known CYP2C8 inhibitors against CYP26A1 and CYP26B1. The IC50 was determined for the 17 compounds that showed greater than 50% inhibition at 10μM. Clotrimazole was the most potent inhibitor tested. For the tested compounds as a whole, there was a positive and statistically significant correlation between CYP26A1 and CYP2C8 IC50 values and only a weak correlation between CYP26B1 and CYP2C8 (Foti et al., 2016b). Chemicals used as reference compounds in the Foti studies and those that passed the 50% inhibition screen are shown in Table 3.

The Foti work is interesting because, although ToxCast does not contain an assay for CYP26, it does contain one for CYP2C8. CYP2C8 is a potential surrogate for CYP26A1 and CYP26B1 bioactivity. CYP1A1 has also been shown to metabolize ATRA (Lampen et al., 2000). Results from ToxCast assays for CYP2C8 and CYP1A1 are included on Table S17^1^.

#### 3.1.6 Retinol dehydrogenase (RDH)

RDHs are alcohol dehydrogenases (ADH) and therefore members of the short-chain dehydrogenase/reductase family of enzymes (SDR). The SDR family is large, with over 46,000 members in all species; in humans, 70 genes have been identified in the superfamily (Persson et al., 2009). The historical nomenclature is bewildering: names have changed over the years and are still not firm (Napoli, 2020). SDR enzymes often share substrates, and members of the ADH subfamily can play a role in the oxidation of more than one alcohol-containing chemical, with varying affinity (Kumar et al., 2012; Persson et al., 2009; Wang, C. et al., 2011). It is thought that while *in vitro* retinol is a substrate for many ADH forms, *in vivo* the retinol dehydrogenases are the only form that can act on retinol bound to RBP (Napoli, 2020). A table from Napoli (2012) of the enzymes in mouse, rat, and human that contribute significantly to the retinol dehydrogenase step in ATRA biosynthesis is reproduced in Table 4.

PDB and ChEMBL nomenclature reflects the fact that RDH is often referred to as alcohol dehydrogenase Class IV in the literature. In PDB, a search using this term resulted in two entries, both from a publication describing the binding of 4-methylpyrazole to the class IV (retinol dehydrogenase) isoform of human alcohol dehydrogenase (Xie and Hurley, 1999). ChEMBL assays found by searching for “retinol dehydrogenase” surfaced over 10 results from the SDR family that act on alcohols nonspecifically and can dehydrogenate retinol to some extent. Focusing on alcohol dehydrogenase class IV brought up one publication that studied a set of formamide derivatives (Schindler et al., 1998) and highlighted N-heptylformamide for its potency and compared it to all-trans-retinol.

Alcohols, and specifically ethanol, have received attention for their metabolism by the dehydrogenase/reductase family. A review of the ChEMBL publications indicates that inhibitors of these enzymes are sought to counteract the toxic effects of alcohol, and therefore the assays focus on liver enzymes (Chen, W. S. et al., 1981; Schindler et al., 1998; Venkataramaiah and Plapp, 2003). Because of the similarity between the phenotype associated with fetal alcohol syndrome and the phenotype linked to vitamin A deficiency (VAD), ethanol has been studied for its effect on retinol oxidation. Mammalian alcohol dehydrogenases from multiple families accept ethanol and retinol as substrates, and studies show that ethanol can competitively inhibit alcohol dehydrogenases, leading to a decrease in retinal and ultimately ATRA production (Duester, 1991; Molotkov and Duester, 2002). Ethanol will continue to be a topic in the next section of this work. The search for drugs to treat alcohol abuse powers much of the *in vitro* research on ALDH. In addition to ethanol, other compounds studied for effects on retinol or alcohol dehydrogenases are summarized below.

Cimetidine was found to be a competitive inhibitor of human class IV ADH when tested against ethanol (Allali-Hassani et al., 1998), the fungicide ziram was found to weakly inhibit rat RDH2 (Su et al., 2018), resveratrol inhibited rat RDH2 when tested against steroid substrates (Wang, Y. et al., 2017), and the insecticide methoxychlor and its metabolite hydroxychlor (HPTE) both inhibited rat RDH2, with HPTE being the more potent compound (Mao et al., 2018). RDH2 has been shown to be inhibited by gossypol, a compound that disrupts male reproduction (Cao et al., 2019; Lim et al., 2019). The compounds carbenoxolone and phenylarsine oxide have been used *in vitro* to inhibit RDH (Napoli, 2020; Boerman and Napoli, 1995).

In a study published in 1969, pyrazole and pyrazole derivatives were tested against an unnamed form of ADH, and the oxidation of both ethanol and retinol was inhibited (Reynier, 1969), indicating that pyrazole inhibits ADH. Pyrazole is a ring structure found in many pesticides and drugs. In subsequent work, 4-methylpyrazole was used in a number of *in vitro* studies to block ADH (Galli et al., 2001). In one study, 4-methylpyrazole ameliorated the toxic effect of retinol on embryonic mice (Collins et al., 1992). 4-Methylpyrazole is also known as fomepizole and is available as a drug treatment for methanol and ethylene glycol overdose (Thanacoody et al., 2016).

#### 3.1.7 Retinal dehydrogenase (ALDH, RALDH)

In humans, the ALDH superfamily contains 19 isoenzymes expressed in different tissues at different times that accept a variety of substrates. While it is thought that each member of the family has a preferred substrate, each will accept other substrates with lower affinity (Koppaka et al., 2012). In the developing embryo, ALDH1A2 (also known as RALDH2 for retinal dehydrogenase) is the major retinal dehydrogenase responsible for the conversion of retinal to ATRA during early gestation. ALDH1A1 (RALDH1) and ALDH1A3 (RALDH3) come into play later during facial morphogenesis (Metzler and Sandell, 2016). In general, ALDH enzymes break down compounds from the aldehyde form to the less toxic acid form. Table 5 (adapted from Koppaka et al., 2012) lists ALDH family members with relevance to retinoids.

The records in PDB for ALDH1A1, ALDH1A2, and ALDH1A3 reflect the search for new chemical entities specific for these enzymes (Tab. S9^1^). Morgan and Hurley developed an assay that identifies selective inhibitors of ALDH1A1 including two distinct chemical classes (Morgan and Hurley, 2015). The structure of ALDH1A2 was studied using crystallography, and binding studies were performed against a range of compounds, including WIN18,446 (Chen, Y. et al., 2018). PDB contains one record for ALDH1A3 describing its structure bound to ATRA (Moretti et al., 2016).

The 72 assays deposited in ChEMBL are summarized in Table S10^1^. Many of the studies were motivated by the need to modulate the retinoid pathway for disease treatment. Specific inhibitors have been sought for ALDH1A1, ALDH1A1, and ALDH1A3 for treatment of Parkinson’s disease, obesity, cataracts, and various types of cancer (Huddle et al., 2018; Morgan and Hurley, 2015; Quattrini et al., 2020). The ChEMBL collection includes high-throughput assays conducted by the National Center for Advancing Translational Sciences (NCATS). Originally deposited in PubChem, over 220,000 chemicals were evaluated for inhibition of ALDH1A1 activity in an *in vitro* assay (Yasgar et al., 2017).

The links between the retinoid pathway and ethanol are strong, both in the literature about embryonic development and in the literature about treatments for alcohol abuse. Ethanol is known to be toxic to the developing fetus. With ethanol, the first oxidation step transforms alcohol to acetaldehyde, the compound acted upon by ALDH in the second oxidation step to form acetic acid. When this second oxidation is blocked by inhibition of ALDH or by genetic variations in ALDH2 that render it incapable of performing the transformation, acetaldehyde builds up and causes alcohol flushing syndrome in humans, a condition characterized by flushing, shakiness, nausea, and tachycardia. The drug disulfiram (trade name Antabuse) was designed to inhibit ALDH and cause alcohol flushing syndrome with the goal of causing patients to avoid drinking (Bell and Smith, 1949; Jacobsen and Larsen, 1949). The search for new treatments for alcoholism has energized research into ALDH to identify compounds that specifically target certain isoforms (Koppaka et al., 2012). Disulfiram has been shown to inhibit the retinal dehydrogenase ALDH1A1 as well as ALDH2 (Jin et al., 2018; Kim, Y. J. et al., 2017). When signs of alcohol flushing syndrome are observed following (non-ethanol) chemical exposures in patients, ALDH inhibition is suspected (Sharma et al., 2009; Plouvier et al., 1982; Garnier et al., 1992; Finulli and Magistretti, 1961).

A link between disulfiram and cancer treatments has also been established, resulting in an increase in publications describing the drug as a cancer therapeutic by itself (Liu, P. et al., 2012; Lu et al., 2021; Zhang, J. et al., 2020) or as an adjunct therapy for chemotherapeutics (Kast and Belda-Iniesta, 2009). ALDH activity protects cancer cells from the effects of therapeutics and contributes to drug resistance. As an adjunct therapy, disulfiram blocks ALDH, making the cancer cells less likely to develop resistance to treatment (Wang, N. N. et al., 2018; Raha et al., 2014). This approach has stimulated the testing of further compounds in ALDH assays (Thomas et al., 2016).

Koppaka et al. (2012) reviewed inhibitors of ALDH. Table 6 summarizes their findings on significant inhibitors of the retinal dehydrogenases from the literature. A number of the compounds require metabolic activation, and the manuscript should be consulted for more detail on this.

A number of pesticides in the thiocarbamate family share structural features with disulfiram and its metabolites and have also been shown to inhibit ALDH. Quistad et al. (1994) tested a set of thiocarbamate pesticides in *in vitro* ALDH binding assays and *in vivo* assays measuring the effects on acetaldehyde levels in mice. They found some of the pesticides had activity similar to the ALDH inhibitor disulfiram. The publication has the full list of chemicals tested and their percent inhibition of liver ALDH. Among the most potent inhibitors were EPTC, thiobencarb, pebulate, vernolate, and molinate. Experiments testing molinate and its metabolites for their relative inhibitory potency in mouse and human *in vitro* models indicated molinate sulfone was the most potent ALDH inhibitor (Allen et al., 2010). These experimental results are supported by the observation that agricultural workers exposed to these compounds develop an intolerance to alcohol (Quistad et al., 1994).

DEAB (diethylaminobenzaldehyde) is used as a control in the Aldefluor assay (Stemcell Technologies, Inc.) to test for aldehyde activity associated with cancer stem cells. Its selectivity against the ALDH family members has been studied to establish its substrate profile (Morgan et al., 2015) revealing that it is a substrate for ALDH3A1, ALDH1A1, ALDH1A3, ALDH1B1, and ALDH5A1.

The natural product citral has been known for many years to be an aldehyde dehydrogenase inhibitor, and it has routinely been used to block ATRA synthesis in the laboratory (Xu et al., 2018; Kikonyogo et al., 1999).

Win18,446 is shown bound to ALDH1A2 in the PDB records. This chemical, also known as bisdiamine, was long known to be a spermatogenesis inhibitor (Kar et al., 1966). Further studies linked Win18,446 to other reproductive and adverse developmental effects (Oster et al., 1974; Momma et al., 1990; Singh and Dominic, 1995). Mey et al. (2003) showed that this compound, along with the herbicide nitrofen, 4-biphenyl carboxylic acid (BPCA), and SB-210661, caused congenital diaphragmatic hernias in rats and established *in vitro* inhibition of retinal dehydrogenase.

#### 3.1.8 Retinoic acid receptor alpha (RARa)

The structure of RARa has been studied extensively. PDB has data on RARa that include the protein complexed with agonists, antagonists, and RXR (Tab. S11^1^). ChEMBL contains over 250 assays for RARa binding and functional assays (Tab. S12^1^). A review of the PubMed publications behind the depositions to ChEMBL showed that most are studies of newly synthesized compounds designed with the goal of optimizing compound activity. The natural ligand, ATRA, is commonly used as a reference compound. The ToxCast/Tox21 battery of assays includes three assays testing for RARa ligand binding and/or reporter gene transactivation (Tab. S17^1^). Selected chemicals of interest are discussed below.

Am580 and Am80 (tamibarotene) were synthesized in 1988 as synthetic retinoids and found to have potent activity as RARa agonists (Kagechika et al., 1988). These chemicals have been tested in many assays and studied as treatments in a number of diseases. BMS493 is an inverse agonist of RARa (Germain et al., 2009). AGN 193109 was identified as a potent RARa antagonist in 1995 (Johnson et al., 1995). Since then, it has been referenced in over 30 publications, where it is studied for use in disease treatment and as a reference compound for RAR binding assays. Developmental *in vivo* assays demonstrate that it causes craniofacial abnormalities (Kochhar et al., 1998). AGN 193109 has been referred to as an inverse agonist (Thacher et al., 1999) and has been shown to bind RARb as well as RARg (Agarwal et al., 1996). The compound is commercially available from many vendors. ALRT 1550 was identified as a novel agonist of RARa (Zhang, L. et al., 1996). It has received some attention as a potential treatment for cancer (e.g., Hu et al., 2002). The compound is also commercially available.

#### 3.1.9 Retinoic acid receptor beta (RARb)

RARb has 12 records in PDB (Tab. S13^1^). The early records reflect research into understanding the receptor and its endogenous ligand; later, synthesized compounds were tested for their potential as pharmacologically useful ligands. ChEMBL contains over 200 assays for RARb, which are summarized in Table S14^1^. The assays include agonist, antagonist, binding, and activation modes. ToxCast includes one applicable RARb assay, which is described in Table S18^1^, and the results are summarized in Table S17^1^. Selected chemicals are discussed below.

Benzo[a]pyrene diol epoxide (BPDE) is a metabolite of benzo[a]pyrene, a combustion by-product of several processes including cigarette smoking. This commercially available compound is thought to inhibit RARb through suppression of the RARb promoter (Song and Xu, 2001). BMS453 is a RARb-specific agonist and a RARa antagonist (Chen, R. et al., 2001). CD 2019 is a RARb-specific agonist. In a study of the relative teratogenicity of three specific RAR agonists, CD 2019 was the RARb agonist used, while AM 580 was the RARa agonist, and CD 437 was the RARg agonist (Elmazar et al., 1996). LE 135 is a RARb antagonist and has been shown to affect the chondrogenic pathway in development (Li, Z. et al., 2011).

#### 3.1.10 Retinoic acid receptor gamma (RARg)

RARg, similarly to RARa and RARb, has received attention as a drug target. Researchers have sought compounds specific to RARg, often testing non-retinoid compounds to avoid the side effects associated with this structural family. RARg has 11 records in PDB (Tab. S15^1^), most of which were compounds synthesized as potential drug candidates. There are 13 assay records in ChEMBL (Tab. S16^1^) that include agonist, antagonist, and binding functionality. ToxCast has one assay for RARg specifically: ATG_RARg_TRANS_up. The top results in terms of highest potency for this assay are in Table S17^1^. Selected chemicals are discussed below.

BMS 961 is a selective RARg agonist (Klaholz et al., 1998). Identified as a ligand in 1998, the compound has been used as a test chemical in limb development assays (Galdones and Hales, 2008) and is commercially available. CD1530 has been used in several studies as a specific RARg agonist. It has also been shown to inhibit CYP26A1 with a potency similar to that of ketoconazole (Thatcher et al., 2011). Trifarotene (CD5789) is a recently synthesized RARg selective agonist that has been approved for the treatment of acne (Thoreau et al., 2018). Because the compound has undergone clinical trials, safety data are available (Blume-Peytavi et al., 2020; Tan et al., 2019). CD 437 has been used to induce apoptosis in a number of cancer cell types and was one of the three compounds studied for teratogenic effects (Shao et al., 1995; Elmazar et al., 1996).

#### 3.1.11 Collection of data on retinoid pathways

Several existing assays test activity of the retinoid pathway as a whole. These are generally reporter assays that measure some effect downstream from receptor binding such as gene transcription. In a mouse pluripotent P19 cell model, Chen and Reese (2013) tested a reporter assay designed to measure levels of *Hoxa1* expression, which plays an important role in development and whose transcription levels are controlled by retinol. These authors tested the assay using chemicals known to perturb various steps in the retinoid pathway (Tab. 7). In a subsequent publication, the authors performed the same assay on a set of phthalate esters (Chen and Reese, 2016). In more recent work, Chen, Reese and colleagues performed a RARE reporter assay in the C3RL4 mouse cell line using the Tox21 high-throughput screening platform and tested over 1000 chemicals (Chen, Y. et al., 2016b; Attene-Ramos et al., 2013). This assay was then implemented as part of the ToxCast/Tox21 platform, and while the data have not been analyzed and published yet, they can be accessed under the names TOX21_RAR_LUC_Agonist and TOX21_RAR_LUC_Antagonist. Another assay in ToxCast that measures the effects on the retinoid pathway is ATG_DR5_CIS_up, which monitors DR5 (direct repeats of 5 nucleotides) for RAR/RXR transactivation. This assay uses fluorescence intensity to indicate induction of RARE, a cis acting reporter response element responsive to RARa, RARb and RARg.

The top results in terms of highest potency for the TOX21_RAR_LUC agonist and antagonist assays and the DR5 assay are in Table S17^1^. The ToxCast DR5 results show effects for some organochlorine pesticides such as endrin, dieldrin, endosulfan, and chlordane, in addition to known retinoids. Lemaire et al. (2005) performed extensive assays on these compounds with the goals of delineating their activity at RARa, RARb, and RARg. They suggest that these compounds may disrupt the retinoid receptor by working as low-affinity agonists on RARa, RARb, and RARg and hypothesize that prolonged exposure to these compounds may contribute to teratogenicity.

### 3.2 Literature exploration and citral case study

#### 3.2.1 Literature exploration using Abstract Sifter

**Fig. 3:**
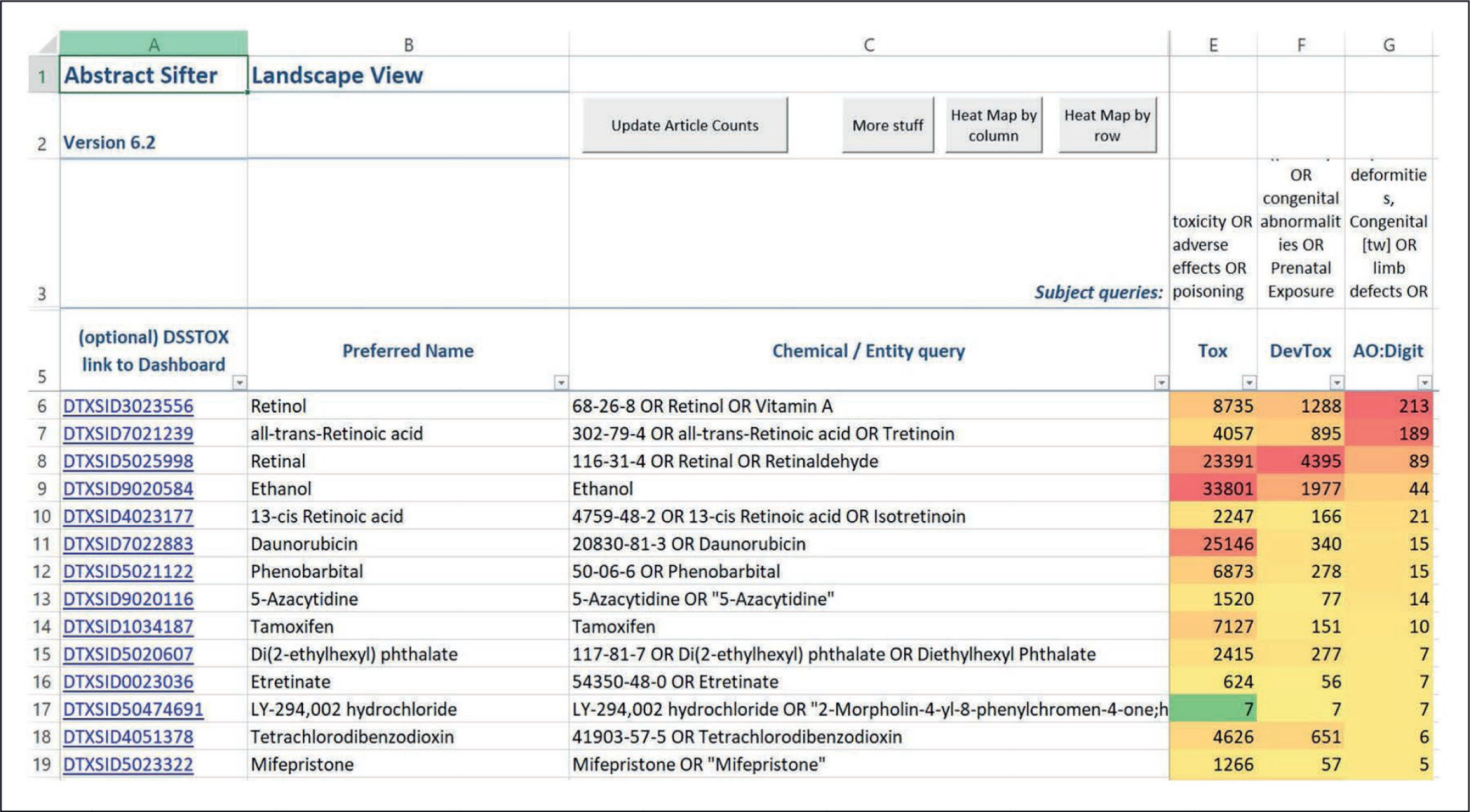
Landscape sheet from the Abstract Sifter Excel application showing literature counts for reference chemicals The numbers represent citation counts returned from PubMed when a query built from the chemical phrase in Column C is appended with “AND” to the subject matter query terms in Column 3. Note: Query results indicate co-occurrence of terms, and publications should be consulted for results.

Approximately 280 putative reference chemicals (referred to as Refchemset) were identified in this work. Results of queries on the Landscape sheet show that, of the chemicals in Refchemset, ethanol, daunorubicin and retinal are associated with the highest number of articles describing toxicity (column E), while retinal has the most citations connecting it to developmental toxicity (Fig. 3). Around 180 (approximately 64%) of the chemicals in Refchemset have some connections to citations about developmental toxicity. When the queries are structured to focus on one developmental adverse outcome associated with retinoid disruption – limb defects – we find approximately 58 chemicals have some supporting literature.

#### 3.2.2 Citral case study

In two studies describing the toxicity of citral, the first to describe the chemical’s effect on embryonic chicks, citral caused malformations in the chick limbs when applied early in undifferentiated tissue (Abramovici et al., 1973, 1980). A study in 1996 used citral to block endogenous ATRA production in the chick embryo and found that citral caused truncated cartilage elements (Tanaka et al., 1996). In a study in zebrafish and mummichog, dosing with excessive ATRA resulted in duplicated fins; citral administration reversed this effect and even caused loss of pectoral fins (Vandersea et al., 1998). In dose-ranging developmental studies in pregnant rats, researchers found that oral doses over 60 mg/kg caused minor skeletal abnormalities and growth retardation (Nogueira et al., 1995). In an inhalation study in pregnant rats, only high maternally toxic doses caused defects, including hypoplastic bones (Gaworski et al., 1992). Citral has significant evidence linking it to the adverse outcome in our limb defect AOP in Figure 2.

Next, we looked for literature linking citral to the other key events in the AOP. RALDH activity comes downstream of several targets discussed here, and it affects levels of ATRA. The key event following RALDH inhibition is a deficiency or down-regulation of ATRA. There is ample literature describing this activity of citral (Connor, 1988); this effect is often the rationale for using the chemical in experiments.

The next step in the AOP describes disrupted FGF signaling. Citral’s activity with respect to FGF during development is described in studies of facial bone development, not limb development. In studies on the developing chick jaw, Shimomura et al. (2015) determined that both ATRA and citral work through the regulation of FGF. In the developing chick face, Song et al. (2004) found that endogenous retinoids and citral act upstream of FGF8 and regulate the programmed cell death and morphogenesis of the face.

The next key event in the AOP revolves around DHAND protein in the zone of polarizing activity (ZPA). A search of PubMed using citral and terms referring to DHAND and ZPA did not produce any citations. The next key event is Homeobox (Hox) gene disruption of retinoid pathway. Two publications describe citral’s inhibitory effect (75% inhibition of *Hoxa1* expression) in a Hox gene expression assay (Chen and Reese, 2013, 2016).

## 4 Discussion

**Fig. 4:**
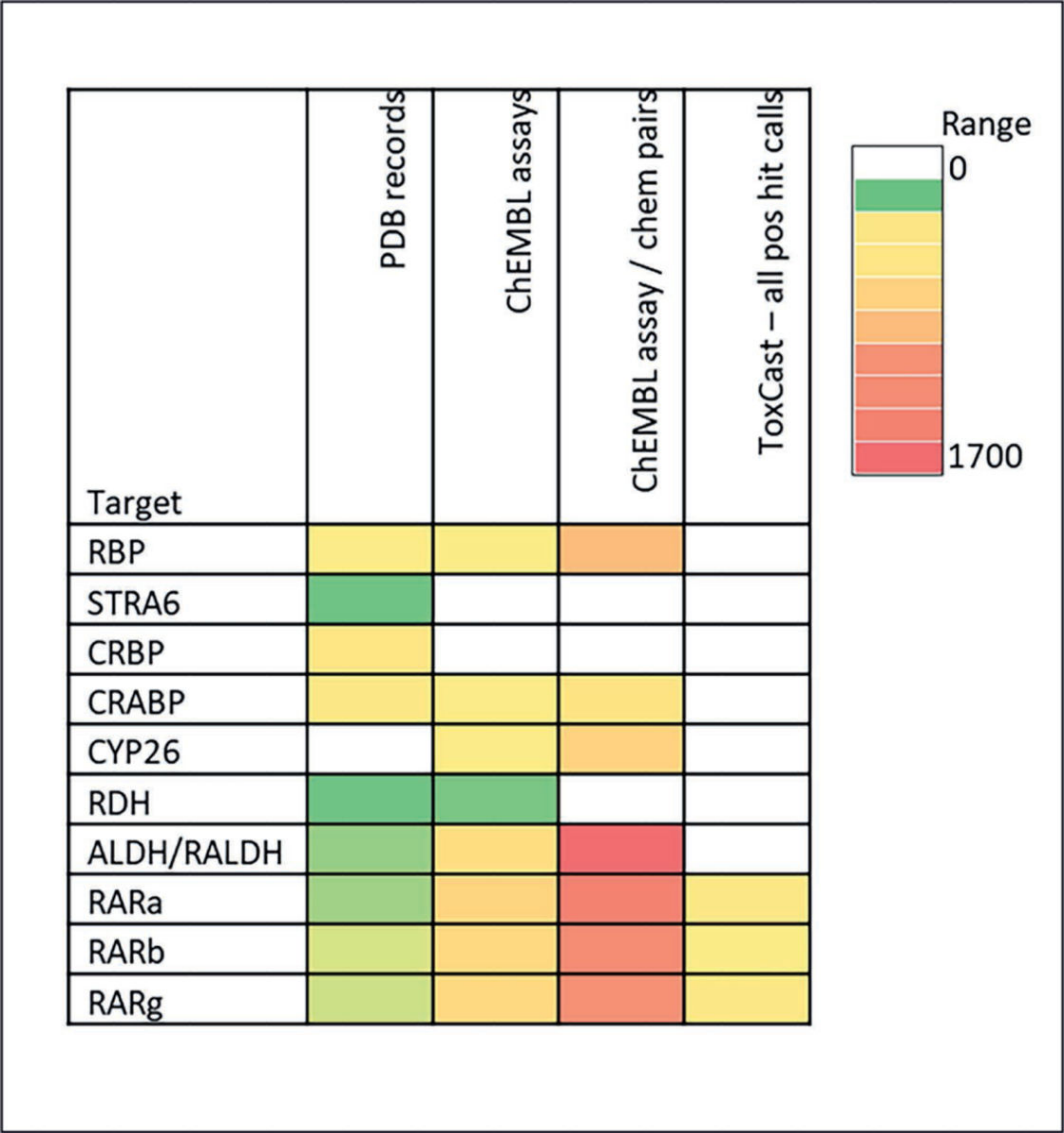
Indicator of data volume for each retinoid pathway target Depth of record counts noted in the top row are indicated by the color bar key.

This work demonstrates that there is a large amount of information regarding the effects of chemicals on gene and protein targets in the retinoid pathway, but the amount of information varies for each target. Figure 4 illustrates this data disparity. A target like retinol binding protein, for example, has many PDB and ChEMBL entries, while STRA8 and CYP26 have very few. Gene and protein targets encompassed in high-throughput testing programs like ToxCast contribute significantly to expanding the target space and the chemical space, but gaps remain. These gaps are significant in the retinoid pathway: the retinoic acid receptors are the only retinoid targets directly tested in ToxCast.

This inconsistency in information volume among the retinoid targets is driven to some extent by the search for therapeutics. Retinoid pathway targets like CRABP receive attention from researchers when they are recognized as potential therapeutic targets, not when they could be involved in an adverse outcome. Binding data available through PDB, while useful for its specificity, is motivated mostly by pharmacological goals, yielding chemicals that are similar to each other in structure. When this is the case, only molecules that achieve some success in the development pipeline have follow-up studies. The information is useful but patchy and can weaken inferences to environmental chemicals. For example, pesticides have been tested against RAR receptors, RALDH, and RDH, but not against RBP and CRABP.

The role of reference chemical metabolism is a challenge to assess consistently. Intact metabolic enzymes may be required to activate a chemical. Conversely, a chemical can be an excellent reference chemical for a cell-free biochemical assay, but not when it is metabolically inactivated in a cell-based assay. Indeed, a chemical can be an excellent reference chemical for a binding assay but have no evidence connecting it to any downstream outcome associated with the binding target.

The project of identifying reference chemicals has a circular aspect. A researcher needs reference chemicals to develop a robust assay but needs assays to identify active chemicals. One approach often employed is to test endogenous retinoids (e.g., retinol, retinal, ATRA, 9-cis-retinoic acid), then exogenous retinoids, and then transition to non-retinoids. This approach can be seen for instance in the study of RBP and CRABP.

High-throughput *in vitro* testing has resulted in millions of data points describing chemical activity at the molecular level, but for most chemicals, any downstream effects in a cell, a tissue, an organ, or an organism are not known and can only be inferred. A chemical like citral has strong evidence linking it to its putative MIE (RALDH inhibition), but despite the long publication history, piecing together evidence that it participates in the key events of provisional AOP for limb defects caused by retinoid pathway disruption is challenged by lack of information, particularly observations of activity at the cellular and tissue level.

As the toxicology community performs fewer and fewer tests on animals, published reports of developmental and reproductive adverse outcomes will slow, even as the number of untested chemicals introduced to the marketplace grows. This data disconnect underscores the importance of developing assays that test the effects of chemicals on complex multi-system tissues such as organ-on-a-chip systems. If these systems can be designed to measure endpoints relevant to important AOPs, more light could be shed on candidate reference chemicals and strengthen the case, not just that they have the molecular initiating event of interest, but that their effects are consistent – or not – with AOPs of interest. When such complex culture assays can be scaled up and run in high-throughput, then candidate reference chemicals can be selected with the support of much stronger lines of evidence.

In this work, we have surveyed the data and literature for ten protein targets in the retinoid pathway and then assembled, discussed, and compiled a set of candidate reference chemicals for each target. Assembling this complex information into one place with links to the data and the literature will facilitate development of new testing programs, new *in vitro* assays, and other new approach methodologies for the retinoid system. These approaches depend on reference chemicals to calibrate the activity thresholds and establish confidence.

## Supplementary Material

Supplement1

Supplement2

Supplement3

## Figures and Tables

**Tab. 1:** Overview of retinoid pathway targets reviewed Table identifiers with “S” are supplemental^1^.

Section	Retinoid target	Long name	Basic function	Data source and entries	Table
3.1	RBP	Retinol binding protein (serum / plasma)	Transports dietary derived and *in situ* synthesized retinol in serum	PDB entries	S1
				ChEMBL assays	S2
3.2	STRA6	stimulated by retinoic acid gene 6 protein	Transports retinol across membrane into cell from bloodstream; receptor for retinol uptake; signaling	PDB entries	S3
3.3	CRBP (CRBP1, CRBP2, CRBP3)	Cellular retinol binding protein	Binds retinol inside the cell	PDB entries	S4
3.4	CRABP (CRABP1, CRABP2)	Cellular retinoic acid binding protein	Binds ATRA; facilitates transfer of ATRA from cytosol to nucleus	PDB entries	S5
				ChEMBL assays	S6
				Fogh et al., 1993	2
				Chaudhuri et al., 1999	2
3.5	CYP26	Retinoic acid 4- hydroxylase OR cytochrome P450 family 26	Degradation of ATRA	ChEMBL assays	S7
				Thatcher et al., 2011; Buttrick, 2013; Foti et al., 2016a,b	3
3.6	RDH	Retinol dehydrogenase	Transforms retinol to retinal through dehydrogenation	ChEMBL assays	S8
3.7	ALDH / RALDH	Aldehyde dehydrogenase / retinal dehydrogenase	Transforms retinal to ATRA through dehydrogenation	PDB entries	S9
				ChEMBL assays	S10
				Koppaka et al., 2012	6
3.8	RARa	Retinoic acid receptor alpha	ATRA receptor	PDB entries	S11
				ChEMBL assays	S12
				ToxCast assay results	S17
3.9	RARb	Retinoic acid receptor beta	ATRA receptor	PDB entries	S13
				ChEMBL assays	S14
				ToxCast assay results	S17
3.10	RARg	Retinoic acid receptor gamma	ATRA receptor	PDB entries	S15
				ChEMBL assays	S16
				ToxCast assay results	S17
3.11	Retinoid pathway	Retinoid pathway		Chen and Reese, 2013; Chen et al., 2016b	7
				ToxCast / Tox21 assay results	S17

**Tab. 2:** Chemicals active in binding assays for CRABP1 and CRABP2 in selected publications

Source	Chemical name
Fogh et al., 1993	CD 367
	All-trans retinoic acid (ATRA)
	TTNPB
	4-oxoretinoic acid
Chaudhuri et al., 1999	All-trans retinoic acid (ATRA)
	Ro13–6307
	Ro12–7310
	Am80 (tamibarotene)
	TTNPB

**Tab. 3:** Chemicals tested for CYP26A1 and/or CYP26B1 inhibition in selected publications For (Foti et al., 2016b), the 17 chemicals that passed the single concentration inhibition screen are listed in addition to results for known CYP26 inhibitors. For all chemicals, consult the publications for measured values, experimental conditions, and cut-offs used.

	Thatcher et al., 2011	Foti et al., 2016a		Foti et al., 2016b		Buttrick, 2013	
Chemical	CYP26A1	CYP26A1	CYP26B1	CYP26A1	CYP26B1	CYP26A1	CYP26B1
17-alpha-ethinyl estradiol				X	X		
AM580		X	X			X	X
AM80 (tamibarotene)		X	X	X	X	X	X
Benzbromarone				X	X		
Bexarotene				X	X	X	X
BMS753		X	X			X	X
BMS961		X	X			X	X
Candesartan				X	X		
Candesartan cilexetil				X	X		
CD1530	X						
CD437				X	X		
Clotrimazole				X	X		
CS5	X						
EC23				X	X	X	X
Fluconazole				X	X		
Itraconazole				X	X		
Ketoconazole	X			X	X	X	X
L-165,041	X						
Liarozole	X			X	X	X	X
MM11253				X	X	X	X
Mometasone				X	X		
Montelukast				X	X		
Pioglitazone	X			X	X		
Quercetin				X	X		
R115866 (talarozole)	X			X	X	X	X
R116010	X					X	X
Raloxifene				X	X		
Repaglinide				X	X		
Ritonavir				X	X		
Rosiglitazone	X			X	X		
SR11237				X	X	X	X
Tamoxifen				X	X		
Tazarotene						X	X
Tazarotenic acid (Agn 190299)		X	X			X	X
TTNPB	X	X	X			X	X
Zafirlukast				X	X		

**Tab. 4:** Major RDH genes from Napoli (2012)

Mouse	Rat	Human	Note
Rdh1	Rdh7 and Rdh2 (originally RodhI and RodhII)	Rdh16 (originally Rodh4, RDH-E)	Sometimes referred to as ADH Class IV
Rdh10	Rdh10	Rdh10	
Dhrs9	Dhrs9 (originally eRolDH2)	Dhrs9 (originally retSdr8, RDHL, Rdh-TBE, RoDH-E2, 3α-HSD)	

**Tab. 5:** Subset of aldehyde dehydrogenases (adapted from Koppaka et al., 2012)

Gene	Preferred substrate
ALDH1A1	Retinal
ALDH1A2	Retinal
ALDH1A3	Retinal
ALDH1B1	Retinal and acetaldehyde
ALDH2	Acetaldehyde
ALDH8A1	Retinal

**Tab. 6:** Known inhibitors of aldehyde dehydrogenases (adapted from Koppaka et al., 2012)

Chemical inhibitor
Ampal
Benomyl
Citral
Chloral hydrate
Chlorpromamide analogs
Coprine
Cyanamide
Daidzin
CVT-10216
DEAB
Disulfiram
Gossypol
Kynurenine
Molinate
Nitroglycerin
Pargyline

**Tab. 7:** Summary of results from published retinoid pathway assays The publications should be consulted for potency, measurements, and assay details.

Chen et al., 2016b		Chen and Reese, 2013
Agonists	Antagonists	Active
1,10-Phenanthroline monohydrate	5-Azacytidine	Ethanol
10058-F4	Amoxapine	4-Methylpyrazole
13-cis-retinoic acid	Amsacrine hydrochloride	Geraniol
4-Aminoazobenzene	Auranofin	3,7-Dimethyloctan-1-ol
AC-55649	Bay 11–7085	Citronellol
BF-170 hydrochloride	BAY 61–3606 hydrochloride hydrate	Citral
DFB	Brefeldin A from Penicillium brefeldianum	Citronellal
GW9662	Camptothecin	Bisdiamine
K114	CGP-74514A hydrochloride	4-(Diethylamino)benzaldehyde (DEAB)
Kenpaullone	CGP-7930	Nitrofen
Niclosamide	Desipramine hydrochloride	Metam-sodium
PD 98,059	Dilazep hydrochloride	Thiram
Retinoic acid (ATRA)	D-ribofuranosylbenzimidazole	4-Nonylphenol
Retinoic acid p-hydroxyanilide	Emetine dihydrochloride hydrate	Diethylstilbestrol
Rhodblock 6	H-8 dihydrochloride	Bisphenol A
Rutaecarpine	Idarubicin hydrochloride	Genistein
SB 204741	LY-294,002 hydrochloride	Dibutyl phthalate
SB 206553 hydrochloride	Mifepristone	Dipentyl phthalate
SB-366791	Mitoxantrone	Di(2-ethylhexyl) phthalate
SCH 58261	MNS	
SIB 1757	Parthenolide	
SIB 1893	PD-166285 hydrate	
SU 4312	Spironolactone	
TTNPB	Stattic	
Tyrphostin AG 494	Topotecan hydrochloride hydrate	

## Data Availability

The ToxCast data is available from the EPA Chemicals Dashboard^6^.

## Footnotes

1 doi:10.14573/altex.2202231s1

2
https://comptox.epa.gov/dashboard

3 doi:10.14573/altex.2202231s2

4
https://comptox.epa.gov/dashboard/downloads

5 doi:10.14573/altex.2202231s3

## Abbreviations

ADH: alcohol dehydrogenase
ALDH: aldehyde dehydrogenase
AOP: adverse outcome pathway
ATRA: all-trans-retinoic acid (tretinoin for pharmaceutical use)
CRABP: cellular retinoic acid binding protein
CYP26: cytochrome P450 family 26
DART: developmental and reproductive toxicology
DHAND: heart and neural crest derivatives protein
DR5: direct repeats of 5 nucleotides for RAR/RXR transactivation
DRP: detailed review paper
FGF: fibroblast growth factor
Hoxa1: homeobox A1
NAM: new approach methods
OECD: Organization of Economic Cooperation and Development
RALDH: retinal dehydrogenase
RAR: retinoic acid receptor
RARE: retinoic acid response element
RBP: retinol binding protein (plasma or serum)
RDH: retinol dehydrogenase
RXR: retinoid X receptor
SDR: short-chain dehydrogenase/reductase
STRA6: stimulated by retinoic acid 6
ZPA: zone of polarizing activity

## References

[R1] Abramovici A, Liban E, Ben-David E (1973). The ultrastructure of striated muscle in malformed chick limb induced by citral. Virchows Arch B Cell Pathol 14, 127–134. doi:10.1007/bf02889182 4203885

[R2] Abramovici A, Rachmuth-Forschmidt P, Liban E (1980). Experimental limb dysmorphogenesis as a model of chemical injury response in undifferentiated embryonic tissues: A light and electron microscopical study. J Pathol 131, 289–308. doi:10.1002/path.1711310402 7431150

[R3] Agarwal C, Chandraratna RA, Johnson AT (1996). AGN193109 is a highly effective antagonist of retinoid action in human ectocervical epithelial cells. J Biol Chem 271, 12209–12212. doi:10.1074/jbc.271.21.12209 8647816

[R4] Allali-Hassani A, Peralba JM, Martras S (1998). Retinoids, omega-hydroxyfatty acids and cytotoxic aldehydes as physiological substrates, and H2-receptor antagonists as pharmacological inhibitors, of human class IV alcohol dehydrogenase. FEBS Lett 426, 362–366. doi:10.1016/s0014-5793(98)00374-3 9600267

[R5] Allen EM, Anderson DG, Florang VR (2010). Relative inhibitory potency of molinate and metabolites with aldehyde dehydrogenase 2: Implications for the mechanism of enzyme inhibition. Chem Res Toxicol 23, 1843–1850. doi:10.1021/tx100317q 20954713 PMC2989800

[R6] Anonymous (2004). Tamibarotene: AM 80, retinobenzoic acid, tamibaro. Drugs R D 5, 359–362. doi:10.2165/00126839-200405060-00010 15563242

[R7] Attene-Ramos MS, Miller N, Huang R (2013). TheTox21 robotic platform for the assessment of environmental chemicals – From vision to reality. Drug Discov Today 18, 716–723. doi:10.1016/j.drudis.2013.05.015 23732176 PMC3771082

[R8] Baker NC and Hemminger BM (2010). Mining connections between chemicals, proteins, and diseases extracted from Medline annotations. J Biomed Inform 43, 510–519. doi:10.1016/j.jbi.2010.03.008 20348023 PMC2902698

[R9] Baker N, Knudsen T and Williams A (2017). Abstract Sifter: A comprehensive front-end system to PubMed. F1000Res 6, 2164. doi:10.12688/f1000research.12865.1 PMC5801564 29479422

[R10] Balmer JE and Blomhoff R (2002). Gene expression regulation by retinoic acid. J Lipid Res 43, 1773–1808. doi:10.1194/jlr.r100015-jlr200 12401878

[R11] Bell RG and Smith HW (1949). Preliminary report on clinical trials of antabuse. Can Med Assoc J 60, 286–288. PMC1591479 18110807

[R12] Berman H, Henrick K and Nakamura H (2003). Announcing the worldwide Protein Data Bank. Nat Struct Biol 10, 980. doi:10.1038/nsb1203-980 14634627

[R13] Berry DC, O’Byrne SM, Vreeland AC (2012). Cross talk between signaling and vitamin a transport by the retinol-binding protein receptor STRA6. Mol Cell Biol 32, 3164–3175. doi:10.1128/mcb.00505-12 22665496 PMC3434520

[R14] Blume-Peytavi U, Fowler J, Kemeny L (2020). Long-term safety and efficacy of trifarotene 50 μg/g cream, a first-in-class RAR-γ selective topical retinoid, in patients with moderate facial and truncal acne. J Eur Acad Dermatol Venereol 34, 166–173. doi:10.1111/jdv.15794 31306527 PMC7004112

[R15] Boerman MH and Napoli JL (1995). Characterization of a microsomal retinol dehydrogenase: A short-chain alcohol dehydrogenase with integral and peripheral membrane forms that interacts with holo-CRBP (type I). Biochemistry 34, 7027–7037. doi:10.1021/bi00021a014 7766612

[R16] Breen CJ, Martin DS, Ma H (2015). Production of functional human vitamin A transporter/RBP receptor (STRA6) for structure determination. PLoS One 10, e0122293. doi:10.1371/journal.pone.0122293 25816144 PMC4376794

[R17] Brtko J and Dvorak Z (2020). Natural and synthetic retinoid X receptor ligands and their role in selected nuclear receptor action. Biochimie 179, 157–168. doi:10.1016/j.biochi.2020.09.027 33011201

[R18] Bruno RD and Njar VC (2007). Targeting cytochrome P450 enzymes: A new approach in anti-cancer drug development. Bioorg Med Chem 15, 5047–5060. doi:10.1016/j.bmc.2007.05.046 17544277 PMC1958998

[R19] Buttrick BR (2013). Characterization of selective and potent inhibitors of the human retinoic acid hydroxylases CYP26A1 and CYP26B1. MSc thesis, University of Washington. https://bit.ly/3X23tOZ

[R20] Cao S, Wang G, Ge F (2019). Gossypol inhibits 5α-reductase 1 and 3α-hydroxysteroid dehydrogenase: Its possible use for the treatment of prostate cancer. Fitoterapia 133, 102–108. doi:10.1016/j.fitote.2018.12.024 30605780

[R21] Chaudhuri BN, Kleywegt GJ, Broutin-L’Hermite I (1999). Structures of cellular retinoic acid binding proteins I and II in complex with synthetic retinoids. Acta Crystallogr D Biol Crystallogr 55, 1850–1857. doi:10.1107/s0907444999011026 10531482

[R22] Chen CH, Lin KD, Ke LY (2019). O-GlcNAcylation disrupts STRA6-retinol signals in kidneys of diabetes. Biochim Biophys Acta Gen Subj 1863, 1059–1069. doi:10.1016/j.bbagen.2019.03.014 30905621

[R23] Chen H, Chidboy MA and Robinson JF (2020). Retinoids and developmental neurotoxicity: Utilizing toxicogenomics to enhance adverse outcome pathways and testing strategies. Reprod Toxicol 96, 102–113. doi:10.1016/j.reprotox.2020.06.007 32544423 PMC7736340

[R24] Chen R, Chen F, Han J (2001). Effects of selective rar or/ and rxr retinoids on the proliferation and differentiation of nb4 cells and their mechanisms [Article in Chinese]. Zhonghua Xue Ye Xue Za Zhi 22, 256–259. 11877083

[R25] Chen WS, Bohlken DP and Plapp BV (1981). Inactivation of liver alcohol dehydrogenases and inhibition of ethanol metabolism by ambivalent active-site-directed reagents. J Med Chem 24, 190–193. doi:10.1021/jm00134a012 7009869

[R26] Chen Y and Reese DH (2013). A screen for disruptors of the retinol (vitamin A) signaling pathway. Birth Defects Res B Dev Reprod Toxicol 98, 276–282. doi:10.1002/bdrb.21062 23696197

[R27] Chen Y and Reese DH (2016). Disruption of retinol (vitamin A) signaling by phthalate esters: SAR and mechanism studies. PLoS One 11, e0161167. doi:10.1371/journal.pone.0161167 PMC4988654 27532513

[R28] Chen Y, Clarke OB, Kim J (2016a). Structure of the stra6 receptor for retinol uptake. Science 353, aad8266. doi:10.1126/science.aad8266 PMC5114850 27563101

[R29] Chen Y, Sakamuru S, Huang R (2016b). Identification of compounds that modulate retinol signaling using a cell-based qHTS assay. Toxicol In Vitro 32, 287–296. doi:10.1016/j.tiv.2016.01.011 26820057 PMC4779714

[R30] Chen Y, Zhu JY, Hong KH (2018). Structural basis of ALDH1A2 inhibition by irreversible and reversible small molecule inhibitors. ACS Chem Biol 13, 582–590. doi:10.1021/acschembio.7b00685 29240402 PMC6089219

[R31] Cioffi CL, Dobri N, Freeman EE (2014). Design, synthesis, and evaluation of nonretinoid retinol binding protein 4 antagonists for the potential treatment of atrophic age-related macular degeneration and Stargardt disease. J Med Chem 57, 7731–7757. doi:10.1021/jm5010013 25210858 PMC4174998

[R32] Collins MD, Eckhoff C, Chahoud I (1992). 4-Methylpyrazole partially ameliorated the teratogenicity of retinol and reduced the metabolic formation of all-trans-retinoic acid in the mouse. Arch Toxicol 66, 652–659. doi:10.1007/bf01981505 1482289

[R33] Connor MJ and Smit MH (1987). Terminal-group oxidation of retinol by mouse epidermis. Inhibition in vitro and in vivo. Biochem J 244, 489–492. doi:10.1042/bj2440489 3663136 PMC1148017

[R34] Connor MJ (1988). Oxidation of retinol to retinoic acid as a requirement for biological activity in mouse epidermis. Cancer Res 48, 7038–7040. 3191479

[R35] Damdimopoulou P, Chiang C and Flaws JA (2019). Retinoic acid signaling in ovarian folliculogenesis and steroidogenesis. Reprod Toxicol 87, 32–41. doi:10.1016/j.reprotox.2019.04.007 31059772 PMC6613987

[R36] Donovan M, Olofsson B, Gustafson AL (1995). The cellular retinoic acid binding proteins. J Steroid Biochem Mol Biol 53, 459–465. doi:10.1016/0960-0760(95)00092-e 7626495

[R37] Duester G (1991). A hypothetical mechanism for fetal alcohol syndrome involving ethanol inhibition of retinoic acid synthesis at the alcohol dehydrogenase step. Alcohol Clin Exp Res 15, 568–572. doi:10.1111/j.1530-0277.1991.tb00562.x 1877746

[R38] Duester G (2008). Retinoic acid synthesis and signaling during early organogenesis. Cell 134, 921–931. doi:10.1016/j.cell.2008.09.002 18805086 PMC2632951

[R39] Elmazar MM, Reichert U, Shroot B (1996). Pattern of retinoid-induced teratogenic effects: Possible relationship with relative selectivity for nuclear retinoid receptors RAR alpha, RAR beta, and RAR gamma. Teratology 53, 158–167. doi:10.1002/(sici)1096-9926(199603)53:3<158::aid-tera3>3.0.co;2-0 8761883

[R40] Favorskaya I, Kainov Y, Chemeris G (2014). Expression and clinical significance of CRABP1 and CRABP2 in non-small cell lung cancer. Tumour Biol 35, 10295–10300. doi:10.1007/s13277-014-2348-4 25034531

[R41] Filer DL, Kothiya P, Setzer RW (2017). tcpl: The toxcast pipeline for high-throughput screening data. Bioinformatics 33, 618–620. doi:10.1093/bioinformatics/btw680 27797781

[R42] Finulli M and Magistretti M (1961). [Antabuse-like toxic manifestations in workmen employed in the manufacture of a synthetic anticryptogamic: T.M.T.D. (tetramethylthiuram disulfide) [Article in Italian]. Med Lav 52, 132–137. 13699710

[R43] Fogh K, Voorhees JJ and Astrom A (1993). Expression, purification, and binding properties of human cellular retinoic acid-binding protein type I and type II. Arch Biochem Biophys 300, 751–755. doi:10.1006/abbi.1993.1104 8382035

[R44] Foti RS, Isoherranen N, Zelter A (2016a). Identification of tazarotenic acid as the first xenobiotic substrate of human retinoic acid hydroxylase CYP26A1 and CYP26B1. J Pharmacol Exp Ther 357, 281–292. doi:10.1124/jpet.116.232637 26937021 PMC4851321

[R45] Foti RS, Diaz P and Douguet D (2016b). Comparison of the ligand binding site of CYP2C8 with CYP26A1 and CYP26B1: A structural basis for the identification of new inhibitors of the retinoic acid hydroxylases. J Enzyme Inhib Med Chem 31, 148161. doi:10.1080/14756366.2016.1193734 PMC6628712 27424662

[R46] Galdones E and Hales BF (2008). Retinoic acid receptor gamma-induced misregulation of chondrogenesis in the murine limb bud in vitro. Toxicol Sci 106, 223–232. doi:10.1093/toxsci/kfn169 18703560

[R47] Galli A, Pinaire J, Fischer M (2001). The transcriptional and DNA binding activity of peroxisome proliferator-activated receptor alpha is inhibited by ethanol metabolism. A novel mechanism for the development of ethanol-induced fatty liver. J Biol Chem 276, 68–75. doi:10.1074/jbc.m008791200 11022051

[R48] Garnier R, Chataigner D and Efthymiou ML (1992). Skin and eye burns, painful abdomen syndrome, antabuse effect, and cytolytic hepatitis in workers exposed to dimethylformamide [Article in French]. J Toxicol Clin Exp 12, 227–237. 1295974

[R49] Gaulton A, Hersey A, Nowotka M (2017). The ChEMBL database in 2017. Nucleic Acids Res 45, D945–D954. doi:10.1093/nar/gkw1074 27899562 PMC5210557

[R50] Gaworski CL, Vollmuth TA, York RG (1992). Developmental toxicity evaluation of inhaled citral in Sprague-Dawley rats. Food Chem Toxicol 30, 269–275. doi:10.1016/0278-6915(92)90003-4 1628861

[R51] Germain P, Gaudon C, Pogenberg V (2009). Differential action on coregulator interaction defines inverse retinoid agonists and neutral antagonists. Chem Biol 16, 479–489. doi:10.1016/j.chembiol.2009.03.008 19477412

[R52] Grignard E, Hakansson H and Munn S (2020). Regulatory needs and activities to address the retinoid system in the context of endocrine disruption: The European viewpoint. Reprod Toxicol 93, 250–258. doi:10.1016/j.reprotox.2020.03.002 32171711 PMC7322530

[R53] Helvig C, Taimi M, Cameron D (2011). Functional properties and substrate characterization of human CYP26A1, CYP26B1, and CYP26C1 expressed by recombinant baculovirus in insect cells. J Pharmacol Toxicol Methods 64, 258–263. doi:10.1016/j.vascn.2011.08.005 21906690

[R54] Hu W, Verschraegen CF, Wu WG (2002). Activity of ALRT 1550, a new retinoid, with interferon-gamma on ovarian cancer cell lines. Int J Gynecol Cancer 12, 202–207. doi:10.1046/j.1525-1438.2002.01084.x 11975681

[R55] Huddle BC, Grimley E, Buchman CD (2018). Structure-based optimization of a novel class of aldehyde dehydrogenase 1A (ALDH1A) subfamily-selective inhibitors as potential adjuncts to ovarian cancer chemotherapy. J Med Chem 61, 8754–8773. doi:10.1021/acs.jmedchem.8b00930.s002 30221940 PMC6477540

[R56] Hussain RM, Gregori NZ, Ciulla TA (2018). Pharmacotherapy of retinal disease with visual cycle modulators. Expert Opin Pharmacother 19, 471–481. doi:10.1080/14656566.2018. 1448060 29542350

[R57] Jacobsen E and Larsen V (1949). Site of the formation of acetaldehyde after ingestion of antabuse (tetraethylthiuramdisulphide) and alcohol. Acta Pharmacol Toxicol (Copenh) 5, 285–291. doi:10.1111/j.1600-0773.1949.tb03393.x 15393457

[R58] Jin N, Zhu X, Cheng F (2018). Disulfiram/copper targets stem cell-like ALDH^+^ population of multiple myeloma by inhibition of ALDH1A1 and hedgehog pathway. J Cell Biochem 119, 6882–6893. doi:10.1002/jcb.26885 29665144

[R59] Johnson AT, Klein ES, Gillett SJ (1995). Synthesis and characterization of a highly potent and effective antagonist of retinoic acid receptors. J Med Chem 38, 4764–4767. doi:10.1021/jm00024a003 7490725

[R60] Ju J, Wang N, Wang J (2018). 4-Amino-2-trifluoromethylphenyl retinate inhibits proliferation, invasion, and migration of breast cancer cells by independently regulating CRABP2 and FABP5. Drug Des Devel Ther 12, 997–1008. doi:10.2147/dddt.s151029 PMC5927060 29731607

[R61] Judson RS, Houck KA, Kavlock RJ (2010). In vitro screening of environmental chemicals for targeted testing prioritization: The ToxCast project. Environ Health Perspect 118, 485–492. doi:10.1289/ehp.0901392 20368123 PMC2854724

[R62] Judson RS, Thomas RS, Baker N (2019). Workflow for defining reference chemicals for assessing performance of in vitro assays. ALTEX 36, 261–276. doi:10.14573/altex.1809281 30570668 PMC6784312

[R63] Kagechika H, Kawachi E, Hashimoto Y (1988). Retinobenzoic acids. 1. Structure-activity relationships of aromatic amides with retinoidal activity. J Med Chem 31, 2182–2192. doi:10.1021/jm00119a021 3184125

[R64] Kaminskaia ZA (1949). Primenenie citralia pri glaukome [Application of citral in glaucoma]. Sov Med 13, 37. 18142086

[R65] Kar AB, Jehan Q, Kamboj VP (1966). Effect of N,N’bis(dichloroacetyl)-1,8-octamethylenediamine on the chemical composition of the rat seminiferous tubules. Int J Fertil 11, 291–296. 6007836

[R66] Kast RE and Belda-Iniesta C (2009). Suppressing glioblastoma stem cell function by aldehyde dehydrogenase inhibition with chloramphenicol or disulfiram as a new treatment adjunct: An hypothesis. Curr Stem Cell Res Ther 4, 314–317. doi:10.2174/157488809789649241 19500061

[R67] Kawaguchi R and Sun H (2010). Techniques to study specific cell-surface receptor-mediated cellular vitamin A uptake. Methods Mol Biol 652, 341–361. doi:10.1007/978-1-60327-325-1_20 20552439 PMC3907174

[R68] Kelly M and von Lintig J (2015). STRA6: Role in cellular retinol uptake and efflux. Hepatobiliary Surg Nutr 4, 229–242. doi:10.3978/j.issn.2304-3881.2015.01.12 26312242 PMC4526761

[R69] Kikonyogo A, Abriola DP, Dryjanski M (1999). Mechanism of inhibition of aldehyde dehydrogenase by citral, a retinoid antagonist. Eur J Biochem 262, 704–712. doi:10.1046/j.1432-1327.1999.00415.x 10411631

[R70] Kim S, Chen J, Cheng T (2019). PubChem 2019 update: Improved access to chemical data. Nucleic Acids Res 47, D1102–D1109. doi:10.1093/nar/gky1033 30371825 PMC6324075

[R71] Kim YJ, Kim JY, Lee N (2017). Disulfiram suppresses cancer stem-like properties and STAT3 signaling in triple-negative breast cancer cells. Biochem Biophys Res Commun 486, 1069–1076. doi:10.1016/j.bbrc.2017.03.164 28373070

[R72] Klaholz BP, Renaud JP, Mitschler A (1998). Conformational adaptation of agonists to the human nuclear receptor RAR gamma. Nat Struct Biol 5, 199–202. doi:10.1038/nsb0398-199 9501913

[R73] Knudsen TB, Pierro JD and Baker NC (2021). Retinoid signaling in skeletal development: Scoping the system for predictive toxicology. Reprod Toxicol 99, 109–130. doi:10.1016/j.reprotox.2020.10.014 33202217 PMC11451096

[R74] Kochhar DM (1973). Limb development in mouse embryos. I. Analysis of teratogenic effects of retinoic acid. Teratology 7, 289–295. doi:10.1002/tera.1420070310 4713875

[R75] Kochhar DM, Jiang H, Penner JD (1998). The use of a retinoid receptor antagonist in a new model to study vitamin A-dependent developmental events. Int J Dev Biol 42, 601–608. 9694631

[R76] Koppaka V, Thompson DC, Chen Y (2012). Aldehyde dehydrogenase inhibitors: A comprehensive review of the pharmacology, mechanism of action, substrate specificity, and clinical application. Pharmacol Rev 64, 520–539. doi:10.1124/pr.111.005538 22544865 PMC3400832

[R77] Kumar S, Sandell LL, Trainor PA (2012). Alcohol and aldehyde dehydrogenases: Retinoid metabolic effects in mouse knockout models. Biochimica et Biophysica Acta 1821, 198–205. doi:10.1016/j.bbalip.2011.04.004 21515404 PMC3161159

[R78] Lampen A, Meyer S, Arnhold T (2000). Metabolism of vitamin A and its active metabolite all-trans-retinoic acid in small intestinal enterocytes. J Pharmacol Exp Ther 295, 979–985. 11082432

[R79] Lee GS, Kochhar DM and Collins MD (2004). Retinoid-induced limb malformations. Curr Pharm Des 10, 2657–2699. doi:10.2174/1381612043383728 15320736

[R80] Lee SA, Yang KJZ, Brun PJ (2020). Retinol-binding protein 2 (RBP2) binds monoacylglycerols and modulates gut endocrine signaling and body weight. Sci Adv 6, eaay8937. doi:10.1126/sciadv.aay8937 PMC7065888 32195347

[R81] Lemaire G, Balaguer P, Michel S (2005). Activation of retinoic acid receptor-dependent transcription by organochlorine pesticides. Toxicol Appl Pharmacol 202, 38–49. doi:10.1016/j.taap.2004.06.004 15589975

[R82] Li D, Wang M, Cheng S (2017). CYP1A1 based on metabolism of xenobiotics by cytochrome P450 regulates chicken male germ cell differentiation. In Vitro Cell Dev Biol Anim 53, 293–303. doi:10.1007/s11626-016-0108-z 28364347

[R83] Li Z, Yao SJ, Alini M (2011). The role of retinoic acid receptor inhibitor LE135 on the osteochondral differentiation of human bone marrow mesenchymal stem cells. J Cell Biochem 112, 963–970. doi:10.1002/jcb.23013 21308729

[R84] Lim W, Ham J, Park S (2019). Gossypol induces disruption of spermatogenesis and steroidogenesis in male mice. J Agric Food Chem 67, 2075–2085. doi:10.1021/acs.jafc.8b06946 30678458

[R85] Liu P, Brown S, Goktug T (2012). Cytotoxic effect of disulfiram/copper on human glioblastoma cell lines and ALDH-positive cancer-stem-like cells. Br J Cancer 107, 1488–1497. doi:10.1038/bjc.2012.442 23033007 PMC3493777

[R86] Liu RZ, Garcia E, Glubrecht DD (2015). CRABP1 is associated with a poor prognosis in breast cancer: Adding to the complexity of breast cancer cell response to retinoic acid. Mol Cancer 14, 129. doi:10.1186/s12943-015-0380-7 26142905 PMC4491424

[R87] Lu C, Li X, Ren Y (2021). Disulfiram: A novel repurposed drug for cancer therapy. Cancer Chemother Pharmacol 87, 159–172. doi:10.1007/s00280-020-04216-8 33426580

[R88] Maguire M, Larsen MC, Vezina CM (2020). Cyp1b1 directs Srebp-mediated cholesterol and retinoid synthesis in perinatal liver; association with retinoic acid activity during fetal development. PLoS One 15, e0228436. doi:10.1371/journal.pone.0228436 32027669 PMC7004353

[R89] Mao B, Wu C, Zheng W (2018). Methoxychlor and its metabolite HPTE inhibit rat neurosteroidogenic 3α-hydroxysteroid dehydrogenase and retinol dehydrogenase 2. Neurosci Lett 684, 169–174. doi:10.1016/j.neulet.2018.08.008 30107201

[R90] Mark M, Ghyselinck NB and Chambon P (2009). Function of retinoic acid receptors during embryonic development. Nucl Recept Signal 7, e002. doi:10.1621/nrs.07002 19381305 PMC2670431

[R91] Metzler MA and Sandell LL (2016). Enzymatic metabolism of vitamin A in developing vertebrate embryos. Nutrients 8, 812. doi:10.3390/nu8120812 27983671 PMC5188467

[R92] Mey J, Babiuk RP, Clugston R (2003). Retinal dehydrogenase-2 is inhibited by compounds that induce congenital diaphragmatic hernias in rodents. Am J Pathol 162, 673–679. doi:10.1016/s0002-9440(10)63861-8 12547725 PMC1851155

[R93] Molotkov A and Duester G (2002). Retinol/ethanol drug interaction during acute alcohol intoxication in mice involves inhibition of retinol metabolism to retinoic acid by alcohol dehydrogenase. J Biol Chem 277, 22553–22557. doi:10.1074/jbc.m201603200 11960985

[R94] Momma K, Ando M and Takao A (1990). Fetal cardiac morphology of tetralogy of Fallot with absent pulmonary valve in the rat. Circulation 82, 1343–1351. doi:10.1161/01.cir.82.4.1343 2401068

[R95] Moretti A, Li J, Donini S (2016). Crystal structure of human aldehyde dehydrogenase 1A3 complexed with NAD^+^ and retinoic acid. Sci Rep 6, 35710. doi:10.1038/srep35710 27759097 PMC5069622

[R96] Morgan CA and Hurley TD (2015). Characterization of two distinct structural classes of selective aldehyde dehydrogenase 1A1 inhibitors. J Med Chem 58, 1964–1975. doi:10.1021/jm501900s 25634381 PMC4344389

[R97] Morgan CA, Parajuli B, Buchman CD (2015). N,Ndiethylaminobenzaldehyde (DEAB) as a substrate and mechanism-based inhibitor for human ALDH isoenzymes. Chem Biol Interact 234, 18–28. doi:10.1016/j.cbi.2014.12.008 25512087 PMC4414715

[R98] Mujawar I, Sabatino M, Ray Mitchell S (2014). A 12year comparison of students’ perspectives on diversity at a Jesuit medical school. Med Educ Online 19, 23401. doi:10.3402/meo.v19.23401 24581334 PMC3938797

[R99] Napoli JL (2012). Physiological insights into all-trans-retinoic acid biosynthesis. Biochim Biophys Acta 1821, 152–167. doi:10.1016/j.bbalip.2011.05.004 21621639 PMC3179567

[R100] Napoli JL (2016). Functions of intracellular retinoid binding-proteins. Subcell Biochem 81, 21–76. doi:10.1007/978-94-024-0945-1_2 27830500 PMC5493979

[R101] Napoli JL (2017). Cellular retinoid binding-proteins, CRBP, CRABP, FABP5: Effects on retinoid metabolism, function and related diseases. Pharmacol Ther 173, 19–33. doi:10.1016/j.pharmthera.2017.01.004 28132904 PMC5408321

[R102] Napoli JL (2020). Post-natal all-trans-retinoic acid biosynthesis. Methods Enzymol 637, 27–54. doi:10.1016/bs.mie.2020.02.003 32359649 PMC7357352

[R103] Niederreither K, Vermot J, Schuhbaur B (2002). Embryonic retinoic acid synthesis is required for forelimb growth and anteroposterior patterning in the mouse. Development 129, 3563–3574. doi:10.1242/dev.129.15.3563 12117807

[R104] Nogueira AC, Carvalho RR, Souza CA (1995). Study on the embryofeto-toxicity of citral in the rat. Toxicology 96, 105–113. doi:10.1016/0300-483x(94)02915-h 7886681

[R105] Noy N (2016). Vitamin A transport and cell signaling by the retinol-binding protein receptor STRA6. Subcell Biochem 81, 77–93. doi:10.1007/978-94-024-0945-1_3 27830501

[R106] OECD (2014). Guidance Document on Standardised Test Guidelines for Evaluating Chemicals for Endocrine Disruption. OECD Publishing, Paris. doi:10.1787/9789264221413-en

[R107] OECD (2021). Detailed Review Paper on the Retinoid System. Series on Testing and Assessment, No. 343. OECD Publishing, Paris. https://www.oecd.org/officialdocuments/publicdisplaydocumentpdf/?cote=ENV-CBC-MONO(2021)20%20&doclanguage=en

[R108] Oster G, Salgo MP and Taleporos P (1974). Embryocidal action of a bis(dichloroacetyl)-diamine: An oral abortifacient for rats. Am J Obstet Gynecol 119, 583–588. doi:10.1016/0002-9378(74)90117-3 4857790

[R109] Pasutto F, Sticht H, Hammersen G (2007). Mutations in STRA6 cause a broad spectrum of malformations including anophthalmia, congenital heart defects, diaphragmatic hernia, alveolar capillary dysplasia, lung hypoplasia, and mental retardation. Am J Hum Genet 80, 550–560. doi:10.1086/512203 17273977 PMC1821097

[R110] Persson B, Kallberg Y, Bray JE (2009). The SDR (short-chain dehydrogenase/reductase and related enzymes) nomenclature initiative. Chem Biol Interact 178, 94–98. doi:10.1016/j.cbi.2008.10.040 19027726 PMC2896744

[R111] Pignatello MA, Kauffman FC and Levin AA (1997). Multiple factors contribute to the toxicity of the aromatic retinoid, TTNPB (Ro 13–7410): Binding affinities and disposition. Toxicol Appl Pharmacol 142, 319–327. doi:10.1006/taap.1996.8047 9070355

[R112] Plouvier B, Lemoine X, De Coninck P (1982). Antabuse effect during the administration of a topical drug based on monosulfiram [Article in French]. Nouv Presse Med 11, 3209. 7177842

[R113] Quattrini L, Gelardi ELM, Coviello V (2020). Imidazo[1,2-a]pyridine derivatives as aldehyde dehydrogenase inhibitors: Novel chemotypes to target glioblastoma stem cells. J Med Chem 63, 4603–4616. doi:10.1021/acs.jmedchem.9b01910 32223240

[R114] Quistad GB, Sparks SE and Casida JE (1994). Aldehyde dehydrogenase of mice inhibited by thiocarbamate herbicides. Life Sci 55, 1537–1544. doi:10.1016/0024-3205(94)00314-9 7968224

[R115] Racz B, Varadi A, Kong J (2018). A non-retinoid antagonist of retinol-binding protein 4 rescues phenotype in a model of Stargardt disease without inhibiting the visual cycle. J Biol Chem 293, 11574–11588. doi:10.1074/jbc.ra118.002062 29871924 PMC6065170

[R116] Raha D, Wilson TR, Peng J (2014). The cancer stem cell marker aldehyde dehydrogenase is required to maintain a drugtolerant tumor cell subpopulation. Cancer Res 74, 3579–3590. doi:10.1158/0008-5472.can-13-3456 24812274

[R117] Reynier M (1969). Pyrazole inhibition and kinetic studies of ethanol and retinol oxidation catalyzed by rat liver alcohol dehydrogenase. Acta Chem Scand 23, 1119–1129. doi:10.3891/acta.chem.scand.23-1119 4979887

[R118] Richard AM, Judson RS, Houck KA (2016). ToxCast chemical landscape: Paving the road to 21^st^ century toxicology. Chem Res Toxicol 29, 1225–1251. doi:10.1021/acs.chemrestox.6b00135 27367298

[R119] Rowbotham SE, Illingworth NA, Daly AK (2010). Role of UDP-glucuronosyltransferase isoforms in 13-cis retinoic acid metabolism in humans. Drug Metab Dispos 38, 1211–1217. doi:10.1124/dmd.109.031625 20308471

[R120] Ruberte E, Friederich V, Morriss-Kay G (1992). Differential distribution patterns of CRABP I and CRABP II transcripts during mouse embryogenesis. Development 115, 973–987. doi: 10.1242/dev.115.4.973 1333403

[R121] Schindler JF, Berst KB and Plapp BV (1998). Inhibition of human alcohol dehydrogenases by formamides. J Med Chem 41, 1696–1701. doi:10.1021/jm9707380 9572895

[R122] Scialli AR, Daston G, Chen C (2018). Rethinking developmental toxicity testing: Evolution or revolution? Birth Defects Res 110, 840–850. doi:10.1002/bdr2.1212 29436169 PMC6624839

[R123] Shao ZM, Dawson MI, Li XS (1995). P53 independent G0/G1 arrest and apoptosis induced by a novel retinoid in human breast cancer cells. Oncogene 11, 493–504. 7630633

[R124] Sharma V, Sharma A, Kumar V (2009). Disulfiram-like reaction with ornidazole. J Postgrad Med 55, 292–293. doi:10.4103/0022-3859.58940 20083883

[R125] Shimomura T, Kawakami M, Okuda H (2015). Retinoic acid regulates Lhx8 expression via FGF-8b to the upper jaw development of chick embryo. J Biosci Bioeng 119, 260–266. doi:10.1016/j.jbiosc.2014.08.010 25239070

[R126] Silvaroli JA, Widjaja-Adhi MAK, Trischman T (2019). Abnormal cannabidiol modulates vitamin A metabolism by acting as a competitive inhibitor of CRBP1. ACS Chem Biol 14, 434–448. doi:10.1021/acschembio.8b01070 30721022 PMC6420351

[R127] Silvaroli JA, Plau J, Adams CH (2021). Molecular basis for the interaction of cellular retinol binding protein 2 (CRBP2) with nonretinoid ligands. J Lipid Res 62, 100054. doi:10.1016/j.jlr.2021.100054 33631211 PMC8010219

[R128] Singh AK and Dominic CJ (1995). Testicular toxicity of WIN 18446 in the laboratory mouse. Reprod Toxicol 9, 475–481. doi:10.1016/0890-6238(95)00039-d 8563190

[R129] Song S and Xu XC (2001). Effect of benzo[a]pyrene diol epoxide on expression of retinoic acid receptor-beta in immortalized esophageal epithelial cells and esophageal cancer cells. Biochem Biophys Res Commun 281, 872–877. doi:10.1006/bbrc.2001.4433 11237740

[R130] Song Y, Hui JN, Fu KK (2004). Control of retinoic acid synthesis and FGF expression in the nasal pit is required to pattern the craniofacial skeleton. Dev Biol 276, 313–329. doi:10.1016/j.ydbio.2004.08.035 15581867

[R131] Su Y, Li H, Chen X (2018). Ziram inhibits rat neurosteroidogenic 5α-reductase 1 and 3α-hydroxysteroid dehydrogenase. Toxicol Mech Methods 28, 38–44. doi:10.1080/15376516.2017.1355950 28707553

[R132] Sun X, Zhang Z, Ning H (2017). Sitagliptin down-regulates retinol-binding protein 4 and reduces insulin resistance in gestational diabetes mellitus: A randomized and double-blind trial. Metab Brain Dis 32, 773–778. doi:10.1007/s11011-017-9958-7 28213841

[R133] Takahashi M, Yang XJ, Lavery TT (2002). Gene expression profiling of favorable histology Wilms tumors and its correlation with clinical features. Cancer Res 62, 6598–6605. 12438255

[R134] Tan J, Thiboutot D, Popp G (2019). Randomized phase 3 evaluation of trifarotene 50 μg/g cream treatment of moderate facial and truncal acne. J Am Acad Dermatol 80, 1691–1699. doi:10.1016/j.jaad.2019.02.044 30802558

[R135] Tanaka M, Tamura K and Ide H (1996). Citral, an inhibitor of retinoic acid synthesis, modifies chick limb development. Dev Biol 175, 239–247. doi:10.1006/dbio.1996.0111 8626029

[R136] Thacher SM, Nagpal S, Klein ES (1999). Cell type and gene-specific activity of the retinoid inverse agonist AGN 193109: Divergent effects from agonist at retinoic acid receptor gamma in human keratinocytes. Cell Growth Differ 10, 255–262. 10319995

[R137] Thanacoody RH, Gilfillan C, Bradberry SM (2016). Management of poisoning with ethylene glycol and methanol in the UK: A prospective study conducted by the national poisons information service (NPIS). Clin Toxicol (Phila) 54, 134–140. doi:10.3109/15563650.2015.1116044 26594941

[R138] Thatcher JE, Buttrick B, Shaffer SA (2011). Substrate specificity and ligand interactions of CYP26A1, the human liver retinoic acid hydroxylase. Mol Pharmacol 80, 228–239. doi:10.1124/mol.111.072413 21521770 PMC3141886

[R139] Thomas ML, de Antueno R, Coyle KM (2016). Citral reduces breast tumor growth by inhibiting the cancer stem cell marker ALDH1A3. Mol Oncol 10, 1485–1496. doi:10.1016/j.molonc.2016.08.004 27592281 PMC5423215

[R140] Thoreau E, Arlabosse JM, Bouix-Peter C (2018). Structure-based design of trifarotene (CD5789), a potent and selective RARγ agonist for the treatment of acne. Bioorg Med Chem Lett 28, 1736–1741. doi:10.1016/j.bmcl.2018.04.036 29706423

[R141] USEPA (2018). Strategic Plan to Promote the Development and Implementation of Alternative Test Methods within the TSCA Program. U.S. Environmental Protection Agency, Office of Chemical Safety and Pollution Prevention, Washington, DC. https://www.epa.gov/sites/default/files/2018-06/documents/epa_alt_strat_plan_6-20-18_clean_final.pdf

[R142] Vandersea MW, Fleming P, McCarthy RA (1998). Fin duplications and deletions induced by disruption of retinoic acid signaling. Dev Genes Evol 208, 61–68. doi:10.1007/s004270050155 9569347

[R143] Venkataramaiah TH and Plapp BV (2003). Formamides mimic aldehydes and inhibit liver alcohol dehydrogenases and ethanol metabolism. J Biol Chem 278, 36699–36706. doi:10.1074/jbc.m305419200 12855684

[R144] Vermot J, Schuhbaur B, Le Mouellic H (2005). Retinaldehyde dehydrogenase 2 and Hoxc8 are required in the murine brachial spinal cord for the specification of Lim1+ motoneurons and the correct distribution of Islet1+ motoneurons. Development 132, 1611–1621. doi:10.1242/dev.01718 15753214

[R145] Villeneuve DL, Crump D, Garcia-Reyero N (2014). Adverse outcome pathway (AOP) development I: Strategies and principles. Toxicol Sci 142, 312–320. doi:10.1093/toxsci/kfu199 25466378 PMC4318923

[R146] Wang B, Yan Y, Zhou J (2013). A novel all-trans retinoid acid derivatives inhibits the migration of breast cancer cell lines MDA-MB-231 via myosin light chain kinase involving p38-MAPK pathway. Biomed Pharmacother 67, 357–362. doi:10.1016/j.biopha.2013.03.016 23602051

[R147] Wang C, Kane MA and Napoli JL (2011). Multiple retinol and retinal dehydrogenases catalyze all-trans-retinoic acid biosynthesis in astrocytes. J Biol Chem 286, 6542–6553. doi:10.1074/jbc.m110.198382 21138835 PMC3283052

[R148] Wang NN, Wang LH, Li Y (2018). Targeting ALDH2 with disulfiram/copper reverses the resistance of cancer cells to microtubule inhibitors. Exp Cell Res 362, 72–82. doi:10.1016/j.yexcr.2017.11.004 29155365

[R149] Wang Y, Connors R, Fan P (2014). Structure-assisted discovery of the first non-retinoid ligands for retinol-binding protein 4. Bioorg Med Chem Lett 24, 2885–2891. doi:10.1016/j.bmcl.2014.04.089 24835984

[R150] Wang Y, Sun J, Chen L (2017). Effects of resveratrol on rat neurosteroid synthetic enzymes. Fitoterapia 122, 61–66. doi:10.1016/j.fitote.2017.08.005 28823883

[R151] Wei LN (2016). Cellular retinoic acid binding proteins: Genomic and non-genomic functions and their regulation. Subcell Biochem 81, 163–178. doi:10.1007/978-94-024-0945-1_6 27830504

[R152] Wiley MJ (1983). The pathogenesis of retinoic acid-induced vertebral abnormalities in golden Syrian hamster fetuses. Teratology 28, 341–353. doi:10.1002/tera.1420280306 6665734

[R153] Williams AJ, Grulke CM, Edwards J (2017). The CompTox chemistry dashboard: A community data resource for environmental chemistry. J Cheminform 9, 61. doi:10.1186/s13321-017-0247-6 29185060 PMC5705535

[R154] Xie PT and Hurley TD (1999). Methionine-141 directly influences the binding of 4-methylpyrazole in human sigma sigma alcohol dehydrogenase. Protein Sci 8, 2639–2644. doi:10.1110/ps.8.12.2639 10631979 PMC2144219

[R155] Xu J, Zhang M, Zhang X (2018). Contribution of hepatic retinaldehyde dehydrogenase induction to impairment of glucose metabolism by high-fat-diet feeding in C57BL/6J mice. Basic Clin Pharmacol Toxicol 123, 539–548. doi:10.1111/bcpt.13039 29753302

[R156] Yasgar A, Titus SA, Wang Y (2017). A high-content assay enables the automated screening and identification of small molecules with specific ALDH1A1-inhibitory activity. PLoS One 12, e0170937. doi:10.1371/journal.pone.0170937 PMC5271370 28129349

[R157] Yashiro K, Zhao X, Uehara M (2004). Regulation of retinoic acid distribution is required for proximodistal patterning and outgrowth of the developing mouse limb. Dev Cell 6, 411–422. doi:10.1016/s1534-5807(04)00062-0 15030763

[R158] Yu J, Gonzalez S, Martinez L (2003). Effects of retinoic acid on the neural crest-controlled organs of fetal rats. Pediatr Surg Int 19, 355–358. doi:10.1007/s00383-003-1010-9 12898162

[R159] Zhang J, Pu K, Bai S (2020). The anti-alcohol dependency drug disulfiram inhibits the viability and progression of gastric cancer cells by regulating the Wnt and NF-κB pathways. J Int Med Res 48, 300060520925996. doi:10.1177/0300060520925996 PMC7294493 32529870

[R160] Zhang L, Nadzan AM, Heyman RA (1996). Discovery of novel retinoic acid receptor agonists having potent antiproliferative activity in cervical cancer cells. J Med Chem 39, 2659–2663. doi:10.1021/jm960285j 8709094

[R161] Zhong M, Kawaguchi R, Costabile B (2020). Regulatory mechanism for the transmembrane receptor that mediates bidirectional vitamin A transport. Proc Natl Acad Sci U S A 117, 9857–9864. doi:10.1073/pnas.1918540117 32300017 PMC7211970

