# Supplementary material for: Identifying Candidate Reference Chemicals for In Vitro Testing of the Retinoid Pathway for Predictive Developmental Toxicity
Source: ALTEX. Author manuscript; Available in PMC 2024 Jan 4. (PMC10765368; doi:10.14573/altex.2202231)
Supplement: Supplement1 [file NIHMS1945822-supplement-Supplement1.docx]

## Identifying candidate reference chemicals for *in vitro* testing of the retinoid pathway for predictive developmental toxicity

### Supplemental Material

Abbreviations Used
AC50: Concentration at which there is 50% activity, CAS RN: Chemical Abstracts Service Registry Number, Chem Ct: Chemical Count, DSSTOXID: EPA DSSTox database identification number, N/A: Not Applicable, PDB ID: Protein Data Bank Identification Number, PMID: PubMed Identifier, Year Dep: Year Deposited

Table of Contents

Table S1. Summary of Protein Data Bank records for retinol binding protein (RBP) serum/plasma

Table S2. Summary of ChEMBL entries for retinol binding protein (RBP) serum/plasma

Table S3. Protein Data Bank entries for STRA6

Table S4. Summary of Protein Data Bank records for Cellular Retinol Binding Protein (CRBP)

Table S5. Summary of Protein Data Bank results for Cellular Retinoic Acid Binding Proteins (CRABP1 and CRABP2)

Table S6. Summary of ChEMBL results for Cellular Retinoic Acid Binding Proteins (CRABP1 and CRABP2)

Table S7. Summary of ChEMBL entries for CYP26

Table S8. Summary of ChEMBL records returned by a search on “retinol dehydrogenase”

Table S9. Summary of Protein Data Bank records for Aldehyde Dehydrogenase (ALDH1A1, ALDH1A2, ALDH1A3)

Table S10. Summary of ChEMBL results for Aldehyde Dehydrogenase (ALDH1A1, ALDH1A2, ALDH1A3)

Table S11. Summary of Protein Data Bank entries for Retinoic acid receptor alpha (RARa)

Table S12. Summary of ChEMBL results for Retinoic acid receptor alpha (RARa)

Table S13. Summary of the Protein Data Bank records for Retinoic Acid Receptor beta (RARb)

Table S14. Summary of the ChEMBL records for Retinoic Acid Receptor beta (RARb)

Table S15. Summary of Protein Data Bank results for Retinoic Acid Receptor gamma (RARg)

Table S16. Summary of ChEMBL results for Retinoic Acid Receptor gamma (RARg)

Table S17. ToxCast and Tox21 results.

Table S18. Descriptions of ToxCast / Tox21 assays

### Table S1. Summary of Protein Data Bank records for retinol binding protein (RBP) serum/plasma

| PDB ID | Structure Title | Year Dep | PMID | Ligands Used |
| --- | --- | --- | --- | --- |
| [1AQB](http://www.rcsb.org/pdb/explore.do?structureId=1AQB) | RETINOL-BINDING PROTEIN (RBP) FROM PIG PLASMA | 1997 | [9757135](https://www.ncbi.nlm.nih.gov/pubmed/9757135) | CADMIUM ION / RETINOL |
| [1BRP](http://www.rcsb.org/pdb/explore.do?structureId=1BRP) | CRYSTAL STRUCTURE OF THE TRIGONAL FORM OF HUMAN PLASMA RETINOL-BINDING PROTEIN AT 2.5 ANGSTROMS RESOLUTION | 1992 | [8464067](https://www.ncbi.nlm.nih.gov/pubmed/8464067) | RETINOL |
| [1BRQ](http://www.rcsb.org/pdb/explore.do?structureId=1BRQ) | CRYSTAL STRUCTURE OF THE TRIGONAL FORM OF HUMAN PLASMA RETINOL-BINDING PROTEIN AT 2.5 ANGSTROMS RESOLUTION | 1992 | [8464067](https://www.ncbi.nlm.nih.gov/pubmed/8464067) | N/A |
| [1ERB](http://www.rcsb.org/pdb/explore.do?structureId=1ERB) | THE INTERACTION OF N-ETHYL RETINAMIDE WITH PLASMA RETINOL-BINDING PROTEIN (RBP) AND THE CRYSTAL STRUCTURE OF THE RETINOID-RBP COMPLEX AT 1.9 ANGSTROMS RESOLUTION | 1993 | [8227049](https://www.ncbi.nlm.nih.gov/pubmed/8227049) | N-ETHYL RETINAMIDE |
| [1FEL](http://www.rcsb.org/pdb/explore.do?structureId=1FEL) | CRYSTALLOGRAPHIC STUDIES ON COMPLEXES BETWEEN RETINOIDS AND PLASMA RETINOL-BINDING PROTEIN | 1994 | [7961949](https://www.ncbi.nlm.nih.gov/pubmed/7961949) | N-(4-HYDROXYPHENYL)ALL-TRANS RETINAMIDE |
| [1FEM](http://www.rcsb.org/pdb/explore.do?structureId=1FEM) | CRYSTALLOGRAPHIC STUDIES ON COMPLEXES BETWEEN RETINOIDS AND PLASMA RETINOL-BINDING PROTEIN | 1994 | [7961949](https://www.ncbi.nlm.nih.gov/pubmed/7961949) | RETINOIC ACID |
| [1FEN](http://www.rcsb.org/pdb/explore.do?structureId=1FEN) | CRYSTALLOGRAPHIC STUDIES ON COMPLEXES BETWEEN RETINOIDS AND PLASMA RETINOL-BINDING PROTEIN | 1994 | [7961949](https://www.ncbi.nlm.nih.gov/pubmed/7961949) | ALL-TRANS AXEROPHTHENE |
| [1HBP](http://www.rcsb.org/pdb/explore.do?structureId=1HBP) | CRYSTAL STRUCTURE OF LIGANDED AND UNLIGANDED FORMS OF BOVINE PLASMA RETINOL-BINDING PROTEIN | 1993 | [8496140](https://www.ncbi.nlm.nih.gov/pubmed/8496140) | RETINOL |
| [1HBQ](http://www.rcsb.org/pdb/explore.do?structureId=1HBQ) | CRYSTAL STRUCTURE OF LIGANDED AND UNLIGANDED FORMS OF BOVINE PLASMA RETINOL-BINDING PROTEIN | 1993 | [8496140](https://www.ncbi.nlm.nih.gov/pubmed/8496140) | N/A |
| [1IIU](http://www.rcsb.org/pdb/explore.do?structureId=1IIU) | Chicken plasma retinol-binding protein (RBP) | 2001 | [11738088](https://www.ncbi.nlm.nih.gov/pubmed/11738088) | CADMIUM ION / RETINOL |
| [1JYD](http://www.rcsb.org/pdb/explore.do?structureId=1JYD) | Crystal Structure of Recombinant Human Serum Retinol-Binding Protein at 1.7 A Resolution | 2001 | [11604536](https://www.ncbi.nlm.nih.gov/pubmed/11604536) | GLYCEROL |
| [1JYJ](http://www.rcsb.org/pdb/explore.do?structureId=1JYJ) | Crystal Structure of a Double Variant (W67L/W91H) of Recombinant Human Serum Retinol-binding Protein at 2.0 A Resolution | 2001 | [11604536](https://www.ncbi.nlm.nih.gov/pubmed/11604536) | GLYCEROL |
| [1KT3](http://www.rcsb.org/pdb/explore.do?structureId=1KT3) - [1KT7](http://www.rcsb.org/pdb/explore.do?structureId=1KT7) | Crystal structure of bovine holo-RBP at pH 2.0 | 2002 | [12787682](https://www.ncbi.nlm.nih.gov/pubmed/12787682) | RETINOL |
| [1OO2](http://www.rcsb.org/pdb/explore.do?structureId=1OO2) | Crystal structure of transthyretin from Sparus aurata | 2003 | [14644428](https://www.ncbi.nlm.nih.gov/pubmed/14644428) | CADMIUM ION |
| [1RBP](http://www.rcsb.org/pdb/explore.do?structureId=1RBP) | CRYSTALLOGRAPHIC REFINEMENT OF HUMAN SERUM RETINOL BINDING PROTEIN AT 2 ANGSTROMS RESOLUTION | 1990 | [2217163](https://www.ncbi.nlm.nih.gov/pubmed/2217163) | RETINOL |
| [1RLB](http://www.rcsb.org/pdb/explore.do?structureId=1RLB) | RETINOL BINDING PROTEIN COMPLEXED WITH TRANSTHYRETIN | 1995 | [7754382](https://www.ncbi.nlm.nih.gov/pubmed/7754382) | RETINOIC ACID |
| [1THC](http://www.rcsb.org/pdb/explore.do?structureId=1THC) | CRYSTAL STRUCTURE DETERMINATION AT 2.3A OF HUMAN TRANSTHYRETIN-3',5'-DIBROMO-2',4,4',6-TETRA-HYDROXYAURONE COMPLEX | 1992 | [1631168](https://www.ncbi.nlm.nih.gov/pubmed/1631168) | 3',5'-DIBROMO-2',4,4',6'-TETRAHYDROXY AURONE |
| [1TTR](http://www.rcsb.org/pdb/explore.do?structureId=1TTR) | TRANSTHYRETIN-V/122/I CARDIOMYOPATHIC MUTANT | 1996 | [15299606](https://www.ncbi.nlm.nih.gov/pubmed/15299606) | N/A |
| [1TYR](http://www.rcsb.org/pdb/explore.do?structureId=1TYR) | TRANSTHYRETIN COMPLEX WITH RETINOIC ACID | 1995 | [8536704](https://www.ncbi.nlm.nih.gov/pubmed/8536704) | (9cis)-retinoic acid |
| [2WQ9](http://www.rcsb.org/pdb/explore.do?structureId=2WQ9) | Crystal Structure of RBP4 bound to Oleic Acid | 2009 | N/A | CHLORIDE ION / GLYCEROL / OLEIC ACID |
| [2WQA](http://www.rcsb.org/pdb/explore.do?structureId=2WQA) | Complex of TTR and RBP4 and Oleic Acid | 2009 | N/A | OLEIC ACID / SULFATE ION |
| [2WR6](http://www.rcsb.org/pdb/explore.do?structureId=2WR6) | Structure of the complex of RBP4 with linoleic acid | 2009 | N/A | (11E,13E,15Z)-OCTADECA-11,13,15-TRIENOIC ACID / CHLORIDE ION |
| [3BSZ](http://www.rcsb.org/pdb/explore.do?structureId=3BSZ) | Crystal structure of the transthyretin-retinol binding protein-Fab complex | 2007 | [7754382](https://www.ncbi.nlm.nih.gov/pubmed/7754382)/ [10052934](https://www.ncbi.nlm.nih.gov/pubmed/10052934) / [10089423](https://www.ncbi.nlm.nih.gov/pubmed/10089423) / [19021760](https://www.ncbi.nlm.nih.gov/pubmed/19021760) | RETINOL |
| [3FMZ](http://www.rcsb.org/pdb/explore.do?structureId=3FMZ) | Crystal Structure of Retinol-Binding Protein 4 (RBP4) in complex with non-retinoid ligand | 2008 | [19147488](https://www.ncbi.nlm.nih.gov/pubmed/19147488) | 2-[({4-[2-(trifluoromethyl)phenyl]piperidin-1-yl}carbonyl)amino]benzoic acid (A1120) |
| [3HJ0](http://www.rcsb.org/pdb/explore.do?structureId=3HJ0) | Transthyretin in complex with a covalent small molecule kinetic stabilizer | 2009 | [20081815](https://www.ncbi.nlm.nih.gov/pubmed/20081815) | 3-SULFINOALANINE / 4-fluorophenyl 3-[(E)-2-(4-hydroxy-3,5-dimethylphenyl)ethenyl]benzoate |
| [4O9S](http://www.rcsb.org/pdb/explore.do?structureId=4O9S) | Crystal structure of Retinol-Binding Protein 4 (RBP4) in complex with a non-retinoid ligand | 2014 | [24835984](https://www.ncbi.nlm.nih.gov/pubmed/24835984) | 1,2-ETHANEDIOL / 1-[4-(8lambda~4~-cyclopenta[4,5]thieno[2,3-d]pyrimidin-4-yl)piperazin-1-yl]-2-[2-(trifluoromethyl)phenyl]ethanone / CHLORIDE ION / DI(HYDROXYETHYL)ETHER |
| [4PSQ](http://www.rcsb.org/pdb/explore.do?structureId=4PSQ) | Crystal Structure of Retinol-Binding Protein 4 (RBP4) in complex with a non-retinoid ligand | 2014 | [24835984](https://www.ncbi.nlm.nih.gov/pubmed/24835984) | (1-benzyl-1H-imidazol-4-yl)[4-(2-chlorophenyl)piperazin-1-yl]methanone / 1,2-ETHANEDIOL / PHOSPHATE ION |
| [5NTY](http://www.rcsb.org/pdb/explore.do?structureId=5NTY) | Structure of non-fluorescent human plasma RBP4 | 2017 | [29414511](https://www.ncbi.nlm.nih.gov/pubmed/29414511) | CHLORIDE ION / PALMITIC ACID |
| [5NU2](http://www.rcsb.org/pdb/explore.do?structureId=5NU2) | Structure of non-fluorescent human urine RBP4 | 2017 | [29414511](https://www.ncbi.nlm.nih.gov/pubmed/29414511) | CHLORIDE ION / PALMITIC ACID |
| [5NU6](http://www.rcsb.org/pdb/explore.do?structureId=5NU6) | Structure of non-fluorescent human amniotic fluid RBP4 | 2017 | [29414511](https://www.ncbi.nlm.nih.gov/pubmed/29414511) | CHLORIDE ION / PALMITIC ACID |
| [5NU7](http://www.rcsb.org/pdb/explore.do?structureId=5NU7) | Structure of human holo plasma RBP4 | 2017 | [29414511](https://www.ncbi.nlm.nih.gov/pubmed/29414511) | CHLORIDE ION / RETINOL |
| [5NU8](http://www.rcsb.org/pdb/explore.do?structureId=5NU8) | Structure of human urine RBP4 saturated with palmitate | 2017 | [29414511](https://www.ncbi.nlm.nih.gov/pubmed/29414511) | CHLORIDE ION / PALMITIC ACID |
| [5NU9](http://www.rcsb.org/pdb/explore.do?structureId=5NU9) | Structure of human amniotic fluid RBP4 saturated with palmitate | 2017 | [29414511](https://www.ncbi.nlm.nih.gov/pubmed/29414511) | CHLORIDE ION / PALMITIC ACID |
| [5NUA](http://www.rcsb.org/pdb/explore.do?structureId=5NUA) | Structure of human urine RBP4 saturated with laurate | 2017 | [29414511](https://www.ncbi.nlm.nih.gov/pubmed/29414511) | CHLORIDE ION / LAURIC ACID |
| [5NUB](http://www.rcsb.org/pdb/explore.do?structureId=5NUB) | Structure of human amniotic fluid RBP4 saturated with laurate | 2017 | [29414511](https://www.ncbi.nlm.nih.gov/pubmed/29414511) | CHLORIDE ION / LAURIC ACID |

### Table S2. Summary of ChEMBL entries for retinol binding protein (RBP) serum/plasma

| Assay ID | Chem  Ct | Assay Description | PMID |
| --- | --- | --- | --- |
| [CHEMBL3889136](https://www.ebi.ac.uk/chembl/assay/inspect/CHEMBL3889136) | 123 | TR-FRET Assay: TR-FRET assay for retinol-induced RBP4-TTR interaction Binding of a desired RBP4 antagonist displaces retinol and induces hindrance for RBP4-TTR interaction resulting in the decreased FRET signal. | N/A |
| [CHEMBL3889135](https://www.ebi.ac.uk/chembl/assay/inspect/CHEMBL3889135) | 124 | Scintillation Proximity Binding Assay: Untagged human RBP4 purified from urine of tubular proteinuria patients was purchased from Fitzgerald Industries International. It was biotinylated using the EZ-Link Sulfo-NHS-LC-Biotinylation kit from Pierce following the manufacturer's recommendations. Binding experiments were performed in 96-well plates (OptiPlate, PerkinElmer) in a final assay volume of 100 ul per well in SPA buffer (IX PBS, pH 7.4, 1 mM EDTA, 0.1% BSA, 0.5% CHAPS). | N/A |
| [CHEMBL3705730](https://www.ebi.ac.uk/chembl/assay/inspect/CHEMBL3705730) | 17 | Binding Assay: Streptavidin (20 ul) (10 ug/ml Streptavidin type II (Wako Pure Chemical Industries, Ltd.), 10 mM Tris-HCl (pH 7.5), 10 mM NaCl) was added to a 384 well black plate (Nunc MaxiSorp, Thermo Fisher Scientific Inc.), and the plate was subjected to centrifugation (1000 rpm, 1 min) and coated overnight at 4 C. | N/A |
| [CHEMBL3705134](https://www.ebi.ac.uk/chembl/assay/inspect/CHEMBL3705134) | 15 | Binding Assay: The action of the compound of the present invention to inhibit binding of RBP4 and retinol and TTR was evaluated using the Retinol-RBP4-TTR ELISA (human-type ELISA) system. | N/A |
| [CHEMBL3594968](https://www.ebi.ac.uk/chembl/assay/inspect/CHEMBL3594968) | 35 | Inhibition of retinol-induced interaction of bacterially expressed MBP-tagged RBP4 (unknown origin) with Eu3+ cryptate labeled TTR by HTRF assay | [26181715](https://www.ncbi.nlm.nih.gov/pubmed/26181715) |
| [CHEMBL3594967](https://www.ebi.ac.uk/chembl/assay/inspect/CHEMBL3594967) | 36 | Displacement of [3H]-retinol from RBP4 (unknown origin) by scintillation proximity assay | [26181715](https://www.ncbi.nlm.nih.gov/pubmed/26181715) |
| [CHEMBL3380010](https://www.ebi.ac.uk/chembl/assay/inspect/CHEMBL3380010) | 35 | Antagonist activity at maltose binding protein-tagged RBP4 (unknown origin) expressed in Escherichia coli assessed as inhibition of retinol-induced protein/transthyretin interaction by HTRF assay | [25210858](https://www.ncbi.nlm.nih.gov/pubmed/25210858) |
| [CHEMBL3380009](https://www.ebi.ac.uk/chembl/assay/inspect/CHEMBL3380009) | 35 | Displacement of [3H]retinol from biotinylated human RBP4 by scintillation proximity assay | [25210858](https://www.ncbi.nlm.nih.gov/pubmed/25210858) |
| [CHEMBL3291833](https://www.ebi.ac.uk/chembl/assay/inspect/CHEMBL3291833) | 26 | Inhibition of human recombinant RBP4-transthyretin interaction by FRET analysis | [24835984](https://www.ncbi.nlm.nih.gov/pubmed/24835984) |
| [CHEMBL3291832](https://www.ebi.ac.uk/chembl/assay/inspect/CHEMBL3291832) | 33 | Displacement of [3H]-retinol from human recombinant RBP4 by scintillation proximity assay | [24835984](https://www.ncbi.nlm.nih.gov/pubmed/24835984) |
| [CHEMBL1804573](https://www.ebi.ac.uk/chembl/assay/inspect/CHEMBL1804573) | 7 | Binding affinity to immobilized His-tagged recombinant human sRBP assessed as inhibition of sRBP and detergent treated HEK293 membrane interaction at 100 uM after 1 hr by surface plasmon resonance assay | [21591606](https://www.ncbi.nlm.nih.gov/pubmed/21591606) |
| [CHEMBL1804572](https://www.ebi.ac.uk/chembl/assay/inspect/CHEMBL1804572) | 2 | Antagonist activity at His-tagged recombinant human sRBP expressed in Escherichia coli BL21(DE3) assessed as disruption of sRBP-TTR protein interaction after 2 hr by surface plasmon resonance assay | [21591606](https://www.ncbi.nlm.nih.gov/pubmed/21591606) |
| [CHEMBL1804571](https://www.ebi.ac.uk/chembl/assay/inspect/CHEMBL1804571) | 7 | Antagonist activity at His-tagged recombinant human sRBP expressed in Escherichia coli BL21(DE3) assessed as disruption of ROH-sRBP-TTR protein interaction after 2 hr by TR-FRET assay relative to control | [21591606](https://www.ncbi.nlm.nih.gov/pubmed/21591606) |
| [CHEMBL1804187](https://www.ebi.ac.uk/chembl/assay/inspect/CHEMBL1804187) | 7 | Antagonist activity at His-tagged recombinant human sRBP expressed in Escherichia coli BL21(DE3) assessed as disruption of ROH-sRBP-TTR protein interaction after 2 hr by TR-FRET assay | [21591606](https://www.ncbi.nlm.nih.gov/pubmed/21591606) |
| [CHEMBL1804186](https://www.ebi.ac.uk/chembl/assay/inspect/CHEMBL1804186) | 1 | Binding affinity to His-tagged recombinant human sRBP expressed in Escherichia coli BL21(DE3) assessed as disruption of sRBP-TTR protein interaction at 100 uM after 1 hr by SDS-PAGE and silver staining method relative to 10 uM ROH | [21591606](https://www.ncbi.nlm.nih.gov/pubmed/21591606) |
| [CHEMBL1804185](https://www.ebi.ac.uk/chembl/assay/inspect/CHEMBL1804185) | 47 | Binding affinity to His-tagged recombinant human sRBP expressed in Escherichia coli BL21(DE3) assessed as disruption of sRBP-TTR protein interaction after 1 hr by SDS-PAGE and silver staining method | [21591606](https://www.ncbi.nlm.nih.gov/pubmed/21591606) |
| [CHEMBL1804184](https://www.ebi.ac.uk/chembl/assay/inspect/CHEMBL1804184) | 21 | Binding affinity to His-tagged recombinant human sRBP expressed in Escherichia coli BL21(DE3) assessed as disruption of sRBP-TTR protein interaction at 1 to 100 uM after 1 hr by SDS-PAGE and silver staining method | [21591606](https://www.ncbi.nlm.nih.gov/pubmed/21591606) |
| [CHEMBL1804183](https://www.ebi.ac.uk/chembl/assay/inspect/CHEMBL1804183) | 8 | Binding affinity to His-tagged recombinant human sRBP expressed in Escherichia coli BL21(DE3) assessed as apparent dissociation constant after 5 mins by fluorescence spectrophotometric analysis | [21591606](https://www.ncbi.nlm.nih.gov/pubmed/21591606) |

### Table S3. Protein Data Bank entries for STRA6

| PDB ID | Structure Title | Year Dep | PMID | Ligands used |
| --- | --- | --- | --- | --- |
| [5K8Q](http://www.rcsb.org/pdb/explore.do?structureId=5K8Q) | Crystal Structure of Calcium-loaded Calmodulin in complex with STRA6 CaMBP2-site peptide. | 2016 | [27563101](https://www.ncbi.nlm.nih.gov/pubmed/27563101) | IMIDAZOLE /CALCIUM ION / AMINO GROUP / ACETYL GROUP |
| [5SY1](http://www.rcsb.org/pdb/explore.do?structureId=5SY1) | Structure of the STRA6 receptor for retinol uptake in complex with calmodulin | 2016 | [27563101](https://www.ncbi.nlm.nih.gov/pubmed/27563101) | CALCIUM ION / CHOLESTEROL |

### Table S4. Summary of Protein Data Bank records for Cellular Retinol Binding Protein (CRBP)

| PDB Link | Structure Title | PMID | Year Dep | Ligands Used |
| --- | --- | --- | --- | --- |
| [1B4M](http://www.rcsb.org/pdb/explore.do?structureId=1B4M) | NMR STRUCTURE OF APO CELLULAR RETINOL-BINDING PROTEIN II, 24 STRUCTURES | [10047490](https://www.ncbi.nlm.nih.gov/pubmed/10047490) | 1998 | N/A |
| [1CBQ](http://www.rcsb.org/pdb/explore.do?structureId=1CBQ) | CRYSTAL STRUCTURE OF CELLULAR RETINOIC-ACID-BINDING PROTEINS I AND II IN COMPLEX WITH ALL-TRANS-RETINOIC ACID AND A SYNTHETIC RETINOID | [7704533](https://www.ncbi.nlm.nih.gov/pubmed/7704533) | 1994 | 6-(2,3,4,5,6,7-HEXAHYDRO-2,4,4-TRIMETHYL-1-METYLENEINDEN-2-YL)-3-METHYLHEXA-2,4-DIENOIC ACID / PHOSPHATE ION |
| [1CBR](http://www.rcsb.org/pdb/explore.do?structureId=1CBR) | CRYSTAL STRUCTURE OF CELLULAR RETINOIC-ACID-BINDING PROTEINS I AND II IN COMPLEX WITH ALL-TRANS-RETINOIC ACID AND A SYNTHETIC RETINOID | [7704533](https://www.ncbi.nlm.nih.gov/pubmed/7704533) | 1994 | RETINOIC ACID |
| [1CBS](http://www.rcsb.org/pdb/explore.do?structureId=1CBS) | CRYSTAL STRUCTURE OF CELLULAR RETINOIC-ACID-BINDING PROTEINS I AND II IN COMPLEX WITH ALL-TRANS-RETINOIC ACID AND A SYNTHETIC RETINOID | [7704533](https://www.ncbi.nlm.nih.gov/pubmed/7704533) | 1994 | RETINOIC ACID |
| [1CRB](http://www.rcsb.org/pdb/explore.do?structureId=1CRB) | CRYSTALLOGRAPHIC STUDIES ON A FAMILY OF CELLULAR LIPOPHILIC TRANSPORT PROTEINS. REFINEMENT OF P2 MYELIN PROTEIN AND THE STRUCTURE DETERMINATION AND REFINEMENT OF CELLULAR RETINOL-BINDING PROTEIN IN COMPLEX WITH ALL-TRANS-RETINOL | [7683727](https://www.ncbi.nlm.nih.gov/pubmed/7683727) | 1993 | CADMIUM ION / RETINOL |
| [1EII](http://www.rcsb.org/pdb/explore.do?structureId=1EII) | NMR STRUCTURE OF HOLO CELLULAR RETINOL-BINDING PROTEIN II | [10884357](https://www.ncbi.nlm.nih.gov/pubmed/10884357) | 2000 | RETINOL |
| [1GGL](http://www.rcsb.org/pdb/explore.do?structureId=1GGL) | HUMAN CELLULAR RETINOL BINDING PROTEIN III | [11274389](https://www.ncbi.nlm.nih.gov/pubmed/11274389) | 2000 | N/A |
| [1JBH](http://www.rcsb.org/pdb/explore.do?structureId=1JBH) | Solution structure of cellular retinol binding protein type-I in the ligand-free state | [11934897](https://www.ncbi.nlm.nih.gov/pubmed/11934897) | 2001 | N/A |
| [1KGL](http://www.rcsb.org/pdb/explore.do?structureId=1KGL) | Solution structure of cellular retinol binding protein type-I in complex with all-trans-retinol | [11934897](https://www.ncbi.nlm.nih.gov/pubmed/11934897) | 2001 | RETINOL |
| [1KQW](http://www.rcsb.org/pdb/explore.do?structureId=1KQW) ,  [1KQX](http://www.rcsb.org/pdb/explore.do?structureId=1KQX) | Crystal structure of holo-CRBP from zebrafish | [12162964](https://www.ncbi.nlm.nih.gov/pubmed/12162964) | 2002 | RETINOL |
| [1LPJ](http://www.rcsb.org/pdb/explore.do?structureId=1LPJ) | Human cRBP IV | [12177003](https://www.ncbi.nlm.nih.gov/pubmed/12177003) | 2002 | N/A |
| [1MX7](http://www.rcsb.org/pdb/explore.do?structureId=1MX7) | Two homologous rat cellular retinol-binding proteins differ in local structure and flexibility | [12850148](https://www.ncbi.nlm.nih.gov/pubmed/12850148) | 2002 | N/A |
| [1MX8](http://www.rcsb.org/pdb/explore.do?structureId=1MX8) | Two homologous rat cellular retinol-binding proteins differ in local structure and flexibility | [12850148](https://www.ncbi.nlm.nih.gov/pubmed/12850148) | 2002 | RETINOL |
| [1OPA](http://www.rcsb.org/pdb/explore.do?structureId=1OPA) | THE CRYSTAL STRUCTURES OF HOLO-AND APO-CELLULAR RETINOL BINDING PROTEIN II | [8487303](https://www.ncbi.nlm.nih.gov/pubmed/8487303) | 1992 | N/A |
| [1OPB](http://www.rcsb.org/pdb/explore.do?structureId=1OPB) | THE CRYSTAL STRUCTURES OF HOLO-AND APO-CELLULAR RETINOL BINDING PROTEIN II | [8487303](https://www.ncbi.nlm.nih.gov/pubmed/8487303) | 1992 | RETINAL |
| [1PMP](http://www.rcsb.org/pdb/explore.do?structureId=1PMP) | CRYSTALLOGRAPHIC STUDIES ON A FAMILY OF CELLULAR LIPOPHILIC TRANSPORT PROTEINS. REFINEMENT OF P2 MYELIN PROTEIN AND THE STRUCTURE DETERMINATION AND REFINEMENT OF CELLULAR RETINOL-BINDING PROTEIN IN COMPLEX WITH ALL-TRANS-RETINOL | [7683727](https://www.ncbi.nlm.nih.gov/pubmed/7683727) | 1993 | OLEIC ACID |
| [2RCQ](http://www.rcsb.org/pdb/explore.do?structureId=2RCQ) | Crystal structure of human apo Cellular Retinol Binding Protein II (CRBP-II) | [18076076](https://www.ncbi.nlm.nih.gov/pubmed/18076076) | 2007 | L(+)-TARTARIC ACID / SULFATE ION |
| [2RCT](http://www.rcsb.org/pdb/explore.do?structureId=2RCT) | Crystal structure of human holo cellular retinol-binding protein II (CRBP-II) | [18076076](https://www.ncbi.nlm.nih.gov/pubmed/18076076) | 2007 | L(+)-TARTARIC ACID / RETINOL / SULFATE ION |
| [3CR6](http://www.rcsb.org/pdb/explore.do?structureId=3CR6) | Crystal Structure of the R132K:R111L:A32E Mutant of Cellular Retinoic Acid Binding Protein Type II Complexed with C15-aldehyde (a retinal analog) at 1.22 Angstrom resolution. | N/A | 2008 | 1,3,3-trimethyl-2-[(1E,3E)-3-methylpenta-1,3-dien-1-yl]cyclohexene |
| [3CWK](http://www.rcsb.org/pdb/explore.do?structureId=3CWK) | Crystal Structure of the R132K:Y134F:R111L:T54V:L121E Mutant of Cellular Retinoic Acid Binding Protein Type II in Complex with All-trans-Retinoic Acid at 1.57 Angstroms Resolution | [19018099](https://www.ncbi.nlm.nih.gov/pubmed/19018099) | 2008 | RETINOIC ACID / SULFATE ION |
| [3D95](http://www.rcsb.org/pdb/explore.do?structureId=3D95) | Crystal Structure of the R132K:Y134F:R111L:L121E:T54V Mutant of Apo-Cellular Retinoic Acid Binding Protein Type II at 1.20 Angstroms Resolution | [19018099](https://www.ncbi.nlm.nih.gov/pubmed/19018099) | 2008 | N/A |
| [3D96](http://www.rcsb.org/pdb/explore.do?structureId=3D96) | Crystal Structure of the R132K:Y134F Mutant of Apo-Cellular Retinoic Acid Binding Protein Type II at 1.71 Angstroms Resolution | [19018099](https://www.ncbi.nlm.nih.gov/pubmed/19018099) | 2008 | ACETATE ION |
| [3D97](http://www.rcsb.org/pdb/explore.do?structureId=3D97) | Crystal Structure of the R132K:R111L:L121E Mutant of Apo-Cellular Retinoic Acid Binding Protein Type II At 1.50 Angstroms Resolution | N/A | 2008 | 2-[3-(2-HYDROXY-1,1-DIHYDROXYMETHYL-ETHYLAMINO)-PROPYLAMINO]-2-HYDROXYMETHYL-PROPANE-1,3-DIOL / SODIUM ION |
| [3F8A](http://www.rcsb.org/pdb/explore.do?structureId=3F8A) | Crystal Structure of the R132K:R111L:L121E:R59W Mutant of Cellular Retinoic Acid-Binding Protein Type II Complexed with C15-aldehyde (a retinal analog) at 1.95 Angstrom resolution. | N/A | 2008 | 1,3,3-trimethyl-2-[(1E,3E)-3-methylpenta-1,3-dien-1-yl]cyclohexene / 2-[3-(2-HYDROXY-1,1-DIHYDROXYMETHYL-ETHYLAMINO)-PROPYLAMINO]-2-HYDROXYMETHYL-PROPANE-1,3-DIOL |
| [3F9D](http://www.rcsb.org/pdb/explore.do?structureId=3F9D) | Crystal structure of the R132K:R111L:T54E mutant of cellular retinoic acid-binding protein II complexed with C15-aldehyde (a retinal analog) at 2.00 angstrom resolution | N/A | 2008 | 1,3,3-trimethyl-2-[(1E,3E)-3-methylpenta-1,3-dien-1-yl]cyclohexene |
| [3FA6](http://www.rcsb.org/pdb/explore.do?structureId=3FA6) | Crystal structure of the R132K:Y134F:R111L:L121D:T54V mutant of cellular retinoic acid-binding protein II complexed with C15-aldehyde (a retinal analog) at 1.54 angstrom resolution | N/A | 2008 | 1,3,3-trimethyl-2-[(1E,3E)-3-methylpenta-1,3-dien-1-yl]cyclohexene |
| [3FA7](http://www.rcsb.org/pdb/explore.do?structureId=3FA7) | Crystal structure of the apo R132K:R111L:L121E:R59E mutant of cellular retinoic acid-binding protein II at 1.90 angstrom resolution | N/A | 2008 | 2-[3-(2-HYDROXY-1,1-DIHYDROXYMETHYL-ETHYLAMINO)-PROPYLAMINO]-2-HYDROXYMETHYL-PROPANE-1,3-DIOL |
| [3FA8](http://www.rcsb.org/pdb/explore.do?structureId=3FA8) | Crystal structure of the apo R132K:Y134F:R111L:L121E mutant of cellular retinoic acid-binding protein II at 1.78 angstrom resolution | N/A | 2008 | N/A |
| [3FA9](http://www.rcsb.org/pdb/explore.do?structureId=3FA9) | Crystal structure of the apo R132K:Y134F:R111L:L121D mutant of cellular retinoic acid-binding protein II at 1.94 angstrom resolution | N/A | 2008 | ACETATE ION |
| [3FEK](http://www.rcsb.org/pdb/explore.do?structureId=3FEK) | Crystal structure of the R132K:Y134F:R111L:L121D:T54V mutant of cellular retinoic acid-binding protein II at 1.51 angstrom resolution | N/A | 2008 | ACETATE ION / DI(HYDROXYETHYL)ETHER |
| [3FEL](http://www.rcsb.org/pdb/explore.do?structureId=3FEL) | Crystal structure of the R132K:R111L:T54E mutant of cellular retinoic acid-binding protein II at 1.85 angstrom resolution | N/A | 2008 | 2-[3-(2-HYDROXY-1,1-DIHYDROXYMETHYL-ETHYLAMINO)-PROPYLAMINO]-2-HYDROXYMETHYL-PROPANE-1,3-DIOL |
| [3FEN](http://www.rcsb.org/pdb/explore.do?structureId=3FEN) | Crystal structure of the R132K:R111L:A32E mutant of cellular retinoic acid-binding protein II at 1.56 angstrom resolution | N/A | 2008 | CHLORIDE ION |
| [3FEP](http://www.rcsb.org/pdb/explore.do?structureId=3FEP) | Crystal structure of the R132K:R111L:L121E:R59W-CRABPII mutant complexed with a synthetic ligand (merocyanin) at 2.60 angstrom resolution. | [25534273](https://www.ncbi.nlm.nih.gov/pubmed/25534273) | 2008 | (2E,4E,6E)-3-methyl-6-(1,3,3-trimethyl-1,3-dihydro-2H-indol-2-ylidene)hexa-2,4-dienal / 2-(N-MORPHOLINO)-ETHANESULFONIC ACID |
| [3HX3](http://www.rcsb.org/pdb/explore.do?structureId=3HX3) | Crystal structure of CRALBP mutant R234W | [19846785](https://www.ncbi.nlm.nih.gov/pubmed/19846785) | 2009 | RETINAL / SELENOMETHIONINE |
| [3HY5](http://www.rcsb.org/pdb/explore.do?structureId=3HY5) | Crystal structure of CRALBP | [19846785](https://www.ncbi.nlm.nih.gov/pubmed/19846785) | 2009 | L(+)-TARTARIC ACID / RETINAL |
| [3I17](http://www.rcsb.org/pdb/explore.do?structureId=3I17) | Crystal structure of the apo R132K:L121E mutant of cellular retinoic acid-binding protein II at 1.68 angstrom resolution | [19603486](https://www.ncbi.nlm.nih.gov/pubmed/19603486) | 2009 | N/A |
| [4EDE](http://www.rcsb.org/pdb/explore.do?structureId=4EDE) | Crystal Structure of the Q108K:K40L:T51V:T53C:Y19W:R58W:T29L:A33W Mutant of Cellular Retinol Binding Protein Type II in Complex with All-trans-Retinal at 1.4 Angstrom Resolution | [23224553](https://www.ncbi.nlm.nih.gov/pubmed/23224553) | 2012 | ACETATE ION / RETINAL |
| [4EEJ](http://www.rcsb.org/pdb/explore.do?structureId=4EEJ) | Crystal Structure of the Q108K:K40L:T51V:T53C:Y19W:R58W:T29L:Q4R Mutant of Cellular Retinol Binding Protein Type II in Complex with All-trans-Retinal at 1.5 Angstrom Resolution | [23224553](https://www.ncbi.nlm.nih.gov/pubmed/23224553) | 2012 | ACETATE ION / RETINAL |
| [4EFG](http://www.rcsb.org/pdb/explore.do?structureId=4EFG) | Crystal Structure of the Q108K:K40L:T51V:T53C:Y19W:R58W:T29L Mutant of Cellular Retinol Binding Protein Type II in Complex with All-trans-Retinal at 1.58 Angstrom Resolution | [23224553](https://www.ncbi.nlm.nih.gov/pubmed/23224553) | 2012 | ACETATE ION / RETINAL |
| [4EXZ](http://www.rcsb.org/pdb/explore.do?structureId=4EXZ) | Crystal Structure of the Q108K:K40L Mutant of Cellular Retinol Binding Protein Type II in Complex with All-trans-Retinal at 1.7 Angstrom Resolution | [23224553](https://www.ncbi.nlm.nih.gov/pubmed/23224553) | 2012 | ACETATE ION / RETINAL |
| [4GKC](http://www.rcsb.org/pdb/explore.do?structureId=4GKC) | Crystal structure of Q108K:K40L:T51V:T53C:R58W:T29L:Y19W:Q4A mutant of cellular retinol binding protein II complex with all-trans-retinal at 1.33 | [23224553](https://www.ncbi.nlm.nih.gov/pubmed/23224553) | 2012 | RETINAL |
| [4HIQ](http://www.rcsb.org/pdb/explore.do?structureId=4HIQ) | The Structure of V122I Mutant Transthyretin in Complex with AG10 | [23716704](https://www.ncbi.nlm.nih.gov/pubmed/23716704) | 2012 | 3-[3-(3,5-dimethyl-1H-pyrazol-4-yl)propoxy]-4-fluorobenzoic acid |
| [4HIS](http://www.rcsb.org/pdb/explore.do?structureId=4HIS) | The Structure of V122I Mutant Transthyretin in Complex with Tafamidis | [23716704](https://www.ncbi.nlm.nih.gov/pubmed/23716704) | 2012 | 2-(3,5-dichlorophenyl)-1,3-benzoxazole-6-carboxylic acid |
| [4QYN](http://www.rcsb.org/pdb/explore.do?structureId=4QYN) | The Crystal Structures of holo-wt human Cellular Retinol Binding protein II (hCRBPII) bound to Retinol | [25478840](https://www.ncbi.nlm.nih.gov/pubmed/25478840) | 2014 | ACETATE ION / RETINOL |
| [4QYP](http://www.rcsb.org/pdb/explore.do?structureId=4QYP) | The Crystal Structures of holo-wt human Cellular Retinol Binding protein II (hCRBPII) bound to Retinal | [25478840](https://www.ncbi.nlm.nih.gov/pubmed/25478840) | 2014 | ACETATE ION / RETINAL |
| [4QZT](http://www.rcsb.org/pdb/explore.do?structureId=4QZT) | Crystal Structure of wild type Human Cellular Retinol Binding Protein II (hCRBPII) bound to retinol at 7 KeV beam energy | [25478840](https://www.ncbi.nlm.nih.gov/pubmed/25478840) | 2014 | ACETATE ION / RETINOL |
| [4QZU](http://www.rcsb.org/pdb/explore.do?structureId=4QZU) | Crystal Structure of wild type Human Cellular Retinol Binding Protein II (hCRBPII) bound to retinol at 11 KeV beam energy | [25478840](https://www.ncbi.nlm.nih.gov/pubmed/25478840) | 2014 | ACETATE ION / GLYCEROL / RETINOL |
| [4RUU](http://www.rcsb.org/pdb/explore.do?structureId=4RUU) | Crystal structure of the Q108K:K40L mutant of human Cellular Retinol Binding ProteinII in complex with All-trans-Retinal after 24 hour incubation at 1.4 Angstrom Resolution | [23224553](https://www.ncbi.nlm.nih.gov/pubmed/23224553) | 2014 | ACETATE ION / RETINAL |
| [4ZCB](http://www.rcsb.org/pdb/explore.do?structureId=4ZCB) | Human CRBPII mutant - Y60W dimer | [27524203](https://www.ncbi.nlm.nih.gov/pubmed/27524203) | 2015 | N/A |
| [4ZGU](http://www.rcsb.org/pdb/explore.do?structureId=4ZGU) | Crystal structure of monomer Y60W hCRBPII | [27524203](https://www.ncbi.nlm.nih.gov/pubmed/27524203) | 2015 | ACETATE ION |
| [4ZH6](http://www.rcsb.org/pdb/explore.do?structureId=4ZH6) | Crystal Structure of the Domain-Swapped Dimer Y60L mutant of Human Cellular Retinol Binding Protein II | [27524203](https://www.ncbi.nlm.nih.gov/pubmed/27524203) | 2015 | ACETATE ION |
| [4ZH9](http://www.rcsb.org/pdb/explore.do?structureId=4ZH9) | Crystal Structure of the Domain-Swapped Dimer Wild-Type of Human Cellular Retinol Binding Protein II | [27524203](https://www.ncbi.nlm.nih.gov/pubmed/27524203) | 2015 | N/A |
| [4ZJ0](http://www.rcsb.org/pdb/explore.do?structureId=4ZJ0) | The crystal structure of monomer Q108K:K40L:Y60W CRBPII bound to all-trans-retinal | [27524203](https://www.ncbi.nlm.nih.gov/pubmed/27524203) | 2015 | ACETATE ION / RETINAL |
| [4ZR2](http://www.rcsb.org/pdb/explore.do?structureId=4ZR2) | Crystal Structure of the Domain-Swapped Dimer K40L:Q108K:Y60W mutant of Human Cellular Retinol Binding Protein II | [27524203](https://www.ncbi.nlm.nih.gov/pubmed/27524203) | 2015 | ACETATE ION / RETINAL |
| [5DG4](http://www.rcsb.org/pdb/explore.do?structureId=5DG4) | Crystal structure of monomer human cellular retinol binding protein II-Y60L | [27524203](https://www.ncbi.nlm.nih.gov/pubmed/27524203) | 2015 | ACETATE ION |
| [5DPQ](http://www.rcsb.org/pdb/explore.do?structureId=5DPQ) | Crystal Structure of E72A mutant of domain swapped dimer Human Cellular Retinol Binding Protein | [27524203](https://www.ncbi.nlm.nih.gov/pubmed/27524203) | 2015 | ACETATE ION |
| [5F58](http://www.rcsb.org/pdb/explore.do?structureId=5F58) | Crystal structure of the Q108K:K40L:T51V:R58F mutant of human Cellular Retinol Binding Protein II in complex with All-trans-Retinal after 24 hours of incubation at 1.54 Angstrom Resolution | N/A | 2015 | ACETATE ION / RETINAL |
| [5F6B](http://www.rcsb.org/pdb/explore.do?structureId=5F6B) | Crystal structure of the Q108K:K40L:T51V:R58Y:Y19W mutant of human Cellular Retinol Binding Protein II in complex with All-trans-Retinal at 1.3 Angstrom Resolution | N/A | 2015 | ACETATE ION / RETINAL |
| [5F7G](http://www.rcsb.org/pdb/explore.do?structureId=5F7G) | Crystal structure of the Q108K:K40L:T51V:R58Y:Y19W:Q38L mutant of human Cellular Retinol Binding Protein II in complex with All-trans-Retinal at 1.48 Angstrom Resolution | N/A | 2015 | ACETATE ION / RETINAL |
| [5FAZ](http://www.rcsb.org/pdb/explore.do?structureId=5FAZ) | Crystal structure of the Q108K:K40L:T51V mutant of human Cellular Retinol Binding Protein II in complex with All-trans-Retinal after 24 hours of incubation at 1.4 Angstrom Resolution | N/A | 2015 | ACETATE ION / RETINAL |
| [5FEN](http://www.rcsb.org/pdb/explore.do?structureId=5FEN) | Crystal structure of the Q108K:K40L:T53C mutant of human Cellular Retinol Binding Protein II in complex with All-trans-Retinal after 24 hours of incubation at 1.55 Angstrom Resolution | N/A | 2015 | ACETATE ION / RETINAL |
| [5FFH](http://www.rcsb.org/pdb/explore.do?structureId=5FFH) | Crystal structure of the Q108K:K40L:T51V:R58W:Y19W mutant of human Cellular Retinol Binding Protein II in complex with All-trans-Retinal at 1.68 Angstrom Resolution | N/A | 2015 | ACETATE ION / RETINAL |
| [5H8T](http://www.rcsb.org/pdb/explore.do?structureId=5H8T) | Crystal structure of human cellular retinol binding protein 1 in complex with all-trans-retinol | [26900151](https://www.ncbi.nlm.nih.gov/pubmed/26900151) | 2015 | RETINOL |
| [5H9A](http://www.rcsb.org/pdb/explore.do?structureId=5H9A) | Crystal structure of the Apo form of human cellular retinol binding protein 1 | [26900151](https://www.ncbi.nlm.nih.gov/pubmed/26900151) | 2015 | 2-[BIS-(2-HYDROXY-ETHYL)-AMINO]-2-HYDROXYMETHYL-PROPANE-1,3-DIOL |
| [5HA1](http://www.rcsb.org/pdb/explore.do?structureId=5HA1) | Crystal structure of human cellular retinol binding protein 1 in complex with retinylamine | [26900151](https://www.ncbi.nlm.nih.gov/pubmed/26900151) | 2015 | (2~{E},4~{E},6~{E},8~{E})-3,7-dimethyl-9-(2,6,6-trimethylcyclohexen-1-yl)nona-2,4,6,8-tetraen-1-amine |
| [5HBS](http://www.rcsb.org/pdb/explore.do?structureId=5HBS) | Crystal structure of human cellular retinol binding protein 1 in complex with all-trans-retinol at 0.89 angstrom. | [26900151](https://www.ncbi.nlm.nih.gov/pubmed/26900151) | 2016 | RETINOL |
| [5LJB](http://www.rcsb.org/pdb/explore.do?structureId=5LJB) | Crystal structure of holo human CRBP1 | [28057518](https://www.ncbi.nlm.nih.gov/pubmed/28057518) | 2016 | RETINOL |
| [5LJC](http://www.rcsb.org/pdb/explore.do?structureId=5LJC) | Crystal structure of holo human CRBP1 | [28057518](https://www.ncbi.nlm.nih.gov/pubmed/28057518) | 2016 | RETINOL / SODIUM ION |
| [5LJD](http://www.rcsb.org/pdb/explore.do?structureId=5LJD) | Crystal structure of holo human CRBP1/K40L mutant | [28057518](https://www.ncbi.nlm.nih.gov/pubmed/28057518) | 2016 | RETINOL / SODIUM ION |
| [5LJE](http://www.rcsb.org/pdb/explore.do?structureId=5LJE) | Crystal structure of holo human CRBP1/K40L,Q108L mutant | [28057518](https://www.ncbi.nlm.nih.gov/pubmed/28057518) | 2016 | RETINOL / SODIUM ION |
| [5LJG](http://www.rcsb.org/pdb/explore.do?structureId=5LJG) | Crystal structure of holo human CRBP1 | [28057518](https://www.ncbi.nlm.nih.gov/pubmed/28057518) | 2016 | PALMITIC ACID |
| [5LJH](http://www.rcsb.org/pdb/explore.do?structureId=5LJH) | Crystal structure of human apo CRBP1/K40L mutant | [28057518](https://www.ncbi.nlm.nih.gov/pubmed/28057518) | 2016 | SODIUM ION |
| [5LJK](http://www.rcsb.org/pdb/explore.do?structureId=5LJK) | Crystal structure of human apo CRBP1 | [28057518](https://www.ncbi.nlm.nih.gov/pubmed/28057518) | 2016 | SODIUM ION |
| [5U6G](http://www.rcsb.org/pdb/explore.do?structureId=5U6G) | Crystal Structure of the holo Domain-Swapped Dimer mutant Q108K:K40D Human Cellular Retinol Binding Protein II bound with all trans retinal | N/A | 2016 | RETINAL |
| [6AT8](http://www.rcsb.org/pdb/explore.do?structureId=6AT8) | 1.1 Angstrom Resolution Structure of Human Cellular Retinol-Binding Protein IV | N/A | 2017 | DI(HYDROXYETHYL)ETHER / GLYCEROL |
| [6BTH](http://www.rcsb.org/pdb/explore.do?structureId=6BTH) | Crystal structure of human cellular retinol binding protein 2 (CRBP2) in complex with 2-arachidonoylglycerol (2-AG) | N/A | 2017 | 1,3-dihydroxypropan-2-yl (5Z,8Z,11Z,14Z)-icosa-5,8,11,14-tetraenoate / DI(HYDROXYETHYL)ETHER |
| [6BTI](http://www.rcsb.org/pdb/explore.do?structureId=6BTI) | Crystal structure of human cellular retinol binding protein 2 (CRBP2) in complex with N-arachidonoylethanolamine (AEA) | N/A | 2017 | (5Z,8Z,11Z,14Z)-N-(2-hydroxyethyl)icosa-5,8,11,14-tetraenamide / DI(HYDROXYETHYL)ETHER |
| [6C7Z](http://www.rcsb.org/pdb/explore.do?structureId=6C7Z) | Crystal structure of the Q108K:K40L:T51V:R58F mutant of human Cellular Retinol Binding Protein II in complex with synthetic Ligand Julolidine | [29645331](https://www.ncbi.nlm.nih.gov/pubmed/29645331) | 2018 | (2E,4E)-3-methyl-5-(2,3,6,7-tetrahydro-1H,5H-pyrido[3,2,1-ij]quinolin-9-yl)penta-2,4-dienal / ACETATE ION |
| [6E50](http://www.rcsb.org/pdb/explore.do?structureId=%226E50%22) | Crystal structure of the apo domain-swapped dimer Q108K:K40L:T51F mutant of human Cellular Retinol Binding Protein II | [31557439](https://www.ncbi.nlm.nih.gov/pubmed/31557439) | 2018 | ACETATE ION |
| [6E51](http://www.rcsb.org/pdb/explore.do?structureId=6E51) | Crystal structure of the apo domain-swapped dimer Q108K:K40L:T51W mutant of human cellular retinol binding protein II | [31557439](https://www.ncbi.nlm.nih.gov/pubmed/31557439) | 2018 | N/A |
| [6E5E](http://www.rcsb.org/pdb/explore.do?structureId=6E5E) | Crystal structure of the apo domain-swapped dimer Q108K:T51D mutant of human cellular retinol binding protein II | [31557439](https://www.ncbi.nlm.nih.gov/pubmed/31557439) | 2018 | ACETATE ION |
| [6E5L](http://www.rcsb.org/pdb/explore.do?structureId=6E5L) | Crystal structure of human cellular retinol binding protein 1 in complex with abnormal-cannabidiol (abn-CBD) | [30721022](https://www.ncbi.nlm.nih.gov/pubmed/30721022) | 2018 | (1'R,2'R)-5'-methyl-6-pentyl-2'-(prop-1-en-2-yl)-1',2',3',4'-tetrahydro[1,1'-biphenyl]-2,4-diol |
| [6E5Q](http://www.rcsb.org/pdb/explore.do?structureId=6E5Q) | Crystal structure of the apo domain-swapped dimer Q108K:T51D:A28H mutant of human Cellular Retinol Binding Protein II | [31557439](https://www.ncbi.nlm.nih.gov/pubmed/31557439) | 2018 | ACETATE ION |
| [6E5R](http://www.rcsb.org/pdb/explore.do?structureId=6E5R) | Crystal structure of the apo domain-swapped dimer Q108K:T51D:A28C mutant of human Cellular Retinol Binding Protein II | [31557439](https://www.ncbi.nlm.nih.gov/pubmed/31557439) | 2018 | ACETATE ION / GLYCEROL |
| [6E5S](http://www.rcsb.org/pdb/explore.do?structureId=6E5S) | Crystal structure of holo retinal-bound domain-swapped dimer Q108K:T51D mutant of human Cellular Retinol Binding Protein II | [31557439](https://www.ncbi.nlm.nih.gov/pubmed/31557439) | 2018 | RETINAL |
| [6E5T](http://www.rcsb.org/pdb/explore.do?structureId=6E5T) | Crystal structure of human cellular retinol binding protein 1 in complex with abnormal-cannabidiorcin (Abn-CBDO) | [30721022](https://www.ncbi.nlm.nih.gov/pubmed/30721022) | 2018 | (1'R,2'R)-5',6-dimethyl-2'-(prop-1-en-2-yl)-1',2',3',4'-tetrahydro[1,1'-biphenyl]-2,4-diol |
| [6E5W](http://www.rcsb.org/pdb/explore.do?structureId=6E5W) | Crystal structure of human cellular retinol binding protein 3 in complex with abnormal-cannabidiol (abn-CBD) | [30721022](https://www.ncbi.nlm.nih.gov/pubmed/30721022) | 2018 | (1'R,2'R)-5'-methyl-6-pentyl-2'-(prop-1-en-2-yl)-1',2',3',4'-tetrahydro[1,1'-biphenyl]-2,4-diol / GLYCEROL |
| [6E6K](http://www.rcsb.org/pdb/explore.do?structureId=6E6K) | Crystal structure of human cellular retinol-binding protein 4 in complex with abnormal-cannabidiol (abn-CBD) | [30721022](https://www.ncbi.nlm.nih.gov/pubmed/30721022) | 2018 | (1'R,2'R)-5'-methyl-6-pentyl-2'-(prop-1-en-2-yl)-1',2',3',4'-tetrahydro[1,1'-biphenyl]-2,4-diol |
| [6E6L](http://www.rcsb.org/pdb/explore.do?structureId=6E6L) | Crystal structure of the holo retinal-bound domain-swapped dimer Q108K:K40L:T51F:Y60A mutant of human cellular retinol binding protein II | [31557439](https://www.ncbi.nlm.nih.gov/pubmed/31557439) | 2018 | ACETATE ION / RETINAL |
| [6E6M](http://www.rcsb.org/pdb/explore.do?structureId=6E6M) | Crystal structure of human cellular retinol-binding protein 1 in complex with cannabidiorcin (CBDO) | [30721022](https://www.ncbi.nlm.nih.gov/pubmed/30721022) | 2018 | (1'R,2'R)-4,5'-dimethyl-2'-(prop-1-en-2-yl)-1',2',3',4'-tetrahydro[1,1'-biphenyl]-2,6-diol |
| [6E7M](http://www.rcsb.org/pdb/explore.do?structureId=6E7M) | Crystal structure of the holo retinal-bound domain-swapped dimer Q108K:T51D:A28C mutant of human Cellular Retinol Binding Protein II | [31557439](https://www.ncbi.nlm.nih.gov/pubmed/31557439) | 2018 | ACETATE ION / GLYCEROL / RETINAL |
| [6MCU](http://www.rcsb.org/pdb/explore.do?structureId=6MCU) | Crystal structure of the holo retinal-bound domain-swapped dimer Q108K:T51D:A28H mutant of human Cellular Retinol Binding Protein II | [31557439](https://www.ncbi.nlm.nih.gov/pubmed/31557439) | 2018 | GLYCEROL / RETINAL |
| [6MCV](http://www.rcsb.org/pdb/explore.do?structureId=6MCV) | Crystal Structure of Holo Retinal-Bound Domain-Swapped Dimer of Wild Type Human Cellular Retinol Binding Protein II | [31557439](https://www.ncbi.nlm.nih.gov/pubmed/31557439) | 2018 | RETINAL |
| [6MKV](http://www.rcsb.org/pdb/explore.do?structureId=6MKV) | Crystal structure of Retinal-bound holo Q108K:K40L:T51W domain-swapped dimer of human cellular retinol binding protein 2 | [31557439](https://www.ncbi.nlm.nih.gov/pubmed/31557439) | 2018 | ACETATE ION / GLYCEROL / RETINAL |
| [6MLB](http://www.rcsb.org/pdb/explore.do?structureId=6MLB) | Crystal structure of the holo retinal-bound domain-swapped dimer Q108K:K40L:T51F mutant of human cellular retinol binding protein II | [31557439](https://www.ncbi.nlm.nih.gov/pubmed/31557439) | 2018 | ACETATE ION / GLYCEROL / RETINAL |
| [6ON5](http://www.rcsb.org/pdb/explore.do?structureId=6ON5) | Crystal Structure of the Zn-bound Domain-Swapped Dimer Q108K:T51D:A28C:L36C:F57H Mutant of Human Cellular Retinol Binding Protein II | [31557439](https://www.ncbi.nlm.nih.gov/pubmed/31557439) | 2019 | ZINC ION |
| [6ON7](http://www.rcsb.org/pdb/explore.do?structureId=6ON7) | Crystal Structure of Apo Domain-Swapped Dimer Q108K:T51D:A28C:L36C Mutant of Human Cellular Retinol Binding Protein II | [31557439](https://www.ncbi.nlm.nih.gov/pubmed/31557439) | 2019 | N/A |
| [6ON8](http://www.rcsb.org/pdb/explore.do?structureId=6ON8) | Crystal Structure of the Reduced Form of Apo Domain-Swapped Dimer Q108K:T51D:A28C:L36C:F57H Mutant of Human Cellular Retinol Binding Protein II | [31557439](https://www.ncbi.nlm.nih.gov/pubmed/31557439) | 2019 | N/A |

### Table S5. Summary of Protein Data Bank results for Cellular Retinoic Acid Binding Proteins (CRABP1 and CRABP2)

| PDB Link | Structure Title | PMID | Year Dep | Ligands Used |
| --- | --- | --- | --- | --- |
| [1CBI](http://www.rcsb.org/pdb/explore.do?structureId=1CBI) | APO-CELLULAR RETINOIC ACID BINDING PROTEIN I | [7563063](https://www.ncbi.nlm.nih.gov/pubmed/7563063) | 1995 | N/A |
| [1CBQ](http://www.rcsb.org/pdb/explore.do?structureId=1CBQ) | CRYSTAL STRUCTURE OF CELLULAR RETINOIC-ACID-BINDING PROTEINS I AND II IN COMPLEX WITH ALL-TRANS-RETINOIC ACID AND A SYNTHETIC RETINOID | [7704533](https://www.ncbi.nlm.nih.gov/pubmed/7704533) | 1994 | 6-(2,3,4,5,6,7-HEXAHYDRO-2,4,4-TRIMETHYL-1-METYLENEINDEN-2-YL)-3-METHYLHEXA-2,4-DIENOIC ACID / PHOSPHATE ION |
| [1CBR](http://www.rcsb.org/pdb/explore.do?structureId=1CBR) | CRYSTAL STRUCTURE OF CELLULAR RETINOIC-ACID-BINDING PROTEINS I AND II IN COMPLEX WITH ALL-TRANS-RETINOIC ACID AND A SYNTHETIC RETINOID | [7704533](https://www.ncbi.nlm.nih.gov/pubmed/7704533) | 1994 | RETINOIC ACID |
| [1CBS](http://www.rcsb.org/pdb/explore.do?structureId=1CBS) | CRYSTAL STRUCTURE OF CELLULAR RETINOIC-ACID-BINDING PROTEINS I AND II IN COMPLEX WITH ALL-TRANS-RETINOIC ACID AND A SYNTHETIC RETINOID | [7704533](https://www.ncbi.nlm.nih.gov/pubmed/7704533) | 1994 | RETINOIC ACID |
| [1XCA](http://www.rcsb.org/pdb/explore.do?structureId=1XCA) | APO-CELLULAR RETINOIC ACID BINDING PROTEIN II | [9600845](https://www.ncbi.nlm.nih.gov/pubmed/9600845) | 1996 | N/A |
| [1BLR](http://www.rcsb.org/pdb/explore.do?structureId=1BLR) | NMR SOLUTION STRUCTURE OF HUMAN CELLULAR RETINOIC ACID BINDING PROTEIN-TYPE II, 22 STRUCTURES | [9737849](https://www.ncbi.nlm.nih.gov/pubmed/9737849) | 1998 | N/A |
| [1BM5](http://www.rcsb.org/pdb/explore.do?structureId=1BM5) | THE SOLUTION STRUCTURE OF A SITE-DIRECTED MUTANT (R111M) OF HUMAN CELLULAR RETIONIC ACID BINDING PROTEIN-TYPE II, NMR, 31 STRUCTURES | [9737883](https://www.ncbi.nlm.nih.gov/pubmed/9737883) | 1998 | N/A |
| [2CBR](http://www.rcsb.org/pdb/explore.do?structureId=2CBR) | CELLULAR RETINOIC ACID BINDING PROTEIN I IN COMPLEX WITH A RETINOBENZOIC ACID (AM80) | [10531482](https://www.ncbi.nlm.nih.gov/pubmed/10531482) | 1999 | 4-[(5,5,8,8-tetramethyl-5,6,7,8-tetrahydronaphthalen-2-yl)carbamoyl]benzoic acid |
| [2CBS](http://www.rcsb.org/pdb/explore.do?structureId=2CBS) | CELLULAR RETINOIC ACID BINDING PROTEIN II IN COMPLEX WITH A SYNTHETIC RETINOIC ACID (RO-13 6307) | [10531482](https://www.ncbi.nlm.nih.gov/pubmed/10531482) | 1999 | 3-METHYL-7-(5,5,8,8-TETRAMETHYL-5,6,7,8-TETRAHYDRO-NAPHTHALEN-2-YL) -OCTA-2,4,6-TRIENOIC ACID |
| [3CBS](http://www.rcsb.org/pdb/explore.do?structureId=3CBS) | CELLULAR RETINOIC ACID BINDING PROTEIN II IN COMPLEX WITH A SYNTHETIC RETINOIC ACID (RO-12 7310) | [10531482](https://www.ncbi.nlm.nih.gov/pubmed/10531482) | 1999 | (2E,4E,6E,8E)-9-(4-hydroxy-2,3,6-trimethylphenyl)-3,7-dimethylnona-2,4,6,8-tetraenoic acid |
| [2FR3](http://www.rcsb.org/pdb/explore.do?structureId=2FR3) | Crystal Structure of Cellular Retinoic Acid Binding Protein Type II in Complex with All-Trans-Retinoic Acid at 1.48 Angstroms Resolution | [16979656](https://www.ncbi.nlm.nih.gov/pubmed/16979656) | 2006 | ACETATE ION / RETINOIC ACID |
| [2FRS](http://www.rcsb.org/pdb/explore.do?structureId=2FRS) | Crystal structure of the f15w mutant of apo-cellular retinoic acid binding protein type ii at 1.51 angstroms resolution | [16979656](https://www.ncbi.nlm.nih.gov/pubmed/16979656) | 2006 | SODIUM ION / |
| [2FS6](http://www.rcsb.org/pdb/explore.do?structureId=2FS6) | Crystal Structure of Apo-Cellular Retinoic Acid Binding Protein Type II At 1.35 Angstroms Resolution | [16979656](https://www.ncbi.nlm.nih.gov/pubmed/16979656) | 2006 | ACETATE ION / CHLORIDE ION / SODIUM ION |
| [2FS7](http://www.rcsb.org/pdb/explore.do?structureId=2FS7) | Crystal Structure of Apo-Cellular Retinoic Acid Binding Protein Type II At 1.55 Angstroms Resolution | [16979656](https://www.ncbi.nlm.nih.gov/pubmed/16979656) | 2006 | ACETATE ION / CHLORIDE ION |
| [2G78](http://www.rcsb.org/pdb/explore.do?structureId=2G78) | Crystal Structure of the R132K:Y134F Mutant of Cellular Retinoic Acid Binding Protein Type II in Complex with All-Trans-Retinoic Acid at 1.70 Angstroms Resolution | [17447762](https://www.ncbi.nlm.nih.gov/pubmed/17447762) | 2006 | RETINOIC ACID / SODIUM ION |
| [2G79](http://www.rcsb.org/pdb/explore.do?structureId=2G79) | Crystal Structure of the R132K:Y134F Mutant of Cellular Retinoic Acid Binding Protein Type II in Complex with All-Trans-Retinal at 1.69 Angstroms Resolution | [17447762](https://www.ncbi.nlm.nih.gov/pubmed/17447762) | 2006 | RETINAL / SODIUM ION / SULFATE ION |
| [2G7B](http://www.rcsb.org/pdb/explore.do?structureId=2G7B) | Crystal Structure of the R132K:R111L:L121E mutant of Cellular Retinoic Acid Binding Protein Type II In Complex With All-Trans-Retinal At 1.18 Angstroms Resolution | [17447762](https://www.ncbi.nlm.nih.gov/pubmed/17447762) | 2006 | ALL-TRANS AXEROPHTHENE / SODIUM ION |
| [3CWK](http://www.rcsb.org/pdb/explore.do?structureId=3CWK) | Crystal Structure of the R132K:Y134F:R111L:T54V:L121E Mutant of Cellular Retinoic Acid Binding Protein Type II in Complex with All-trans-Retinoic Acid at 1.57 Angstroms Resolution | [19018099](https://www.ncbi.nlm.nih.gov/pubmed/19018099) | 2008 | RETINOIC ACID / SULFATE ION |
| [3D95](http://www.rcsb.org/pdb/explore.do?structureId=3D95) | Crystal Structure of the R132K:Y134F:R111L:L121E:T54V Mutant of Apo-Cellular Retinoic Acid Binding Protein Type II at 1.20 Angstroms Resolution | [19018099](https://www.ncbi.nlm.nih.gov/pubmed/19018099) | 2008 | N/A |
| [3D96](http://www.rcsb.org/pdb/explore.do?structureId=3D96) | Crystal Structure of the R132K:Y134F Mutant of Apo-Cellular Retinoic Acid Binding Protein Type II at 1.71 Angstroms Resolution | [19018099](https://www.ncbi.nlm.nih.gov/pubmed/19018099) | 2008 | ACETATE ION / |
| [3I17](http://www.rcsb.org/pdb/explore.do?structureId=3I17) | Crystal structure of the apo R132K:L121E mutant of cellular retinoic acid-binding protein II at 1.68 angstrom resolution | [19603486](https://www.ncbi.nlm.nih.gov/pubmed/19603486) | 2009 | N/A |
| [4I9R](http://www.rcsb.org/pdb/explore.do?structureId=4I9R) [4I9S](http://www.rcsb.org/pdb/explore.do?structureId=4I9S)  [4M6S](http://www.rcsb.org/pdb/explore.do?structureId=4M6S) [4M7M](http://www.rcsb.org/pdb/explore.do?structureId=4M7M) | Crystal Structure of a Mutant of the Cellular Retinoic Acid Binding Protein Type II in Complex with All-Trans Retinal at various conditions | [24059243](https://www.ncbi.nlm.nih.gov/pubmed/24059243) | 2012 | RETINAL |
| [3FEP](http://www.rcsb.org/pdb/explore.do?structureId=3FEP) [4QGV](http://www.rcsb.org/pdb/explore.do?structureId=4QGV)  [4QGW](http://www.rcsb.org/pdb/explore.do?structureId=4QGW) [4QGX](http://www.rcsb.org/pdb/explore.do?structureId=4QGX) | Crystal structure of a CRABPII mutant complexed with a synthetic ligand (merocyanin) at various conditions | [25534273](https://www.ncbi.nlm.nih.gov/pubmed/25534273) | 2008 | (2E,4E,6E)-3-methyl-6-(1,3,3-trimethyl-1,3-dihydro-2H-indol-2-ylidene)hexa-2,4-dienal / 2-(N-MORPHOLINO)-ETHANESULFONIC ACID |
| [5HZQ](http://www.rcsb.org/pdb/explore.do?structureId=5HZQ) | Crystal structure of cellular retinoic acid binding protein 2 (CRABP2)-aryl fluorosulfate covalent conjugate | [27191344](https://www.ncbi.nlm.nih.gov/pubmed/27191344) | 2016 | 4'-[(3,6,9,12-tetraoxapentadec-14-yn-1-yl)oxy][1,1'-biphenyl]-4-yl sulfurofluoridate / GLYCEROL |
| \| [4YBP](http://www.rcsb.org/pdb/explore.do?structureId=4YBP) \| [4YFR](http://www.rcsb.org/pdb/explore.do?structureId=4YFR) \| \| --- \| --- \| \| [4YBU](http://www.rcsb.org/pdb/explore.do?structureId=4YBU) \| [4YGG](http://www.rcsb.org/pdb/explore.do?structureId=4YGG) \| \| [4YCE](http://www.rcsb.org/pdb/explore.do?structureId=4YCE) \| [4YGH](http://www.rcsb.org/pdb/explore.do?structureId=4YGH) \| \| [4YCH](http://www.rcsb.org/pdb/explore.do?structureId=4YCH) \| [4YGZ](http://www.rcsb.org/pdb/explore.do?structureId=4YGZ) \| \| [4YDA](http://www.rcsb.org/pdb/explore.do?structureId=4YDA) \| [4YH0](http://www.rcsb.org/pdb/explore.do?structureId=4YH0) \| \| [4YDB](http://www.rcsb.org/pdb/explore.do?structureId=4YDB) \| [4YKM](http://www.rcsb.org/pdb/explore.do?structureId=4YKM) \| \| [4YFP](http://www.rcsb.org/pdb/explore.do?structureId=4YFP) \| [4YKO](http://www.rcsb.org/pdb/explore.do?structureId=4YKO) \| \| [4YFQ](http://www.rcsb.org/pdb/explore.do?structureId=4YFQ) \| | Crystal structure of the mutant of human cellular retinoic acid binding protein II with retinal – various conditions | [27310917](https://www.ncbi.nlm.nih.gov/pubmed/27310917) | 2015 | RETINAL |
| \| [6MOP](http://www.rcsb.org/pdb/explore.do?structureId=6MOP) \| [6MQI](http://www.rcsb.org/pdb/explore.do?structureId=6MQI) \| \| --- \| --- \| \| [6MOQ](http://www.rcsb.org/pdb/explore.do?structureId=6MOQ) \| [6MQJ](http://www.rcsb.org/pdb/explore.do?structureId=6MQJ) \| \| [6MOR](http://www.rcsb.org/pdb/explore.do?structureId=6MOR) \| [6MQW](http://www.rcsb.org/pdb/explore.do?structureId=6MQW) \| \| [6MOV](http://www.rcsb.org/pdb/explore.do?structureId=6MOV) \| [6MQX](http://www.rcsb.org/pdb/explore.do?structureId=6MQX) \| \| [6MOX](http://www.rcsb.org/pdb/explore.do?structureId=6MOX) \| [6MQY](http://www.rcsb.org/pdb/explore.do?structureId=6MQY) \| \| [6MPK](http://www.rcsb.org/pdb/explore.do?structureId=6MPK) \| [6MQZ](http://www.rcsb.org/pdb/explore.do?structureId=6MQZ) \| \| [6MR0](http://www.rcsb.org/pdb/explore.do?structureId=6MR0) \| | Crystal Structure of the All-trans Retinal-Bound Mutant of Human Cellular Retinoic Acid Binding Protein II – various conditions | [30580520](https://www.ncbi.nlm.nih.gov/pubmed/30580520) | 2018 | RETINAL |
| [6HKR](http://www.rcsb.org/pdb/explore.do?structureId=6HKR) | Human Cellular Retinoic Acid Binding Protein II (CRABPII) with bound synthetic retinoid DC271. | [30613343](https://www.ncbi.nlm.nih.gov/pubmed/30613343) | 2018 | 1,2-ETHANEDIOL / 4-[2-(4,4-dimethyl-1-propan-2-yl-2,3-dihydroquinolin-6-yl)ethynyl]benzoic acid / GLYCEROL / TETRAETHYLENE GLYCOL |
| [5OGB](http://www.rcsb.org/pdb/explore.do?structureId=5OGB) | Human Cellular Retinoic Acid Binding Protein II (CRABPII) with bound synthetic retinoid DC360. | [30707838](https://www.ncbi.nlm.nih.gov/pubmed/30707838) | 2017 | 4-[2-(4,4-dimethyl-1-propan-2-yl-quinolin-6-yl)ethynyl]benzoic acid |
| [3CR6](http://www.rcsb.org/pdb/explore.do?structureId=3CR6) | Crystal Structure of the R132K:R111L:A32E Mutant of Cellular Retinoic Acid Binding Protein Type II Complexed with C15-aldehyde (a retinal analog) at 1.22 Angstrom resolution. | N/A | 2008 | 1,3,3-trimethyl-2-[(1E,3E)-3-methylpenta-1,3-dien-1-yl]cyclohexene |
| [3D97](http://www.rcsb.org/pdb/explore.do?structureId=3D97) | Crystal Structure of the R132K:R111L:L121E Mutant of Apo-Cellular Retinoic Acid Binding Protein Type II At 1.50 Angstroms Resolution | N/A | 2008 | 2-[3-(2-HYDROXY-1,1-DIHYDROXYMETHYL-ETHYLAMINO)-PROPYLAMINO]-2-HYDROXYMETHYL-PROPANE-1,3-DIOL / SODIUM ION |
| [3F8A](http://www.rcsb.org/pdb/explore.do?structureId=3F8A) | Crystal Structure of the R132K:R111L:L121E:R59W Mutant of Cellular Retinoic Acid-Binding Protein Type II Complexed with C15-aldehyde (a retinal analog) at 1.95 Angstrom resolution. | N/A | 2008 | 1,3,3-trimethyl-2-[(1E,3E)-3-methylpenta-1,3-dien-1-yl]cyclohexene / 2-[3-(2-HYDROXY-1,1-DIHYDROXYMETHYL-ETHYLAMINO)-PROPYLAMINO]-2-HYDROXYMETHYL-PROPANE-1,3-DIOL |
| [3F9D](http://www.rcsb.org/pdb/explore.do?structureId=3F9D) | Crystal structure of the R132K:R111L:T54E mutant of cellular retinoic acid-binding protein II complexed with C15-aldehyde (a retinal analog) at 2.00 angstrom resolution | N/A | 2008 | 1,3,3-trimethyl-2-[(1E,3E)-3-methylpenta-1,3-dien-1-yl]cyclohexene |
| [3FA6](http://www.rcsb.org/pdb/explore.do?structureId=3FA6) | Crystal structure of the R132K:Y134F:R111L:L121D:T54V mutant of cellular retinoic acid-binding protein II complexed with C15-aldehyde (a retinal analog) at 1.54 angstrom resolution | N/A | 2008 | 1,3,3-trimethyl-2-[(1E,3E)-3-methylpenta-1,3-dien-1-yl]cyclohexene |
| [3FA7](http://www.rcsb.org/pdb/explore.do?structureId=3FA7) | Crystal structure of the apo R132K:R111L:L121E:R59E mutant of cellular retinoic acid-binding protein II at 1.90 angstrom resolution | N/A | 2008 | 2-[3-(2-HYDROXY-1,1-DIHYDROXYMETHYL-ETHYLAMINO)-PROPYLAMINO]-2-HYDROXYMETHYL-PROPANE-1,3-DIOL |
| [3FA8](http://www.rcsb.org/pdb/explore.do?structureId=3FA8) | Crystal structure of the apo R132K:Y134F:R111L:L121E mutant of cellular retinoic acid-binding protein II at 1.78 angstrom resolution | N/A | 2008 | N/A |
| [3FA9](http://www.rcsb.org/pdb/explore.do?structureId=3FA9) | Crystal structure of the apo R132K:Y134F:R111L:L121D mutant of cellular retinoic acid-binding protein II at 1.94 angstrom resolution | N/A | 2008 | ACETATE ION / |
| [3FEK](http://www.rcsb.org/pdb/explore.do?structureId=3FEK) | Crystal structure of the R132K:Y134F:R111L:L121D:T54V mutant of cellular retinoic acid-binding protein II at 1.51 angstrom resolution | N/A | 2008 | ACETATE ION / DI(HYDROXYETHYL)ETHER / |
| [3FEL](http://www.rcsb.org/pdb/explore.do?structureId=3FEL) | Crystal structure of the R132K:R111L:T54E mutant of cellular retinoic acid-binding protein II at 1.85 angstrom resolution | N/A | 2008 | 2-[3-(2-HYDROXY-1,1-DIHYDROXYMETHYL-ETHYLAMINO)-PROPYLAMINO]-2-HYDROXYMETHYL-PROPANE-1,3-DIOL |
| [3FEN](http://www.rcsb.org/pdb/explore.do?structureId=3FEN) | Crystal structure of the R132K:R111L:A32E mutant of cellular retinoic acid-binding protein II at 1.56 angstrom resolution | N/A | 2008 | CHLORIDE ION |
| [6AT8](http://www.rcsb.org/pdb/explore.do?structureId=6AT8) | 1.1 Angstrom Resolution Structure of Human Cellular Retinol-Binding Protein IV | N/A | 2017 | DI(HYDROXYETHYL)ETHER / GLYCEROL |
| [6MOW](http://www.rcsb.org/pdb/explore.do?structureId=6MOW) | Crystal Structure of the apo R111K:Y134F:T54V:R132Q:P39Y:R59Y:L121Q mutant of Human Cellular Retinoic Acid Binding Protein II at 2.3 Angstrom Resolution | N/A | 2018 | N/A |
| [6NNX](http://www.rcsb.org/pdb/explore.do?structureId=6NNX) | Crystal Structure of the All-Trans Retinal-Bound R111K:Y134F:T54V:R132Q:P39Y:R59Y:L121M Mutant of Human Cellular Retinoic Acid Binding Protein II in the Dark at 1.87 Angstrom Resolution | N/A | 2019 | RETINAL |
| [6NNY](http://www.rcsb.org/pdb/explore.do?structureId=6NNY) | Crystal Structure of the All-Trans Retinal-Bound R111K:Y134F:T54V:R132Q:P39E:R59Y:L121E Mutant of Human Cellular Retinoic Acid Binding Protein II in the Dark at 1.67 Angstrom Resolution | N/A | 2019 | RETINAL |
| [6NOE](http://www.rcsb.org/pdb/explore.do?structureId=6NOE) | Crystal Structure of the All-Trans Retinal-Bound R111K:Y134F:T54V:R132Q:P39Y:R59Y:L121E:I63D Mutant of Human Cellular Retinoic Acid Binding Protein II in the Dark at 1.97 Angstrom Resolution | N/A | 2019 | RETINAL |

### Table S6. Summary of ChEMBL results for Cellular Retinoic Acid Binding Proteins (CRABP1 and CRABP2)

| Assay ID | Chem Ct | Assay Description | PMID |
| --- | --- | --- | --- |
| [CHEMBL661352](https://www.ebi.ac.uk/chembl/assay/inspect/CHEMBL661352) | 10 | Inhibition of chick skin Cytoplasmic retinoic acid binding protein at 100-fold excess ligand | [8809153](https://www.ncbi.nlm.nih.gov/pubmed/8809153) |
| [CHEMBL661351](https://www.ebi.ac.uk/chembl/assay/inspect/CHEMBL661351) | 6 | Tested for its ability to inhibit the binding of (all-E-)-RA to cytoplasmic retinoic acid-binding protein (CRABP) from chick skin | [7799400](https://www.ncbi.nlm.nih.gov/pubmed/7799400) |
| [CHEMBL884347](https://www.ebi.ac.uk/chembl/assay/inspect/CHEMBL884347) | 1 | Inhibition of [3H]ATRA binding to chick skin Cytoplasmic retinoic acid binding protein | [8809153](https://www.ncbi.nlm.nih.gov/pubmed/8809153) |
| [CHEMBL665219](https://www.ebi.ac.uk/chembl/assay/inspect/CHEMBL665219) | 8 | Inhibition of [3H]ATRA binding to chick skin Cytoplasmic retinoic acid binding protein | [8809153](https://www.ncbi.nlm.nih.gov/pubmed/8809153) |
| [CHEMBL665218](https://www.ebi.ac.uk/chembl/assay/inspect/CHEMBL665218) | 7 | Inhibition of binding to chick skin Cytoplasmic retinoic acid binding protein | [7608895](https://www.ncbi.nlm.nih.gov/pubmed/7608895) |
| [CHEMBL662931](https://www.ebi.ac.uk/chembl/assay/inspect/CHEMBL662931) | 1 | Concentration of compound required to inhibit binding of 2.5 uM [3H]all-trans-retinoic acid by 50% in chick | [2738885](https://www.ncbi.nlm.nih.gov/pubmed/2738885) |
| [CHEMBL662930](https://www.ebi.ac.uk/chembl/assay/inspect/CHEMBL662930) | 1 | Concentration of compound required to inhibit binding of 2.5 uM [3H]all-trans-retinoic acid by 50% in chick | [2738885](https://www.ncbi.nlm.nih.gov/pubmed/2738885) |
| [CHEMBL662929](https://www.ebi.ac.uk/chembl/assay/inspect/CHEMBL662929) | 7 | Concentration of compound required to inhibit binding of 2.5 uM [3H]all-trans-retinoic acid by 50% in chick | [2738885](https://www.ncbi.nlm.nih.gov/pubmed/2738885) |
| [CHEMBL665905](https://www.ebi.ac.uk/chembl/assay/inspect/CHEMBL665905) | 7 | Binding affinity for mouse Cytoplasmic retinoic acid binding protein type 2 | [7608895](https://www.ncbi.nlm.nih.gov/pubmed/7608895) |
| [CHEMBL875705](https://www.ebi.ac.uk/chembl/assay/inspect/CHEMBL875705) | 7 | Inhibition of [3H]ATRA binding to murine Cytoplasmic retinoic acid binding protein (CRABP) type 2 | [8809153](https://www.ncbi.nlm.nih.gov/pubmed/8809153) |
| [CHEMBL665904](https://www.ebi.ac.uk/chembl/assay/inspect/CHEMBL665904) | 4 | Inhibition of [3H]ATRA binding to murine Cytoplasmic retinoic acid binding protein (CRABP) type 2 | [8809153](https://www.ncbi.nlm.nih.gov/pubmed/8809153) |
| [CHEMBL665903](https://www.ebi.ac.uk/chembl/assay/inspect/CHEMBL665903) | 7 | Binding affinity for mouse Cytoplasmic retinoic acid binding protein type 1 | [7608895](https://www.ncbi.nlm.nih.gov/pubmed/7608895) |
| [CHEMBL665902](https://www.ebi.ac.uk/chembl/assay/inspect/CHEMBL665902) | 9 | Inhibition of [3H]ATRA binding to murine Cytoplasmic retinoic acid binding protein (CRABP) type 1 | [8809153](https://www.ncbi.nlm.nih.gov/pubmed/8809153) |
| [CHEMBL665901](https://www.ebi.ac.uk/chembl/assay/inspect/CHEMBL665901) | 1 | Inhibition of [3H]ATRA binding to murine Cytoplasmic retinoic acid binding protein (CRABP) type 1 | [8809153](https://www.ncbi.nlm.nih.gov/pubmed/8809153) |
| [CHEMBL2182124](https://www.ebi.ac.uk/chembl/assay/inspect/CHEMBL2182124) | 1 | Binding affinity to Flag-tagged cIAP1/CRABP2 expressed in human MCF7 cells assessed as reduction in CRABP2 level at 0.1 uM to 1 uM after 6 hrs by Western blot analysis | [21515062](https://www.ncbi.nlm.nih.gov/pubmed/21515062) |
| [CHEMBL2182123](https://www.ebi.ac.uk/chembl/assay/inspect/CHEMBL2182123) | 1 | Binding affinity to Flag-tagged cIAP1/CRABP2 expressed in human HT1080 cells assessed as reduction in CRABP2 level at 0.1 uM to 1 uM after 6 hrs by Western blot analysis | [21515062](https://www.ncbi.nlm.nih.gov/pubmed/21515062) |
| [CHEMBL2182119](https://www.ebi.ac.uk/chembl/assay/inspect/CHEMBL2182119) | 1 | Binding affinity to Flag-tagged cIAP1/CRABP2 expressed in human IMR32 cells assessed as reduction in CRABP-2 level at 1 to 10 uM after 48 hrs by Western blot analysis | [21515062](https://www.ncbi.nlm.nih.gov/pubmed/21515062) |
| [CHEMBL2182118](https://www.ebi.ac.uk/chembl/assay/inspect/CHEMBL2182118) | 1 | Binding affinity to Flag-tagged cIAP1/CRABP2 expressed in human IMR32 cells assessed as reduction in MycN level at 1 to 10 uM after 48 hrs by Western blot analysis | [21515062](https://www.ncbi.nlm.nih.gov/pubmed/21515062) |
| [CHEMBL2188719](https://www.ebi.ac.uk/chembl/assay/inspect/CHEMBL2188719) | 1 | Binding affinity to Flag-tagged cIAP1/CRABP2 expressed in human HT1080 cells assessed as induction in CRABP-2 degradation at 1000 uM by Western blot analysis | [21515062](https://www.ncbi.nlm.nih.gov/pubmed/21515062) |
| [CHEMBL2188718](https://www.ebi.ac.uk/chembl/assay/inspect/CHEMBL2188718) | 1 | Binding affinity to Flag-tagged cIAP1/CRABP2 expressed in human HT1080 cells assessed as induction in CRABP-2 degradation at 1 uM after 6 hrs by Western blot analysis in presence of MeBS | [21515062](https://www.ncbi.nlm.nih.gov/pubmed/21515062) |
| [CHEMBL2188717](https://www.ebi.ac.uk/chembl/assay/inspect/CHEMBL2188717) | 1 | Binding affinity to Flag-tagged cIAP1/CRABP2 expressed in human HT1080 cells assessed as induction in CRABP-2 degradation at 1 uM after 6 hrs by Western blot analysis | [21515062](https://www.ncbi.nlm.nih.gov/pubmed/21515062) |
| [CHEMBL2188716](https://www.ebi.ac.uk/chembl/assay/inspect/CHEMBL2188716) | 1 | Binding affinity to Flag-tagged cIAP1/CRABP2 expressed in human HT1080 cells assessed as induction in CRABP-2 degradation at 1 uM after 6 hrs by Western blot analysis in presence of proteasome inhibitor MG132 | [21515062](https://www.ncbi.nlm.nih.gov/pubmed/21515062) |
| [CHEMBL2188715](https://www.ebi.ac.uk/chembl/assay/inspect/CHEMBL2188715) | 2 | Binding affinity to Flag-tagged cIAP1/CRABP2 expressed in human HT1080 cells assessed as formation of ternary complex by GST-pull down assay | [21515062](https://www.ncbi.nlm.nih.gov/pubmed/21515062) |
| [CHEMBL2188714](https://www.ebi.ac.uk/chembl/assay/inspect/CHEMBL2188714) | 1 | Binding affinity to Flag-tagged cIAP1/CRABP2 expressed in human HT1080 cells assessed as induction in CRABP-2 degradation at 1 uM after 6 hrs by Western blot analysis in presence of proteasome inhibitor lactacystin | [21515062](https://www.ncbi.nlm.nih.gov/pubmed/21515062) |
| [CHEMBL2188713](https://www.ebi.ac.uk/chembl/assay/inspect/CHEMBL2188713) | 1 | Binding affinity to Flag-tagged cIAP1/CRABP2 expressed in human HT1080 cells assessed as induction in cIAP1 degradation at 30 uM after 6 hrs by Western blot analysis | [21515062](https://www.ncbi.nlm.nih.gov/pubmed/21515062) |
| [CHEMBL2188712](https://www.ebi.ac.uk/chembl/assay/inspect/CHEMBL2188712) | 1 | Binding affinity to Flag-tagged cIAP1/CRABP2 expressed in human HT1080 cells assessed as induction in CRABP-2 degradation after 6 hrs by Western blot analysis | [21515062](https://www.ncbi.nlm.nih.gov/pubmed/21515062) |
| [CHEMBL2188711](https://www.ebi.ac.uk/chembl/assay/inspect/CHEMBL2188711) | 2 | Binding affinity to Flag-tagged cIAP1/CRABP2 expressed in human HT1080 cells assessed as induction in cIAP1 degradation at 0.1 to 10 uM after 6 hrs by Western blot analysis | [21515062](https://www.ncbi.nlm.nih.gov/pubmed/21515062) |
| [CHEMBL2188710](https://www.ebi.ac.uk/chembl/assay/inspect/CHEMBL2188710) | 1 | Binding affinity to Flag-tagged cIAP1/CRABP2 expressed in human HT1080 cells assessed as induction in CRABP-2 degradation at 0.1 to 10 uM after 6 hrs by Western blot analysis | [21515062](https://www.ncbi.nlm.nih.gov/pubmed/21515062) |
| [CHEMBL2188708](https://www.ebi.ac.uk/chembl/assay/inspect/CHEMBL2188708) | 1 | Binding affinity to Flag-tagged cIAP1/CRABP2 expressed in human HT1080 cells assessed as induction in CRABP-2 degradation after 12 hrs by Western blot analysis | [21515062](https://www.ncbi.nlm.nih.gov/pubmed/21515062) |
| [CHEMBL2188707](https://www.ebi.ac.uk/chembl/assay/inspect/CHEMBL2188707) | 1 | Binding affinity to Flag-tagged cIAP1/CRABP2 expressed in human HT1080 cells assessed as induction in CRABP-2 degradation after 48 hrs by Western blot analysis | [21515062](https://www.ncbi.nlm.nih.gov/pubmed/21515062) |
| [CHEMBL664300](https://www.ebi.ac.uk/chembl/assay/inspect/CHEMBL664300) | 2 | Inhibition of [3H]-retinoic acid binding to Cellular retinoic acid-binding protein (CRABP) from chick embryo skin | [12723955](https://www.ncbi.nlm.nih.gov/pubmed/12723955) |
| [CHEMBL664299](https://www.ebi.ac.uk/chembl/assay/inspect/CHEMBL664299) | 11 | Inhibition of [3H]-retinoic acid binding to Cellular retinoic acid-binding protein (CRABP) from chick embryo skin | [12723955](https://www.ncbi.nlm.nih.gov/pubmed/12723955) |
| [CHEMBL664298](https://www.ebi.ac.uk/chembl/assay/inspect/CHEMBL664298) | 1 | Inhibition of [3H]-retinoic acid binding to Cellular retinoic acid-binding protein (CRABP) from chick embryo skin | [12723955](https://www.ncbi.nlm.nih.gov/pubmed/12723955) |

### Table S7. Summary of ChEMBL entries for CYP26

| Assay ID | Chem Ct | Assay Description | PMID |
| --- | --- | --- | --- |
|  |  |  |  |
| [CHEMBL3789273](https://www.ebi.ac.uk/chembl/assay/inspect/CHEMBL3789273) | 2 | Inhibition of CYP26A1-mediated atRA metabolism in human HepG2 cells assessed as atRA level in cell media at 1 uM after 24 hrs by LC/MS-MS analysis (Rvb = 0.49 +/- 0.33 nM) | [26918322](https://www.ncbi.nlm.nih.gov/pubmed/26918322) |
| [CHEMBL3789272](https://www.ebi.ac.uk/chembl/assay/inspect/CHEMBL3789272) | 3 | Inhibition of CYP26A1-mediated atRA metabolism in human HepG2 cells assessed as atRA level in cell media at 1 uM after 24 hrs in presence of 100 nM atRA by LC/MS-MS analysis relative to atRA-treated control | [26918322](https://www.ncbi.nlm.nih.gov/pubmed/26918322) |
| [CHEMBL3789264](https://www.ebi.ac.uk/chembl/assay/inspect/CHEMBL3789264) | 24 | Inhibition of microsomal fraction of human CYP26A1 expressed in Sf9 cells using 9-cis-RA as substrate preincubated for 5 mins followed by NADPH addition measured after 1 min by HPLC analysis in presence of rat P450 reductase | [26918322](https://www.ncbi.nlm.nih.gov/pubmed/26918322) |
| [CHEMBL3789258](https://www.ebi.ac.uk/chembl/assay/inspect/CHEMBL3789258) | 3 | Inhibition of CYP26A1 in human HepG2 cells assessed as upregulation of RARbeta mRNA expression at 1 uM after 24 hrs in presence of 100 nM atRA by RT-PCR method | [26918322](https://www.ncbi.nlm.nih.gov/pubmed/26918322) |
| [CHEMBL3789257](https://www.ebi.ac.uk/chembl/assay/inspect/CHEMBL3789257) | 3 | Inhibition of CYP26A1 in human HepG2 cells assessed as upregulation of CYP26A1 mRNA expression at 1 uM after 24 hrs in presence of 100 nM atRA by RT-PCR method | [26918322](https://www.ncbi.nlm.nih.gov/pubmed/26918322) |
| [CHEMBL3789256](https://www.ebi.ac.uk/chembl/assay/inspect/CHEMBL3789256) | 3 | Inhibition of CYP26A1-mediated atRA metabolism in human HepG2 cells assessed as atRA level in cell media at 1 uM after 24 hrs in presence of 100 nM atRA by LC/MS-MS analysis (Rvb = 13.2 +/- 0.14 nM) | [26918322](https://www.ncbi.nlm.nih.gov/pubmed/26918322) |
| [CHEMBL3620667](https://www.ebi.ac.uk/chembl/assay/inspect/CHEMBL3620667) | 2 | Inhibition of CYP26A1-mediated ATRA metabolism in human HL60 cells assessed as ATRA level at 5 uM incubated for 96 hrs by HPLC method (Rvb = 1.21 uM) | [26365710](https://www.ncbi.nlm.nih.gov/pubmed/26365710) |
| [CHEMBL3620666](https://www.ebi.ac.uk/chembl/assay/inspect/CHEMBL3620666) | 2 | Inhibition of CYP26A1-mediated ATRA metabolism in human HL60 cells assessed as ATRA level at 5 uM incubated for 72 hrs by HPLC method (Rvb = 3.6 uM) | [26365710](https://www.ncbi.nlm.nih.gov/pubmed/26365710) |
| [CHEMBL3620665](https://www.ebi.ac.uk/chembl/assay/inspect/CHEMBL3620665) | 2 | Inhibition of CYP26A1-mediated ATRA metabolism in human HL60 cells assessed as ATRA level at 5 uM incubated for 48 hrs by HPLC method (Rvb = 4.95 uM) | [26365710](https://www.ncbi.nlm.nih.gov/pubmed/26365710) |
| [CHEMBL3620664](https://www.ebi.ac.uk/chembl/assay/inspect/CHEMBL3620664) | 2 | Inhibition of CYP26A1-mediated ATRA metabolism in human HL60 cells assessed as ATRA level at 5 uM incubated for 24 hrs by HPLC method (Rvb = 7.19 uM) | [26365710](https://www.ncbi.nlm.nih.gov/pubmed/26365710) |
| [CHEMBL3620653](https://www.ebi.ac.uk/chembl/assay/inspect/CHEMBL3620653) | 16 | Inhibition of CYP26A1 in ATRA-induced human HL60 cell microsomes incubated for 30 mins in dark condition with NADPH and ATRA by HPLC method | [26365710](https://www.ncbi.nlm.nih.gov/pubmed/26365710) |
| [CHEMBL3620652](https://www.ebi.ac.uk/chembl/assay/inspect/CHEMBL3620652) | 4 | Inhibition of CYP26A1 (unknown origin) | [26365710](https://www.ncbi.nlm.nih.gov/pubmed/26365710) |
| [CHEMBL3399274](https://www.ebi.ac.uk/chembl/assay/inspect/CHEMBL3399274) | 22 | Inhibition of CYP26A1 in ATRA-induced human HL60 cell microsomes incubated for 30 mins using ATRA and NADPH by HPLC method | [25684424](https://www.ncbi.nlm.nih.gov/pubmed/25684424) |
| [CHEMBL2417110](https://www.ebi.ac.uk/chembl/assay/inspect/CHEMBL2417110) | 2 | Inhibition of CYP26A1 in human NB4 cells assessed as ATRA level in medium at 5 uM after 12 hrs by LC-MS/MS analysis (Rvb = 27.05 +/- 1.34 ng/ml) | [23601821](https://www.ncbi.nlm.nih.gov/pubmed/23601821) |
| [CHEMBL2214483](https://www.ebi.ac.uk/chembl/assay/inspect/CHEMBL2214483) | 9 | Inhibition of CYP26A1 in human MCF7 cell microsomes using [3H]ATRA as substrate after 1 hr by scintillation counter analysis | [22727372](https://www.ncbi.nlm.nih.gov/pubmed/22727372) |
| [CHEMBL2148657](https://www.ebi.ac.uk/chembl/assay/inspect/CHEMBL2148657) | 16 | Inhibition of CYP26A1-mediated retinoic acid metabolism in human MCF7 cell microsomes using [3H]ATRA as substrate after 1 hr by scintillation counting | [22989911](https://www.ncbi.nlm.nih.gov/pubmed/22989911) |
| [CHEMBL1833546](https://www.ebi.ac.uk/chembl/assay/inspect/CHEMBL1833546) | 3 | Inhibition of CYP26 in human liver microsomes | [21838328](https://www.ncbi.nlm.nih.gov/pubmed/21838328) |
| [CHEMBL1833279](https://www.ebi.ac.uk/chembl/assay/inspect/CHEMBL1833279) | 18 | Inhibition of human CYP26A1 assessed using [11,12-3H]ATRA as substrate by scintillation counting | [21838328](https://www.ncbi.nlm.nih.gov/pubmed/21838328) |
| [CHEMBL1768149](https://www.ebi.ac.uk/chembl/assay/inspect/CHEMBL1768149) | 18 | Inhibition of CYP26A1 in human MCF7 cell microsomes using [3H]ATRA after 1 hr by scintillation counting | [21428449](https://www.ncbi.nlm.nih.gov/pubmed/21428449) |
| [CHEMBL996935](https://www.ebi.ac.uk/chembl/assay/inspect/CHEMBL996935) | 15 | Inhibition of CYP26A1 in human MCF7 cells | [18722776](https://www.ncbi.nlm.nih.gov/pubmed/18722776) |
| [CHEMBL926263](https://www.ebi.ac.uk/chembl/assay/inspect/CHEMBL926263) | 1 | Inhibition of CYP26 | [17544277](https://www.ncbi.nlm.nih.gov/pubmed/17544277) |
| [CHEMBL916381](https://www.ebi.ac.uk/chembl/assay/inspect/CHEMBL916381) | 1 | Inhibition of ATRA-induced CYP26 in human T47D cells assessed as ATRA metabolism using [11.12-3H]-ATRA up to 10 uM | [15615521](https://www.ncbi.nlm.nih.gov/pubmed/15615521) |
| [CHEMBL916380](https://www.ebi.ac.uk/chembl/assay/inspect/CHEMBL916380) | 1 | Inhibition of ATRA-induced CYP26 in human MCF7 cells assessed as ATRA metabolism using [11.12-3H]-ATRA up to 10 uM | [15615521](https://www.ncbi.nlm.nih.gov/pubmed/15615521) |
| [CHEMBL916379](https://www.ebi.ac.uk/chembl/assay/inspect/CHEMBL916379) | 19 | Inhibition of ATRA-induced CYP26 in human T47D cell microsome assessed as ATRA metabolism using [11.12-3H]-ATRA | [15615521](https://www.ncbi.nlm.nih.gov/pubmed/15615521) |
| [CHEMBL916378](https://www.ebi.ac.uk/chembl/assay/inspect/CHEMBL916378) | 18 | Inhibition of ATRA-induced CYP26 in human T47D cells assessed as ATRA metabolism using [11.12-3H]-ATRA | [15615521](https://www.ncbi.nlm.nih.gov/pubmed/15615521) |
| [CHEMBL916377](https://www.ebi.ac.uk/chembl/assay/inspect/CHEMBL916377) | 18 | Inhibition of ATRA-induced CYP26 in human MCF7 cells assessed ATRA as metabolism using [11.12-3H]-ATRA | [15615521](https://www.ncbi.nlm.nih.gov/pubmed/15615521) |
| [CHEMBL853200](https://www.ebi.ac.uk/chembl/assay/inspect/CHEMBL853200) | 20 | Inhibition of CYP26 expressed in human T47D cell line | [16504501](https://www.ncbi.nlm.nih.gov/pubmed/16504501) |
| [CHEMBL865876](https://www.ebi.ac.uk/chembl/assay/inspect/CHEMBL865876) | 6 | Activity against CYP26 up to 20 uM | [16451067](https://www.ncbi.nlm.nih.gov/pubmed/16451067) |
| [CHEMBL832851](https://www.ebi.ac.uk/chembl/assay/inspect/CHEMBL832851) | 31 | Potency towards cytochrome P 450 26 enzyme activity | [15745819](https://www.ncbi.nlm.nih.gov/pubmed/15745819) |
| [CHEMBL3789263](https://www.ebi.ac.uk/chembl/assay/inspect/CHEMBL3789263) | 24 | Inhibition of microsomal fraction of human CYP26B1 expressed in Sf9 cells using 9-cis-RA as substrate preincubated for 5 mins followed by NADPH addition measured after 5 mins by HPLC analysis in presence of rat P450 reductase | [26918322](https://www.ncbi.nlm.nih.gov/pubmed/26918322) |
| [CHEMBL3789262](https://www.ebi.ac.uk/chembl/assay/inspect/CHEMBL3789262) | 1 | Inhibition of microsomal fraction of human CYP26B1 expressed in Sf9 cells using 9-cis-RA as substrate at 20 uM preincubated for 5 mins followed by NADPH addition measured after 5 mins by HPLC analysis in presence of rat P450 reductase | [26918322](https://www.ncbi.nlm.nih.gov/pubmed/26918322) |

### Table S8. Summary of ChEMBL records returned by a search on “retinol dehydrogenase”

| Assay ID | Chem Ct | Assay description | PMID |
| --- | --- | --- | --- |
| [CHEMBL4260594](https://www.ebi.ac.uk/chembl/assay/inspect/CHEMBL4260594) | 1 | Inhibition of ADH1B (unknown origin) assessed as reduction in NADH production by spectrophotometric analysis | [30340896](https://www.ncbi.nlm.nih.gov/pubmed/30340896) |
| [CHEMBL641134](https://www.ebi.ac.uk/chembl/assay/inspect/CHEMBL641134) | 23 | Inhibition of human alcohol dehydrogenase beta 1 activity | [9572895](https://www.ncbi.nlm.nih.gov/pubmed/9572895) |
| [CHEMBL641140](https://www.ebi.ac.uk/chembl/assay/inspect/CHEMBL641140) | 1 | Inhibitory activity against human alcohol dehydrogenase sigma. | [9572895](https://www.ncbi.nlm.nih.gov/pubmed/9572895) |
| [CHEMBL641139](https://www.ebi.ac.uk/chembl/assay/inspect/CHEMBL641139) | 3 | Inhibitory activity against human alcohol dehydrogenase sigma; ND=not determined | [9572895](https://www.ncbi.nlm.nih.gov/pubmed/9572895) |
| [CHEMBL641138](https://www.ebi.ac.uk/chembl/assay/inspect/CHEMBL641138) | 19 | Inhibition of human alcohol dehydrogenase sigma activity | [9572895](https://www.ncbi.nlm.nih.gov/pubmed/9572895) |
| [CHEMBL641137](https://www.ebi.ac.uk/chembl/assay/inspect/CHEMBL641137) | 4 | Inhibitory activity against human alcohol dehydrogenase pi; ND=not determined | [9572895](https://www.ncbi.nlm.nih.gov/pubmed/9572895) |
| [CHEMBL641136](https://www.ebi.ac.uk/chembl/assay/inspect/CHEMBL641136) | 19 | Inhibition of human alcohol dehydrogenase pi activity | [9572895](https://www.ncbi.nlm.nih.gov/pubmed/9572895) |
| [CHEMBL1031472](https://www.ebi.ac.uk/chembl/assay/inspect/CHEMBL1031472) | 8 | Inhibition of 3alpha-HSD | [19188072](https://www.ncbi.nlm.nih.gov/pubmed/19188072) |
| [CHEMBL892201](https://www.ebi.ac.uk/chembl/assay/inspect/CHEMBL892201) | 3 | Inhibition of 3alphaHSD | [17346963](https://www.ncbi.nlm.nih.gov/pubmed/17346963) |
| [CHEMBL898185](https://www.ebi.ac.uk/chembl/assay/inspect/CHEMBL898185) | 2 | Inhibition of rat liver 3-alpha-HSD assessed as 5-beta-dihydrocortisone reduction at 500 uM | [17517512](https://www.ncbi.nlm.nih.gov/pubmed/17517512) |
| [CHEMBL898184](https://www.ebi.ac.uk/chembl/assay/inspect/CHEMBL898184) | 2 | Inhibition of rat liver 3alphaHSD assessed as 5-beta-dihydrocortisone reduction at 250 uM | [17517512](https://www.ncbi.nlm.nih.gov/pubmed/17517512) |
| [CHEMBL898183](https://www.ebi.ac.uk/chembl/assay/inspect/CHEMBL898183) | 21 | Inhibition of rat liver 3-alpha-HSD assessed as 5-beta-dihydrocortisone reduction | [17517512](https://www.ncbi.nlm.nih.gov/pubmed/17517512) |
| [CHEMBL898182](https://www.ebi.ac.uk/chembl/assay/inspect/CHEMBL898182) | 17 | Inhibition of rat liver 3alphaHSD assessed as 5-beta-dihydrocortisone reduction at 1 mM | [17517512](https://www.ncbi.nlm.nih.gov/pubmed/17517512) |
| [CHEMBL3282425](https://www.ebi.ac.uk/chembl/assay/inspect/CHEMBL3282425) | 2 | Binding affinity to alcohol dehydrogenase (unknown origin) | [1246029](https://www.ncbi.nlm.nih.gov/pubmed/1246029) |
| [CHEMBL3257323](https://www.ebi.ac.uk/chembl/assay/inspect/CHEMBL3257323) | 5 | Inhibition of alcohol dehydrogenase (unknown origin) assessed as dissociation constant for the complex of enzyme and DPNH | [321782](https://www.ncbi.nlm.nih.gov/pubmed/321782) |
| [CHEMBL822089](https://www.ebi.ac.uk/chembl/assay/inspect/CHEMBL822089) | 1 | The compound was tested for the ability to inactivate human liver alcohol dehydrogenase in the presence of 1 mM NAD+k | [7009869](https://www.ncbi.nlm.nih.gov/pubmed/7009869) |
| [CHEMBL822088](https://www.ebi.ac.uk/chembl/assay/inspect/CHEMBL822088) | 1 | The compound was tested for the ability to inactivate human liver alcohol dehydrogenase in the absence of NAD+K | [7009869](https://www.ncbi.nlm.nih.gov/pubmed/7009869) |
| [CHEMBL645349](https://www.ebi.ac.uk/chembl/assay/inspect/CHEMBL645349) | 7 | The compound was tested for the ability to inactivate human liver alcohol dehydrogenase in the presence of 1 mM NAD+k | [7009869](https://www.ncbi.nlm.nih.gov/pubmed/7009869) |
| [CHEMBL875754](https://www.ebi.ac.uk/chembl/assay/inspect/CHEMBL875754) | 1 | The compound was tested for the ability to inactivate human liver alcohol dehydrogenase in the presence of 1 mM NAD+K | [7009869](https://www.ncbi.nlm.nih.gov/pubmed/7009869) |
| [CHEMBL645348](https://www.ebi.ac.uk/chembl/assay/inspect/CHEMBL645348) | 1 | The compound was tested for the ability to inactivate human liver alcohol dehydrogenase in the presence of 1 mM NAD+(K | [7009869](https://www.ncbi.nlm.nih.gov/pubmed/7009869) |
| [CHEMBL645347](https://www.ebi.ac.uk/chembl/assay/inspect/CHEMBL645347) | 10 | The compound was tested for the ability to inactivate human liver alcohol dehydrogenase in the absence of NAD+K | [7009869](https://www.ncbi.nlm.nih.gov/pubmed/7009869) |
| [CHEMBL645346](https://www.ebi.ac.uk/chembl/assay/inspect/CHEMBL645346) | 1 | The compound was tested for the ability to inactivate human liver alcohol dehydrogenase in the absence of NAD+(K | [7009869](https://www.ncbi.nlm.nih.gov/pubmed/7009869) |
| [CHEMBL876583](https://www.ebi.ac.uk/chembl/assay/inspect/CHEMBL876583) | 1 | Michaelis-Menten's constant was determined in vitro against purified rat liver alcohol dehydrogenase in presence of ethanol | [6343601](https://www.ncbi.nlm.nih.gov/pubmed/6343601) |
| [CHEMBL645231](https://www.ebi.ac.uk/chembl/assay/inspect/CHEMBL645231) | 5 | In vitro inhibitory constant against monkey liver alcohol dehydrogenase was determined | [3155552](https://www.ncbi.nlm.nih.gov/pubmed/3155552) |

### Table S9. Summary of Protein Data Bank records for Aldehyde Dehydrogenase (ALDH1A1, ALDH1A2, ALDH1A3)

| PDB ID | Structure Title | PMID | Year  Dep | Ligands Used |
| --- | --- | --- | --- | --- |
| [4WB9](http://www.rcsb.org/pdb/explore.do?structureId=4WB9) | Human ALDH1A1 complexed with NADH | [25450233](https://www.ncbi.nlm.nih.gov/pubmed/25450233) | 2014 | 1,4-DIHYDRONICOTINAMIDE ADENINE DINUCLEOTIDE / CHLORIDE ION / YTTERBIUM (III) ION |
| [4WJ9](http://www.rcsb.org/pdb/explore.do?structureId=4WJ9) | Structure of Human apo ALDH1A1 | [25450233](https://www.ncbi.nlm.nih.gov/pubmed/25450233) | 2014 | CHLORIDE ION / YTTERBIUM (III) ION |
| [4WP7](http://www.rcsb.org/pdb/explore.do?structureId=4WP7) | Structure of human ALDH1A1 with inhibitor CM026 | [25634381](https://www.ncbi.nlm.nih.gov/pubmed/25634381) | 2014 | 8-{[4-(furan-2-ylcarbonyl)piperazin-1-yl]methyl}-1,3-dimethyl-7-(3-methylbutyl)-3,7-dihydro-1H-purine-2,6-dione / CHLORIDE ION / YTTERBIUM (III) ION |
| [4WPN](http://www.rcsb.org/pdb/explore.do?structureId=4WPN) | Structure of human ALDH1A1 with inhibitor CM053 | [25634381](https://www.ncbi.nlm.nih.gov/pubmed/25634381) | 2014 | 1-{[1,3-dimethyl-7-(3-methylbutyl)-2,6-dioxo-2,3,6,7-tetrahydro-1H-purin-8-yl]methyl}piperidine-4-carboxamide / CHLORIDE ION / YTTERBIUM (III) ION |
| [4X4L](http://www.rcsb.org/pdb/explore.do?structureId=4X4L) | Structure of human ALDH1A1 with inhibitor CM037 | [25634381](https://www.ncbi.nlm.nih.gov/pubmed/25634381) | 2014 | 1,4-DIHYDRONICOTINAMIDE ADENINE DINUCLEOTIDE / CHLORIDE ION / ethyl ({4-oxo-3-[3-(pyrrolidin-1-yl)propyl]-3,4-dihydro[1]benzothieno[3,2-d]pyrimidin-2-yl}sulfanyl)acetate / SODIUM ION / YTTERBIUM (III) ION |
| [5FHZ](http://www.rcsb.org/pdb/explore.do?structureId=5FHZ) | Human aldehyde dehydrogenase 1A3 complexed with NAD(+) and retinoic acid | [27759097](https://www.ncbi.nlm.nih.gov/pubmed/27759097) | 2015 | NICOTINAMIDE-ADENINE-DINUCLEOTIDE / RETINOIC ACID |
| [5L2M](http://www.rcsb.org/pdb/explore.do?structureId=5L2M) | Structure of ALDH1A1 in complex with BUC11 | [28219011](https://www.ncbi.nlm.nih.gov/pubmed/28219011) | 2016 | 2,3,5-trimethyl-6-[3-oxo-3-(piperidin-1-yl)propyl]-7H-furo[3,2-g][1]benzopyran-7-one / CHLORIDE ION / S-HYDROXYCYSTEINE / YTTERBIUM (III) ION |
| [5L2N](http://www.rcsb.org/pdb/explore.do?structureId=5L2N) | Structure of ALDH1A1 in complex with BUC25 | [28219011](https://www.ncbi.nlm.nih.gov/pubmed/28219011) | 2016 | 3-benzyl-4-methyl-2-oxo-2H-1-benzopyran-7-yl methanesulfonate / CHLORIDE ION / S-HYDROXYCYSTEINE / YTTERBIUM (III) ION |
| [5L2O](http://www.rcsb.org/pdb/explore.do?structureId=5L2O) | Crystal Structure of ALDH1A1 in complex with BUC22 | [28219011](https://www.ncbi.nlm.nih.gov/pubmed/28219011) | 2016 | 7-(diethylamino)-4-methyl-2H-1-benzopyran-2-one / CHLORIDE ION / YTTERBIUM (III) ION |
| [5TEI](http://www.rcsb.org/pdb/explore.do?structureId=5TEI) | Structure of human ALDH1A1 with inhibitor CM039 | N/A | 2016 | 1,4-DIHYDRONICOTINAMIDE ADENINE DINUCLEOTIDE / 6-{[(3-fluorophenyl)methyl]sulfanyl}-5-(2-methylphenyl)-2,5-dihydro-4H-pyrazolo[3,4-d]pyrimidin-4-one / CHLORIDE ION / YTTERBIUM (III) ION |
| [6DUM](http://www.rcsb.org/pdb/explore.do?structureId=6DUM) | ALDH1A1 N121S in complex with 6-{[(3-fluorophenyl)methyl]sulfanyl}-2-(oxetan-3-yl)-5-phenyl-2,5-dihydro-4H-pyrazolo[3,4-d]pyrimidin-4-one (compound 13g) | [30221940](https://www.ncbi.nlm.nih.gov/pubmed/30221940) | 2018 | 1,4-DIHYDRONICOTINAMIDE ADENINE DINUCLEOTIDE / 6-{[(3-fluorophenyl)methyl]sulfanyl}-2-(oxetan-3-yl)-5-phenyl-2,5-dihydro-4H-pyrazolo[3,4-d]pyrimidin-4-one / CHLORIDE ION / YTTERBIUM (III) ION |
| [5FHZ](http://www.rcsb.org/pdb/explore.do?structureId=5FHZ) | Human aldehyde dehydrogenase 1A3 complexed with NAD(+) and retinoic acid | [27759097](https://www.ncbi.nlm.nih.gov/pubmed/27759097) | 2015 | NICOTINAMIDE-ADENINE-DINUCLEOTIDE / RETINOIC ACID |
| [4X2Q](http://www.rcsb.org/pdb/explore.do?structureId=4X2Q) | Crystal Structure of Human Aldehyde Dehydrogenase, ALDH1a2 | N/A | 2014 | NICOTINAMIDE-ADENINE-DINUCLEOTIDE |
| [6ALJ](http://www.rcsb.org/pdb/explore.do?structureId=6ALJ) | ALDH1A2 liganded with NAD and compound WIN18,446 | [29240402](https://www.ncbi.nlm.nih.gov/pubmed/29240402) | 2017 | N,N'-(octane-1,8-diyl)bis(2,2-dichloroacetamide)/NICOTINAMIDE-ADENINE-DINUCLEOTIDE |
| [6B5G](http://www.rcsb.org/pdb/explore.do?structureId=6B5G) | ALDH1A2 liganded with NAD and (3-ethoxythiophen-2-yl){4-[4-nitro-3-(pyrrolidin-1-yl)phenyl]piperazin-1-yl}methanone (compound 6-118) | [29240402](https://www.ncbi.nlm.nih.gov/pubmed/29240402) | 2017 | (3-ethoxythiophen-2-yl){4-[4-nitro-3-(pyrrolidin-1-yl)phenyl]piperazin-1-yl}methanone / NICOTINAMIDE-ADENINE-DINUCLEOTIDE |
| [6B5H](http://www.rcsb.org/pdb/explore.do?structureId=6B5H) | ALDH1A2 liganded with NAD and 1-(4-cyanophenyl)-N-(3-fluorophenyl)-3-[4-(methylsulfonyl)phenyl]-1H-pyrazole-4-carboxamide (compound CM121) | [29240402](https://www.ncbi.nlm.nih.gov/pubmed/29240402) | 2017 | 1-(4-cyanophenyl)-N-(3-fluorophenyl)-3-[4-(methylsulfonyl)phenyl]-1H-pyrazole-4-carboxamide / NICOTINAMIDE-ADENINE-DINUCLEOTIDE |
| [6B5I](http://www.rcsb.org/pdb/explore.do?structureId=6B5I) | ALDH1A2 liganded with 1-(4-cyanophenyl)-N-(3-fluorophenyl)-3-[4-(methylsulfonyl)phenyl]-1H-pyrazole-4-carboxamide (compound CM121) | [29240402](https://www.ncbi.nlm.nih.gov/pubmed/29240402) | 2017 | 1-(4-cyanophenyl)-N-(3-fluorophenyl)-3-[4-(methylsulfonyl)phenyl]-1H-pyrazole-4-carboxamide |

### Table S10. Summary of ChEMBL results for Aldehyde Dehydrogenase (ALDH1A1, ALDH1A2, ALDH1A3)

| Assay ID | Chem Ct | Assay Description | PMID |
| --- | --- | --- | --- |
| [CHEMBL4009360](https://www.ebi.ac.uk/chembl/assay/inspect/CHEMBL4009360) | 1 | Non-competitive inhibition of full length recombinant human ALDH1A1 expressed in Escherichia coli BL21(DE3) assessed as reduction in dehydrogenase activity by measuring NAD(P)H level using varying levels acetaldehyde in presence of NAD+ by Lineweaver-Burk plot analysis | [28219011](https://www.ncbi.nlm.nih.gov/pubmed/28219011) |
| [CHEMBL4009355](https://www.ebi.ac.uk/chembl/assay/inspect/CHEMBL4009355) | 2 | Non-competitive inhibition of full length recombinant human ALDH1A1 expressed in Escherichia coli BL21(DE3) assessed as reduction in dehydrogenase activity by measuring NAD(P)H level using varying levels propionaldehyde in presence of NAD+ by Lineweaver-Burk plot analysis | [28219011](https://www.ncbi.nlm.nih.gov/pubmed/28219011) |
| [CHEMBL4009350](https://www.ebi.ac.uk/chembl/assay/inspect/CHEMBL4009350) | 1 | Uncompetitive inhibition of full length recombinant human ALDH1A1 expressed in Escherichia coli BL21(DE3) using propionaldehyde as substrate in presence of varying levels NAD+ by Lineweaver-Burk plot analysis | [28219011](https://www.ncbi.nlm.nih.gov/pubmed/28219011) |
| [CHEMBL4009344](https://www.ebi.ac.uk/chembl/assay/inspect/CHEMBL4009344) | 37 | Inhibition of full length recombinant human ALDH1A1 expressed in Escherichia coli BL21(DE3) assessed as remaining dehydrogenase activity by measuring NAD(P)H level at 10 uM preincubated for 2 mins followed by addition of propionaldehyde as substrate in presence of NAD+ by spectrophotometric method | [28219011](https://www.ncbi.nlm.nih.gov/pubmed/28219011) |
| [CHEMBL4009338](https://www.ebi.ac.uk/chembl/assay/inspect/CHEMBL4009338) | 3 | Inhibition of full length recombinant human ALDH1A1 expressed in Escherichia coli BL21(DE3) assessed as reduction in dehydrogenase activity by measuring NAD(P)H level preincubated for 2 mins followed by addition of propionaldehyde as substrate in presence of NAD+ by spectrophotometric method relative to control | [28219011](https://www.ncbi.nlm.nih.gov/pubmed/28219011) |
| [CHEMBL4009337](https://www.ebi.ac.uk/chembl/assay/inspect/CHEMBL4009337) | 15 | Inhibition of full length recombinant human ALDH1A1 expressed in Escherichia coli BL21(DE3) assessed as reduction in dehydrogenase activity by measuring NAD(P)H level preincubated for 2 mins followed by addition of propionaldehyde as substrate in presence of NAD+ by spectrophotometric method | [28219011](https://www.ncbi.nlm.nih.gov/pubmed/28219011) |
| [CHEMBL3888331](https://www.ebi.ac.uk/chembl/assay/inspect/CHEMBL3888331) | 16 | Inhibition Assay: IC50 values were determined for CB29 and its analogs using propionaldehyde as the substrate for ALDH1A1 and ALDH2 or benzaldehyde as the substrate for ALDH3A1. | N/A |
| [CHEMBL3562125](https://www.ebi.ac.uk/chembl/assay/inspect/CHEMBL3562125) | 231 | PubChem BioAssay. Extended Characterization of HPGD Inhibitors: Counterscreen Against ALDH1A1. (Class of assay: confirmatory) | N/A |
| [CHEMBL3419577](https://www.ebi.ac.uk/chembl/assay/inspect/CHEMBL3419577) | 1 | Non-competitive partial inhibition of recombinant human ALDH1A1 using 800 uM NAD+ as cofactor by Lineweaver-Burk plot analysis in presence of 100 to 800 uM acetaldehyde | [25634381](https://www.ncbi.nlm.nih.gov/pubmed/25634381) |
| [CHEMBL3419576](https://www.ebi.ac.uk/chembl/assay/inspect/CHEMBL3419576) | 20 | Inhibition of recombinant human ALDH1A1 using propionaldehyde as substrate preincubated for 2 mins with NAD+ followed by substrate addition by UV-Vis spectrophotometric analysis | [25634381](https://www.ncbi.nlm.nih.gov/pubmed/25634381) |
| [CHEMBL3418217](https://www.ebi.ac.uk/chembl/assay/inspect/CHEMBL3418217) | 1 | Inhibition of recombinant human ALDH1A1 using propionaldehyde as substrate at 20 uM preincubated for 2 mins with NAD+ followed by substrate addition by UV-Vis spectrophotometric analysis | [25634381](https://www.ncbi.nlm.nih.gov/pubmed/25634381) |
| [CHEMBL3418216](https://www.ebi.ac.uk/chembl/assay/inspect/CHEMBL3418216) | 2 | Inhibition of human ALDH1A1 G458N mutant using propionaldehyde as substrate preincubated for 2 mins with NAD+ followed by substrate addition by UV-Vis spectrophotometric analysis | [25634381](https://www.ncbi.nlm.nih.gov/pubmed/25634381) |
| [CHEMBL3418011](https://www.ebi.ac.uk/chembl/assay/inspect/CHEMBL3418011) | 1 | Inhibition of human ALDH1A1 G458N mutant using propionaldehyde as substrate at 20 uM preincubated for 2 mins with NAD+ followed by substrate addition by UV-Vis spectrophotometric analysis | [25634381](https://www.ncbi.nlm.nih.gov/pubmed/25634381) |
| [CHEMBL3418010](https://www.ebi.ac.uk/chembl/assay/inspect/CHEMBL3418010) | 17 | Inhibition of human ALDH1A1 G458N mutant using propionaldehyde as substrate at 100 uM preincubated for 2 mins with NAD+ followed by substrate addition by UV-Vis spectrophotometric analysis relative to control | [25634381](https://www.ncbi.nlm.nih.gov/pubmed/25634381) |
| [CHEMBL3418009](https://www.ebi.ac.uk/chembl/assay/inspect/CHEMBL3418009) | 1 | Competitive tight inhibition of recombinant human ALDH1A1 using 1000 uM NAD+ as cofactor by Lineweaver-Burk plot analysis in presence of 100 to 800 uM acetaldehyde | [25634381](https://www.ncbi.nlm.nih.gov/pubmed/25634381) |
| [CHEMBL3418008](https://www.ebi.ac.uk/chembl/assay/inspect/CHEMBL3418008) | 1 | Non-competitive tight inhibition of recombinant human ALDH1A1 using 1000 uM NAD+ as cofactor by Lineweaver-Burk plot analysis in presence of 100 to 800 uM acetaldehyde | [25634381](https://www.ncbi.nlm.nih.gov/pubmed/25634381) |
| [CHEMBL3418007](https://www.ebi.ac.uk/chembl/assay/inspect/CHEMBL3418007) | 2 | Effect on recombinant human ALDH1A1 using propionaldehyde as substrate up to 250 uM preincubated for 2 mins with NAD+ followed by substrate addition by UV-Vis spectrophotometric analysis | [25634381](https://www.ncbi.nlm.nih.gov/pubmed/25634381) |
| [CHEMBL3421198](https://www.ebi.ac.uk/chembl/assay/inspect/CHEMBL3421198) | 1 | Uncompetitive partial inhibition of recombinant human ALDH1A1 using 200 uM propionaldehyde as substrate by Lineweaver-Burk plot analysis in presence of 25 to 250 uM NAD+ | [25634381](https://www.ncbi.nlm.nih.gov/pubmed/25634381) |
| [CHEMBL3421186](https://www.ebi.ac.uk/chembl/assay/inspect/CHEMBL3421186) | 5 | Inhibition of recombinant human ALDH1A1 using propionaldehyde as substrate at 100 uM preincubated for 2 mins with NAD+ followed by substrate addition by UV-Vis spectrophotometric analysis relative to control | [25634381](https://www.ncbi.nlm.nih.gov/pubmed/25634381) |
| [CHEMBL3421185](https://www.ebi.ac.uk/chembl/assay/inspect/CHEMBL3421185) | 1 | Competitive inhibition of recombinant human ALDH1A1 using 800 uM NAD+ as cofactor by Lineweaver-Burk plot analysis in presence of 100 to 800 uM acetaldehyde | [25634381](https://www.ncbi.nlm.nih.gov/pubmed/25634381) |
| [CHEMBL3130567](https://www.ebi.ac.uk/chembl/assay/inspect/CHEMBL3130567) | 22 | Inhibition of human ALDH1A1 using propionaldehyde as substrate preincubated for 2 mins followed by substrate addition by spectrophotometry in presence of NAD+ | [24444054](https://www.ncbi.nlm.nih.gov/pubmed/24444054) |
| [CHEMBL3130566](https://www.ebi.ac.uk/chembl/assay/inspect/CHEMBL3130566) | 1 | Noncompetitive/mixed type inhibition of human ALDH1A1 by Lineweaver-Burk plot analysis in presence of NAD+ | [24444054](https://www.ncbi.nlm.nih.gov/pubmed/24444054) |
| [CHEMBL3128579](https://www.ebi.ac.uk/chembl/assay/inspect/CHEMBL3128579) | 1 | Competitive inhibition of human ALDH1A1 using propionaldehyde as substrate by Lineweaver-Burk plot analysis | [24444054](https://www.ncbi.nlm.nih.gov/pubmed/24444054) |
| [CHEMBL3118330](https://www.ebi.ac.uk/chembl/assay/inspect/CHEMBL3118330) | 1 | Inhibition of human ALDH1A1 using propionaldehyde as substrate up to 250 uM preincubated for 1 min followed by substrate addition by spectrophotometric analysis | [24387105](https://www.ncbi.nlm.nih.gov/pubmed/24387105) |
| [CHEMBL3118132](https://www.ebi.ac.uk/chembl/assay/inspect/CHEMBL3118132) | 21 | Inhibition of human ALDH1A1 using propionaldehyde as substrate preincubated for 1 min followed by substrate addition by spectrophotometric analysis | [24387105](https://www.ncbi.nlm.nih.gov/pubmed/24387105) |
| [CHEMBL3118130](https://www.ebi.ac.uk/chembl/assay/inspect/CHEMBL3118130) | 3 | Activation of human ALDH1A1 using propionaldehyde as substrate at 100 uM preincubated for 1 min followed by substrate addition by spectrophotometric analysis | [24387105](https://www.ncbi.nlm.nih.gov/pubmed/24387105) |
| [CHEMBL3107142](https://www.ebi.ac.uk/chembl/assay/inspect/CHEMBL3107142) | 9 | Inhibition of ALDH1A1 (unknown origin) using NAD+/propionaldehyde as substrate after 15 mins by UV-fluorescence assay | [24360556](https://www.ncbi.nlm.nih.gov/pubmed/24360556) |
| [CHEMBL1738585](https://www.ebi.ac.uk/chembl/assay/inspect/CHEMBL1738585) | 87 | PUBCHEM_BIOASSAY: Inhibitors of Aldehyde Dehydrogenase 1 (ALDH1A1): Follow up Confirmation and Counterscreen. (Class of assay: confirmatory) [Related pubchem assays (depositor defined):AID1030, AID2407] | N/A |
| [CHEMBL1614458](https://www.ebi.ac.uk/chembl/assay/inspect/CHEMBL1614458) | 16117 | PUBCHEM_BIOASSAY: qHTS Assay for Inhibitors of Aldehyde Dehydrogenase 1 (ALDH1A1). (Class of assay: confirmatory) [Related pubchem assays: 1030 (qHTS Validation Assay for Inhibitors of aldehyde dehydrogenase 1 (ALDH1A1))] | N/A |
| [CHEMBL1110890](https://www.ebi.ac.uk/chembl/assay/inspect/CHEMBL1110890) | 6 | Inhibition of ALDH1 in human K562 cells at 15 uM after 45 mins by flow cytometry based method relative to bodipy-aminoacetaldehyde | [20222671](https://www.ncbi.nlm.nih.gov/pubmed/20222671) |
| [CHEMBL1019415](https://www.ebi.ac.uk/chembl/assay/inspect/CHEMBL1019415) | 1 | Effect on human recombinant his-tagged cytosolic ALDH1A1 activity expressed in Escherichia coli BL21 cells at 100 uM | [18787169](https://www.ncbi.nlm.nih.gov/pubmed/18787169) |
| [CHEMBL997777](https://www.ebi.ac.uk/chembl/assay/inspect/CHEMBL997777) | 1 | Inhibition of ALDH1 | [17692435](https://www.ncbi.nlm.nih.gov/pubmed/17692435) |
| [CHEMBL997770](https://www.ebi.ac.uk/chembl/assay/inspect/CHEMBL997770) | 2 | Inhibition of ALDH1 in human DU145 cells at 7.5 uM after 3 days | [17692435](https://www.ncbi.nlm.nih.gov/pubmed/17692435) |
| [CHEMBL997769](https://www.ebi.ac.uk/chembl/assay/inspect/CHEMBL997769) | 2 | Inhibition of ALDH1 in human DU145 cells at 5 uM after 3 days | [17692435](https://www.ncbi.nlm.nih.gov/pubmed/17692435) |
| [CHEMBL997768](https://www.ebi.ac.uk/chembl/assay/inspect/CHEMBL997768) | 2 | Inhibition of ALDH1 in human DU145 cells at 2.5 uM after 3 days | [17692435](https://www.ncbi.nlm.nih.gov/pubmed/17692435) |
| [CHEMBL844222](https://www.ebi.ac.uk/chembl/assay/inspect/CHEMBL844222) | 1 | Inhibition of the enzyme Alcohol dehydrogenase was measured in rats which are treated with phenobarbital (PB) | [7392033](https://www.ncbi.nlm.nih.gov/pubmed/7392033) |
| [CHEMBL643983](https://www.ebi.ac.uk/chembl/assay/inspect/CHEMBL643983) | 12 | Inhibitory activity against yeast aldehyde dehydrogenase (AIDH) | [9667978](https://www.ncbi.nlm.nih.gov/pubmed/9667978) |
| [CHEMBL643982](https://www.ebi.ac.uk/chembl/assay/inspect/CHEMBL643982) | 3 | Inhibitory activity against aldehyde dehydrogenase in rats. | [9191964](https://www.ncbi.nlm.nih.gov/pubmed/9191964) |
| [CHEMBL643981](https://www.ebi.ac.uk/chembl/assay/inspect/CHEMBL643981) | 1 | Evaluated for inhibition of hepatic mitochondrial Aldehyde dehydrogenase (ALDH) from rat | [7990120](https://www.ncbi.nlm.nih.gov/pubmed/7990120) |
| [CHEMBL641149](https://www.ebi.ac.uk/chembl/assay/inspect/CHEMBL641149) | 1 | Evaluated for inhibition of class II mitochondrial Aldehyde dehydrogenase (ALDH) in osmotically disrupted mitochondria from rat | [7990120](https://www.ncbi.nlm.nih.gov/pubmed/7990120) |
| [CHEMBL875244](https://www.ebi.ac.uk/chembl/assay/inspect/CHEMBL875244) | 3 | Evaluated in vivo for inhibition of liver mitochondrial aldehyde dehydrogenase activity 2 hour after administering into 5 to 8 rats, and percentage of activity remaining was reported at a concentration of 1.25 mmol/Kg | [6541256](https://www.ncbi.nlm.nih.gov/pubmed/6541256) |
| [CHEMBL641148](https://www.ebi.ac.uk/chembl/assay/inspect/CHEMBL641148) | 2 | Evaluated in vivo for inhibition of liver mitochondrial aldehyde dehydrogenase activity 2 hour after administering into rats, at a dose of 1.25 mmol/kg | [6541256](https://www.ncbi.nlm.nih.gov/pubmed/6541256) |
| [CHEMBL641147](https://www.ebi.ac.uk/chembl/assay/inspect/CHEMBL641147) | 1 | Evaluated in vivo for inhibition of liver mitochondrial aldehyde dehydrogenase activity 2 hour after administering into 5 to 8 rats, at a dose of 1.25 mmol/kg | [6541256](https://www.ncbi.nlm.nih.gov/pubmed/6541256) |
| [CHEMBL641146](https://www.ebi.ac.uk/chembl/assay/inspect/CHEMBL641146) | 7 | Evaluated in vitro for inhibition of mitochondrial aldehyde dehydrogenase activity in intact rat liver mitochondria (1.0 mM) | [6541256](https://www.ncbi.nlm.nih.gov/pubmed/6541256) |
| [CHEMBL641145](https://www.ebi.ac.uk/chembl/assay/inspect/CHEMBL641145) | 4 | Evaluated in vitro for inhibition of mitochondrial aldehyde dehydrogenase activity in disrupted rat liver mitochondria (1.0 mM) | [6541256](https://www.ncbi.nlm.nih.gov/pubmed/6541256) |
| [CHEMBL641144](https://www.ebi.ac.uk/chembl/assay/inspect/CHEMBL641144) | 9 | Evaluated in vitro for inhibition of mitochondrial aldehyde dehydrogenase (AIDH) activity in intact rat liver mitochondria (0.2 mM) | [6541256](https://www.ncbi.nlm.nih.gov/pubmed/6541256) |
| [CHEMBL641143](https://www.ebi.ac.uk/chembl/assay/inspect/CHEMBL641143) | 5 | Evaluated in vitro for inhibition of mitochondrial aldehyde dehydrogenase (AIDH) activity in disrupted rat liver mitochondria (0.2 mM) | [6541256](https://www.ncbi.nlm.nih.gov/pubmed/6541256) |
| [CHEMBL646632](https://www.ebi.ac.uk/chembl/assay/inspect/CHEMBL646632) | 2 | Compound was tested for enantioselectivity of alcohol dehydrogenase in yeast at a concentration of 100 ug/mL. | [9871718](https://www.ncbi.nlm.nih.gov/pubmed/9871718) |
| [CHEMBL646624](https://www.ebi.ac.uk/chembl/assay/inspect/CHEMBL646624) | 2 | Inhibitory activity against yeast alcohol dehydrogenase | [1433175](https://www.ncbi.nlm.nih.gov/pubmed/1433175) |
| [CHEMBL646623](https://www.ebi.ac.uk/chembl/assay/inspect/CHEMBL646623) | 1 | In vitro inhibitory activity against yeast Alcohol dehydrogenase | [10514271](https://www.ncbi.nlm.nih.gov/pubmed/10514271) |
| [CHEMBL646622](https://www.ebi.ac.uk/chembl/assay/inspect/CHEMBL646622) | 1 | In vitro inhibition against yeast Alcohol dehydrogenase; IA means In active | [7783119](https://www.ncbi.nlm.nih.gov/pubmed/7783119) |
| [CHEMBL646621](https://www.ebi.ac.uk/chembl/assay/inspect/CHEMBL646621) | 7 | In vitro inhibition against yeast Alcohol dehydrogenase | [7783119](https://www.ncbi.nlm.nih.gov/pubmed/7783119) |
| [CHEMBL646620](https://www.ebi.ac.uk/chembl/assay/inspect/CHEMBL646620) | 3 | In vitro inhibition against yeast Alcohol dehydrogenase | [7783118](https://www.ncbi.nlm.nih.gov/pubmed/7783118) |
| [CHEMBL646619](https://www.ebi.ac.uk/chembl/assay/inspect/CHEMBL646619) | 2 | In vitro inhibitory activity against yeast alcohol dehydrogenase; Active | [1433175](https://www.ncbi.nlm.nih.gov/pubmed/1433175) |
| [CHEMBL646616](https://www.ebi.ac.uk/chembl/assay/inspect/CHEMBL646616) | 1 | Percent inhibition of the enzyme Alcohol dehydrogenase was measured in rats which are treated with phenobarbital (PB) | [7392033](https://www.ncbi.nlm.nih.gov/pubmed/7392033) |
| [CHEMBL646615](https://www.ebi.ac.uk/chembl/assay/inspect/CHEMBL646615) | 1 | Percent inhibition of rat hepatic mitochondrial Alcohol dehydrogenase | [10514271](https://www.ncbi.nlm.nih.gov/pubmed/10514271) |
| [CHEMBL646614](https://www.ebi.ac.uk/chembl/assay/inspect/CHEMBL646614) | 5 | Percent inhibition of the enzyme Alcohol dehydrogenase was measured in rats which are untreated with phenobarbital (PB) | [7392033](https://www.ncbi.nlm.nih.gov/pubmed/7392033) |
| [CHEMBL646613](https://www.ebi.ac.uk/chembl/assay/inspect/CHEMBL646613) | 4 | Percent inhibition of the enzyme Alcohol dehydrogenase was measured in rats which are treated with phenobarbital (PB) | [7392033](https://www.ncbi.nlm.nih.gov/pubmed/7392033) |
| [CHEMBL646612](https://www.ebi.ac.uk/chembl/assay/inspect/CHEMBL646612) | 6 | Inhibition of the enzyme Alcohol dehydrogenase was measured in rats which are untreated with phenobarbital (PB) | [7392033](https://www.ncbi.nlm.nih.gov/pubmed/7392033) |
| [CHEMBL646611](https://www.ebi.ac.uk/chembl/assay/inspect/CHEMBL646611) | 4 | Inhibition of the enzyme Alcohol dehydrogenase was measured in rats which are treated with phenobarbital (PB) | [7392033](https://www.ncbi.nlm.nih.gov/pubmed/7392033) |
| [CHEMBL645356](https://www.ebi.ac.uk/chembl/assay/inspect/CHEMBL645356) | 4 | Inhibitory activity against porcine alcohol dehydrogenase; Active | [1433175](https://www.ncbi.nlm.nih.gov/pubmed/1433175) |
| [CHEMBL645232](https://www.ebi.ac.uk/chembl/assay/inspect/CHEMBL645232) | 2 | Compound was tested for enantioselectivity of alcohol dehydrogenase in thermoanaerobium brockii at a concentration of 5 ug/mL. | [9871718](https://www.ncbi.nlm.nih.gov/pubmed/9871718) |
| [CHEMBL645230](https://www.ebi.ac.uk/chembl/assay/inspect/CHEMBL645230) | 2 | Compound was tested for enantioselectivity of alcohol dehydrogenase in lactobacillus kefir at a concentration of 50 ug/mL. | [9871718](https://www.ncbi.nlm.nih.gov/pubmed/9871718) |
| [CHEMBL4009340](https://www.ebi.ac.uk/chembl/assay/inspect/CHEMBL4009340) | 1 | Inhibition of full length recombinant human ALDH1A2 expressed in Escherichia coli BL21(DE3) assessed as reduction in dehydrogenase activity by measuring NAD(P)H level preincubated for 2 mins followed by addition of propionaldehyde as substrate in presence of NAD+ by spectrophotometric method relative to control | [28219011](https://www.ncbi.nlm.nih.gov/pubmed/28219011) |
| [CHEMBL4009339](https://www.ebi.ac.uk/chembl/assay/inspect/CHEMBL4009339) | 12 | Inhibition of full length recombinant human ALDH1A2 expressed in Escherichia coli BL21(DE3) assessed as reduction in dehydrogenase activity by measuring NAD(P)H level preincubated for 2 mins followed by addition of propionaldehyde as substrate in presence of NAD+ by spectrophotometric method | [28219011](https://www.ncbi.nlm.nih.gov/pubmed/28219011) |
| [CHEMBL3419574](https://www.ebi.ac.uk/chembl/assay/inspect/CHEMBL3419574) | 12 | Inhibition of recombinant human ALDH1A2 using propionaldehyde as substrate at 100 uM preincubated for 2 mins with NAD+ followed by substrate addition by UV-Vis spectrophotometric analysis | [25634381](https://www.ncbi.nlm.nih.gov/pubmed/25634381) |
| [CHEMBL3419565](https://www.ebi.ac.uk/chembl/assay/inspect/CHEMBL3419565) | 1 | Effect on recombinant human ALDH1A2 using propionaldehyde as substrate at 20 uM preincubated for 2 mins with NAD+ followed by substrate addition by UV-Vis spectrophotometric analysis | [25634381](https://www.ncbi.nlm.nih.gov/pubmed/25634381) |
| [CHEMBL3418002](https://www.ebi.ac.uk/chembl/assay/inspect/CHEMBL3418002) | 17 | Activation of recombinant human ALDH1A2 using propionaldehyde as substrate at 20 uM preincubated for 2 mins with NAD+ followed by substrate addition by UV-Vis spectrophotometric analysis | [25634381](https://www.ncbi.nlm.nih.gov/pubmed/25634381) |
| [CHEMBL3421188](https://www.ebi.ac.uk/chembl/assay/inspect/CHEMBL3421188) | 3 | Activation of recombinant human ALDH1A2 using propionaldehyde as substrate at 100 uM preincubated for 2 mins with NAD+ followed by substrate addition by UV-Vis spectrophotometric analysis | [25634381](https://www.ncbi.nlm.nih.gov/pubmed/25634381) |
| [CHEMBL3421187](https://www.ebi.ac.uk/chembl/assay/inspect/CHEMBL3421187) | 4 | Inhibition of recombinant human ALDH1A2 using propionaldehyde as substrate preincubated for 2 mins with NAD+ followed by substrate addition by UV-Vis spectrophotometric analysis | [25634381](https://www.ncbi.nlm.nih.gov/pubmed/25634381) |
| [CHEMBL3118138](https://www.ebi.ac.uk/chembl/assay/inspect/CHEMBL3118138) | 1 | Inhibition of human ALDH1A2 using propionaldehyde as substrate up to 250 uM preincubated for 1 min followed by substrate addition by spectrophotometric analysis | [24387105](https://www.ncbi.nlm.nih.gov/pubmed/24387105) |
| [CHEMBL3118129](https://www.ebi.ac.uk/chembl/assay/inspect/CHEMBL3118129) | 24 | Inhibition of human ALDH1A2 using propionaldehyde as substrate up to 100 uM preincubated for 1 min followed by substrate addition by spectrophotometric analysis | [24387105](https://www.ncbi.nlm.nih.gov/pubmed/24387105) |

### Table S11. Summary of Protein Data Bank entries for Retinoic acid receptor alpha (RARa)

| PDB ID | Structure Title | PMID | Year  Dep | Ligands Used |
| --- | --- | --- | --- | --- |
| [1DKF](http://www.rcsb.org/pdb/explore.do?structureId=1DKF) | CRYSTAL STRUCTURE OF A HETERODIMERIC COMPLEX OF RAR AND RXR LIGAND-BINDING DOMAINS | [10882070](https://www.ncbi.nlm.nih.gov/pubmed/10882070) | 1999 | 4-[(4,4-DIMETHYL-1,2,3,4-TETRAHYDRO-[1,2']BINAPTHALENYL-7-CARBONYL)-AMINO]-BENZOIC ACID / OLEIC ACID |
| [1DSZ](http://www.rcsb.org/pdb/explore.do?structureId=1DSZ) | STRUCTURE OF THE RXR/RAR DNA-BINDING DOMAIN HETERODIMER IN COMPLEX WITH THE RETINOIC ACID RESPONSE ELEMENT DR1 | [10698945](https://www.ncbi.nlm.nih.gov/pubmed/10698945) | 2000 | ZINC ION |
| [3A9E](http://www.rcsb.org/pdb/explore.do?structureId=3A9E) | Crystal structure of a mixed agonist-bound RAR-alpha and antagonist-bound RXR-alpha heterodimer ligand binding domains | [21152046](https://www.ncbi.nlm.nih.gov/pubmed/21152046) | 2009 | (2E,4E,6Z)-3-methyl-7-(5,5,8,8-tetramethyl-3-propoxy-5,6,7,8-tetrahydronaphthalen-2-yl)octa-2,4,6-trienoic acid / RETINOIC ACID |
| [3KMR](http://www.rcsb.org/pdb/explore.do?structureId=3KMR) | Crystal structure of RARalpha ligand binding domain in complex with an agonist ligand (Am580) and a coactivator fragment | [20543827](https://www.ncbi.nlm.nih.gov/pubmed/20543827) | 2009 | 4-{[(5,5,8,8-tetramethyl-5,6,7,8-tetrahydronaphthalen-2-yl)carbonyl]amino}benzoic acid |
| [3KMZ](http://www.rcsb.org/pdb/explore.do?structureId=3KMZ) | Crystal structure of RARalpha ligand binding domain in complex with the inverse agonist BMS493 and a corepressor fragment | [20543827](https://www.ncbi.nlm.nih.gov/pubmed/20543827) | 2009 | 4-{(E)-2-[5,5-dimethyl-8-(phenylethynyl)-5,6-dihydronaphthalen-2-yl]ethenyl}benzoic acid / GLYCEROL / S-HYDROXYCYSTEINE |
| [4DQM](http://www.rcsb.org/pdb/explore.do?structureId=4DQM) | Revealing a marine natural product as a novel agonist for retinoic acid receptors with a unique binding mode and antitumor activity | [22642567](https://www.ncbi.nlm.nih.gov/pubmed/22642567) | 2012 | (5S)-4-[(3E,7E)-4,8-dimethyl-10-(2,6,6-trimethylcyclohex-1-en-1-yl)deca-3,7-dien-1-yl]-5-hydroxyfuran-2(5H)-one |
| [5K13](http://www.rcsb.org/pdb/explore.do?structureId=5K13) | Crystal structure of the RAR alpha ligand-binding domain in complex with an antagonist | [27261179](https://www.ncbi.nlm.nih.gov/pubmed/27261179) | 2016 | 4-{5-(3-tert-butylphenyl)-1-[4-(methylsulfonyl)phenyl]-1H-pyrazol-3-yl}benzoic acid |

### Table S12. Summary of ChEMBL results for Retinoic acid receptor alpha (RARa)

| Assay ID | Chem Ct | Assay Description | PMID |
| --- | --- | --- | --- |
| [CHEMBL3880815](https://www.ebi.ac.uk/chembl/assay/inspect/CHEMBL3880815) | 18 | Activation of RARalpha (unknown origin) expressed in human HeLa cells co-expressing ERE-beta-Glob-Luc-SV-Neo incubated for 18 hrs by luciferase reporter gene assay | N/A |
| [CHEMBL3224186](https://www.ebi.ac.uk/chembl/assay/inspect/CHEMBL3224186) | 6 | Inhibition of RAR alpha (unknown origin) | N/A |
| [CHEMBL799803](https://www.ebi.ac.uk/chembl/assay/inspect/CHEMBL799803) | 9 | Binding affinity towards Retinoic acid receptor alpha | N/A |
| [CHEMBL799801](https://www.ebi.ac.uk/chembl/assay/inspect/CHEMBL799801) | 10 | Inhibition of [3H]ATRA binding to retinoic acid receptor RAR alpha | N/A |
| [CHEMBL799715](https://www.ebi.ac.uk/chembl/assay/inspect/CHEMBL799715) | 10 | Percent efficacy for retinoic acid receptor RAR alpha in CV-1 cell transcriptional activation assay | N/A |
| [CHEMBL799710](https://www.ebi.ac.uk/chembl/assay/inspect/CHEMBL799710) | 10 | Effective potency in transcriptional activation assay in CV-1 cells expressing retinoic acid receptor RAR alpha; Not active | N/A |
| [CHEMBL799700](https://www.ebi.ac.uk/chembl/assay/inspect/CHEMBL799700) | 5 | Agonist activity for retinoic acid receptor RAR alpha in transcriptional activation assay; NA means not active (EC50>10e4 nM) | N/A |
| [CHEMBL799699](https://www.ebi.ac.uk/chembl/assay/inspect/CHEMBL799699) | 6 | Agonist activity for retinoic acid receptor RAR alpha in transcriptional activation assay | N/A |
| [CHEMBL799686](https://www.ebi.ac.uk/chembl/assay/inspect/CHEMBL799686) | 25 | Maximum retinoic acid receptor (RAR)-alpha transactivation activity relative to retinoic acid at 10e-6 M | N/A |
| [CHEMBL799685](https://www.ebi.ac.uk/chembl/assay/inspect/CHEMBL799685) | 2 | Transcriptional activation activity against retinoic acid receptor RAR alpha; Not active | N/A |
| [CHEMBL799684](https://www.ebi.ac.uk/chembl/assay/inspect/CHEMBL799684) | 10 | Transcriptional activation activity against retinoic acid receptor RAR alpha; Not active | N/A |
| [CHEMBL799683](https://www.ebi.ac.uk/chembl/assay/inspect/CHEMBL799683) | 13 | Transcriptional activation activity against retinoic acid receptor RAR alpha | N/A |
| [CHEMBL798143](https://www.ebi.ac.uk/chembl/assay/inspect/CHEMBL798143) | 1 | Binding affinity for Retinoic acid receptor alpha | N/A |
| [CHEMBL798141](https://www.ebi.ac.uk/chembl/assay/inspect/CHEMBL798141) | 1 | Transactivation potency for Retinoic acid receptor alpha | N/A |
| [CHEMBL802573](https://www.ebi.ac.uk/chembl/assay/inspect/CHEMBL802573) | 6 | Inhibition of [3H]ATRA binding to RAR alpha receptor | N/A |
| [CHEMBL799721](https://www.ebi.ac.uk/chembl/assay/inspect/CHEMBL799721) | 7 | Compound was tested for binding affinity against retinoic acid receptor using 5 nM of [3H]RA as a radioligand in baculovirus expressed receptor | N/A |
| [CHEMBL799717](https://www.ebi.ac.uk/chembl/assay/inspect/CHEMBL799717) | 1 | Binding affinity for Retinoic acid receptor alpha; Not active | N/A |
| [CHEMBL799876](https://www.ebi.ac.uk/chembl/assay/inspect/CHEMBL799876) | 9 | Binding affinity for Retinoic acid receptor alpha | N/A |
| [CHEMBL799865](https://www.ebi.ac.uk/chembl/assay/inspect/CHEMBL799865) | 6 | Maximal transcriptional activation in CV-1 cells expressing RAR alpha receptor relative to ATRA | N/A |
| [CHEMBL799863](https://www.ebi.ac.uk/chembl/assay/inspect/CHEMBL799863) | 8 | Transactivation potency for Retinoic acid receptor alpha; Not active | N/A |
| [CHEMBL879144](https://www.ebi.ac.uk/chembl/assay/inspect/CHEMBL879144) | 2 | Transactivation potency for Retinoic acid receptor alpha | N/A |
| [CHEMBL799859](https://www.ebi.ac.uk/chembl/assay/inspect/CHEMBL799859) | 7 | Compound was tested for functional activity in CV-1 cells transfected with an expression vector for retinoic acid receptor alpha using transactivation assay | N/A |
| [CHEMBL799858](https://www.ebi.ac.uk/chembl/assay/inspect/CHEMBL799858) | 5 | Transcriptional activation in CV-1 cells expressing retinoic acid receptor RAR alpha; not active | N/A |
| [CHEMBL799857](https://www.ebi.ac.uk/chembl/assay/inspect/CHEMBL799857) | 1 | Transcriptional activation in CV-1 cells expressing retinoic acid receptor RAR alpha; inactive | N/A |
| [CHEMBL802230](https://www.ebi.ac.uk/chembl/assay/inspect/CHEMBL802230) | 4 | Transcriptional activation of retinoic acid receptor RAR alpha; not active | N/A |
| [CHEMBL802229](https://www.ebi.ac.uk/chembl/assay/inspect/CHEMBL802229) | 4 | Transcriptional activation of retinoic acid receptor RAR alpha | N/A |
| [CHEMBL799977](https://www.ebi.ac.uk/chembl/assay/inspect/CHEMBL799977) | 3 | Binding affinity towards human Retinoic acid receptor alpha | N/A |
| [CHEMBL772400](https://www.ebi.ac.uk/chembl/assay/inspect/CHEMBL772400) | 13 | Maximal transcriptional activation of RAR-alpha relative to retinoic acid at 10e-6 M | N/A |
| [CHEMBL772399](https://www.ebi.ac.uk/chembl/assay/inspect/CHEMBL772399) | 8 | Transcriptional activation of retinoic acid RAR-alpha receptor; Not active | N/A |
| [CHEMBL772398](https://www.ebi.ac.uk/chembl/assay/inspect/CHEMBL772398) | 5 | Transcriptional activation of retinoic acid RAR-alpha receptor | N/A |
| [CHEMBL772309](https://www.ebi.ac.uk/chembl/assay/inspect/CHEMBL772309) | 2 | Transcriptional activation for RAR alpha receptor; not active (EC50>10e4 nM) | N/A |
| [CHEMBL772308](https://www.ebi.ac.uk/chembl/assay/inspect/CHEMBL772308) | 9 | Transcriptional activation for RAR alpha receptor | N/A |
| [CHEMBL799712](https://www.ebi.ac.uk/chembl/assay/inspect/CHEMBL799712) | 1 | Transcriptional activation in CV-1 cells expressing RAR-alpha receptor; Not active | [9733501](https://www.ncbi.nlm.nih.gov/pubmed/9733501) |
| [CHEMBL799711](https://www.ebi.ac.uk/chembl/assay/inspect/CHEMBL799711) | 9 | Transcriptional activation in CV-1 cells expressing RAR-alpha receptor | [9733501](https://www.ncbi.nlm.nih.gov/pubmed/9733501) |
| [CHEMBL799790](https://www.ebi.ac.uk/chembl/assay/inspect/CHEMBL799790) | 2 | Percent maximal induction of human RAR-alpha receptor relative to ATRA; Inactive | [9703470](https://www.ncbi.nlm.nih.gov/pubmed/9703470) |
| [CHEMBL799789](https://www.ebi.ac.uk/chembl/assay/inspect/CHEMBL799789) | 1 | Percent maximal induction of human RAR-alpha receptor relative to ATRA; Inactive | [9703470](https://www.ncbi.nlm.nih.gov/pubmed/9703470) |
| [CHEMBL799716](https://www.ebi.ac.uk/chembl/assay/inspect/CHEMBL799716) | 3 | Percent maximal induction of human RAR-alpha receptor relative to ATRA | [9703470](https://www.ncbi.nlm.nih.gov/pubmed/9703470) |
| [CHEMBL801810](https://www.ebi.ac.uk/chembl/assay/inspect/CHEMBL801810) | 1 | Relative IC50 for human RAR-alpha receptor as IC50(compound) divided by IC50(ATRA) | [9703470](https://www.ncbi.nlm.nih.gov/pubmed/9703470) |
| [CHEMBL801809](https://www.ebi.ac.uk/chembl/assay/inspect/CHEMBL801809) | 2 | Relative IC50 for human RAR-alpha receptor as IC50(compound) divided by IC50(ATRA) | [9703470](https://www.ncbi.nlm.nih.gov/pubmed/9703470) |
| [CHEMBL801808](https://www.ebi.ac.uk/chembl/assay/inspect/CHEMBL801808) | 1 | Relative EC30 for human RAR-alpha receptor as EC30(compound) divided by EC30(ATRA); Not detected at 500 nM | [9703470](https://www.ncbi.nlm.nih.gov/pubmed/9703470) |
| [CHEMBL801807](https://www.ebi.ac.uk/chembl/assay/inspect/CHEMBL801807) | 1 | Relative EC30 for human RAR-alpha receptor as EC30(compound) divided by EC30(ATRA); Inactive | [9703470](https://www.ncbi.nlm.nih.gov/pubmed/9703470) |
| [CHEMBL801806](https://www.ebi.ac.uk/chembl/assay/inspect/CHEMBL801806) | 1 | Relative EC30 for human RAR-alpha receptor as EC30(compound) divided by EC30(ATRA); Inactive | [9703470](https://www.ncbi.nlm.nih.gov/pubmed/9703470) |
| [CHEMBL799987](https://www.ebi.ac.uk/chembl/assay/inspect/CHEMBL799987) | 5 | Relative EC30 for human RAR-alpha receptor as EC30(compound) divided by EC30(ATRA) | [9703470](https://www.ncbi.nlm.nih.gov/pubmed/9703470) |
| [CHEMBL802719](https://www.ebi.ac.uk/chembl/assay/inspect/CHEMBL802719) | 8 | Inhibition of binding to retinoid A receptor RAR alpha | [9572893](https://www.ncbi.nlm.nih.gov/pubmed/9572893) |
| [CHEMBL801816](https://www.ebi.ac.uk/chembl/assay/inspect/CHEMBL801816) | 1 | Transcriptional activation in CV-1 cells expressing retinoid A receptor RAR alpha | [9572893](https://www.ncbi.nlm.nih.gov/pubmed/9572893) |
| [CHEMBL801815](https://www.ebi.ac.uk/chembl/assay/inspect/CHEMBL801815) | 7 | Transcriptional activation in CV-1 cells expressing retinoid A receptor RAR alpha | [9572893](https://www.ncbi.nlm.nih.gov/pubmed/9572893) |
| [CHEMBL799805](https://www.ebi.ac.uk/chembl/assay/inspect/CHEMBL799805) | 4 | Synergistic activity towards retinoic acid receptor-alpha | [9435893](https://www.ncbi.nlm.nih.gov/pubmed/9435893) |
| [CHEMBL799804](https://www.ebi.ac.uk/chembl/assay/inspect/CHEMBL799804) | 1 | Selective activity towards retinoic acid receptor-alpha | [9435893](https://www.ncbi.nlm.nih.gov/pubmed/9435893) |
| [CHEMBL799798](https://www.ebi.ac.uk/chembl/assay/inspect/CHEMBL799798) | 2 | Antagonistic activity towards retinoic acid receptor-alpha | [9435893](https://www.ncbi.nlm.nih.gov/pubmed/9435893) |
| [CHEMBL873431](https://www.ebi.ac.uk/chembl/assay/inspect/CHEMBL873431) | 2 | Agonistic activity towards retinoic acid receptor-alpha | [9435893](https://www.ncbi.nlm.nih.gov/pubmed/9435893) |
| [CHEMBL799795](https://www.ebi.ac.uk/chembl/assay/inspect/CHEMBL799795) | 6 | Inhibition of [3H]ATRA binding to baculovirus expressed RAR alpha receptor | [9258350](https://www.ncbi.nlm.nih.gov/pubmed/9258350) |
| [CHEMBL857604](https://www.ebi.ac.uk/chembl/assay/inspect/CHEMBL857604) | 4 | Inhibition of [3H]ATRA binding to baculovirus expressed Retinoic acid receptor RAR alpha | [8978832](https://www.ncbi.nlm.nih.gov/pubmed/8978832) |
| [CHEMBL799861](https://www.ebi.ac.uk/chembl/assay/inspect/CHEMBL799861) | 1 | Transcriptional activation in CV-1 cells expressing Retinoic acid receptor RAR alpha | [8978832](https://www.ncbi.nlm.nih.gov/pubmed/8978832) |
| [CHEMBL802722](https://www.ebi.ac.uk/chembl/assay/inspect/CHEMBL802722) | 10 | Percent inhibition of [3H]ATRA binding to mouse Retinoic acid receptor RAR alpha | [8809153](https://www.ncbi.nlm.nih.gov/pubmed/8809153) |
| [CHEMBL801817](https://www.ebi.ac.uk/chembl/assay/inspect/CHEMBL801817) | 10 | Inhibition of [3H]ATRA binding to mouse Retinoic acid receptor RAR alpha | [8809153](https://www.ncbi.nlm.nih.gov/pubmed/8809153) |
| [CHEMBL801814](https://www.ebi.ac.uk/chembl/assay/inspect/CHEMBL801814) | 6 | Transcriptional activation of Retinoic acid receptor RAR alpha;NA=not active | [8784454](https://www.ncbi.nlm.nih.gov/pubmed/8784454) |
| [CHEMBL801813](https://www.ebi.ac.uk/chembl/assay/inspect/CHEMBL801813) | 2 | Transcriptional activation of Retinoic acid receptor RAR alpha | [8784454](https://www.ncbi.nlm.nih.gov/pubmed/8784454) |
| [CHEMBL802721](https://www.ebi.ac.uk/chembl/assay/inspect/CHEMBL802721) | 1 | Dissociation constant for binding to Retinoic acid receptor alpha;NA=not active | [8784454](https://www.ncbi.nlm.nih.gov/pubmed/8784454) |
| [CHEMBL802720](https://www.ebi.ac.uk/chembl/assay/inspect/CHEMBL802720) | 7 | Dissociation constant for binding to Retinoic acid receptor alpha | [8784454](https://www.ncbi.nlm.nih.gov/pubmed/8784454) |
| [CHEMBL799799](https://www.ebi.ac.uk/chembl/assay/inspect/CHEMBL799799) | 3 | Inhibition of [3H]ATRA binding to Retinoic acid receptor RAR alpha | [8765503](https://www.ncbi.nlm.nih.gov/pubmed/8765503) |
| [CHEMBL799702](https://www.ebi.ac.uk/chembl/assay/inspect/CHEMBL799702) | 3 | Transcriptional activation of Retinoic acid receptor RAR alpha | [8765503](https://www.ncbi.nlm.nih.gov/pubmed/8765503) |
| [CHEMBL799875](https://www.ebi.ac.uk/chembl/assay/inspect/CHEMBL799875) | 5 | Binding affinity for baculovirus-expressed Retinoic acid receptor RAR alpha | [8759622](https://www.ncbi.nlm.nih.gov/pubmed/8759622) |
| [CHEMBL877734](https://www.ebi.ac.uk/chembl/assay/inspect/CHEMBL877734) | 4 | Inhibition of [3H]ATRA binding to Retinoic acid receptor RAR alpha | [8709094](https://www.ncbi.nlm.nih.gov/pubmed/8709094) |
| [CHEMBL799866](https://www.ebi.ac.uk/chembl/assay/inspect/CHEMBL799866) | 4 | Percent transcriptional activation relative to ATRA in CV-1 cells expressing Retinoic acid receptor RAR alpha | [8709094](https://www.ncbi.nlm.nih.gov/pubmed/8709094) |
| [CHEMBL884092](https://www.ebi.ac.uk/chembl/assay/inspect/CHEMBL884092) | 4 | Transcriptional activation in CV-1 cells expressing Retinoic acid receptor RAR alpha | [8709094](https://www.ncbi.nlm.nih.gov/pubmed/8709094) |
| [CHEMBL799794](https://www.ebi.ac.uk/chembl/assay/inspect/CHEMBL799794) | 11 | Apparent binding constant against Retinoic acid receptor alpha in HeLa cell GAl-4 transactivation assay | [8691435](https://www.ncbi.nlm.nih.gov/pubmed/8691435) |
| [CHEMBL799802](https://www.ebi.ac.uk/chembl/assay/inspect/CHEMBL799802) | 25 | Binding affinity to retinoic acid receptor alpha using [3H]CD 367 as radioligand | [8544175](https://www.ncbi.nlm.nih.gov/pubmed/8544175) |
| [CHEMBL884081](https://www.ebi.ac.uk/chembl/assay/inspect/CHEMBL884081) | 11 | Effective concentration against RAR-alpha receptor | [8410972](https://www.ncbi.nlm.nih.gov/pubmed/8410972) |
| [CHEMBL772397](https://www.ebi.ac.uk/chembl/assay/inspect/CHEMBL772397) | 17 | Retinoid activity at 10 e-5 M (E)-RA against RAR-alpha receptor for gene transcriptional activation in transfected CV-1 cells | [8410972](https://www.ncbi.nlm.nih.gov/pubmed/8410972) |
| [CHEMBL772396](https://www.ebi.ac.uk/chembl/assay/inspect/CHEMBL772396) | 1 | Effective concentration against RAR-alpha receptor | [8410972](https://www.ncbi.nlm.nih.gov/pubmed/8410972) |
| [CHEMBL799871](https://www.ebi.ac.uk/chembl/assay/inspect/CHEMBL799871) | 6 | Binding affinity against retinoic Acid alpha receptor using [3H]- -9-cis-Retinoic Acid in competitive binding assay | [8308867](https://www.ncbi.nlm.nih.gov/pubmed/8308867) |
| [CHEMBL798386](https://www.ebi.ac.uk/chembl/assay/inspect/CHEMBL798386) | 6 | Binding affinity against retinoic Acid alpha receptors co-transfected into CV-1 cells | [8308867](https://www.ncbi.nlm.nih.gov/pubmed/8308867) |
| [CHEMBL799797](https://www.ebi.ac.uk/chembl/assay/inspect/CHEMBL799797) | 19 | Inhibition of [3H]-ATRA binding to baculovirus expressed retinoid receptor RAR alpha | [8071941](https://www.ncbi.nlm.nih.gov/pubmed/8071941) |
| [CHEMBL799705](https://www.ebi.ac.uk/chembl/assay/inspect/CHEMBL799705) | 20 | Effective concentration against retinoid receptor isoform (RAR alpha) expressed in CV-1 cells | [8071941](https://www.ncbi.nlm.nih.gov/pubmed/8071941) |
| [CHEMBL799618](https://www.ebi.ac.uk/chembl/assay/inspect/CHEMBL799618) | 1 | Inhibition of [3H]ATRA binding to baculovirus expressed retinoid receptor RAR alpha | [8071941](https://www.ncbi.nlm.nih.gov/pubmed/8071941) |
| [CHEMBL1017445](https://www.ebi.ac.uk/chembl/assay/inspect/CHEMBL1017445) | 2 | Transactivation of RARalpha expressed in mammalian cells assessed as effect on palindromic thyroid hormone response element-driven transcriptional activation by luciferase reporter gene assay | [7807131](https://www.ncbi.nlm.nih.gov/pubmed/7807131) |
| [CHEMBL799862](https://www.ebi.ac.uk/chembl/assay/inspect/CHEMBL799862) | 1 | Relative activity against Retinoic acid receptor RAR alpha compared to ATRA | [7650690](https://www.ncbi.nlm.nih.gov/pubmed/7650690) |
| [CHEMBL802725](https://www.ebi.ac.uk/chembl/assay/inspect/CHEMBL802725) | 24 | Relative activity against Retinoic acid receptor alpha at 10e-6 M with respect to 10e-6 M of trans-RA | [7650690](https://www.ncbi.nlm.nih.gov/pubmed/7650690) |
| [CHEMBL802724](https://www.ebi.ac.uk/chembl/assay/inspect/CHEMBL802724) | 1 | Inhibition of [3H]CD 367 binding to Retinoic acid receptor RAR alpha | [7650690](https://www.ncbi.nlm.nih.gov/pubmed/7650690) |
| [CHEMBL799701](https://www.ebi.ac.uk/chembl/assay/inspect/CHEMBL799701) | 10 | Transcriptional activation of Retinoic acid receptor RAR alpha | [7636843](https://www.ncbi.nlm.nih.gov/pubmed/7636843) |
| [CHEMBL802718](https://www.ebi.ac.uk/chembl/assay/inspect/CHEMBL802718) | 7 | Inhibition of murine Retinoic acid receptor RAR alpha | [7608895](https://www.ncbi.nlm.nih.gov/pubmed/7608895) |
| [CHEMBL799720](https://www.ebi.ac.uk/chembl/assay/inspect/CHEMBL799720) | 1 | Binding constant for baculovirus-expressed Retinoic acid receptor RAR alpha | [7490725](https://www.ncbi.nlm.nih.gov/pubmed/7490725) |
| [CHEMBL798387](https://www.ebi.ac.uk/chembl/assay/inspect/CHEMBL798387) | 1 | Transcriptional activation in CV-1 cells expressing Retinoic acid receptor RAR alpha | [7490725](https://www.ncbi.nlm.nih.gov/pubmed/7490725) |
| [CHEMBL4037125](https://www.ebi.ac.uk/chembl/assay/inspect/CHEMBL4037125) | 3 | Agonist activity at RARaplha (unknown origin) | [28850227](https://www.ncbi.nlm.nih.gov/pubmed/28850227) |
| [CHEMBL4019429](https://www.ebi.ac.uk/chembl/assay/inspect/CHEMBL4019429) | 1 | Transactivation of human GAL4-fused RARalpha ligand binding domain expressed in HEL293T cells at 10 uM after 12 to 14 hrs by dual-glo luciferase reporter gene assay | [28845983](https://www.ncbi.nlm.nih.gov/pubmed/28845983) |
| [CHEMBL4040808](https://www.ebi.ac.uk/chembl/assay/inspect/CHEMBL4040808) | 1 | Antagonist activity at GAL4-tagged human RARalpha1 ligand binding domain (177 to 462 residues) expressed in HEK293T cells at 1 uM after 12 to 14 hrs by dual-glo luciferase reporter gene assay | [28749691](https://www.ncbi.nlm.nih.gov/pubmed/28749691) |
| [CHEMBL4040793](https://www.ebi.ac.uk/chembl/assay/inspect/CHEMBL4040793) | 1 | Transactivation of GAL4-tagged human RARalpha1 ligand binding domain (177 to 462 residues) expressed in HEK293T cells at 1 uM after 12 to 14 hrs by dual-glo luciferase reporter gene assay | [28749691](https://www.ncbi.nlm.nih.gov/pubmed/28749691) |
| [CHEMBL3817435](https://www.ebi.ac.uk/chembl/assay/inspect/CHEMBL3817435) | 1 | Antagonist activity at RARalpha-LBD (unknown origin) expressed in HEK-293 cells assessed as inhibition of ATRA induced Gal4/luciferase gene expression after 24 hrs | [27261179](https://www.ncbi.nlm.nih.gov/pubmed/27261179) |
| [CHEMBL3817429](https://www.ebi.ac.uk/chembl/assay/inspect/CHEMBL3817429) | 11 | Displacement of [3H]-TTNPB from RARalpha/RXRalpha (unknown origin) expressed in baculovirus expression system by scintillation proximity assay | [27261179](https://www.ncbi.nlm.nih.gov/pubmed/27261179) |
| [CHEMBL3789268](https://www.ebi.ac.uk/chembl/assay/inspect/CHEMBL3789268) | 3 | Activation of human RARalpha at 1 uM by luciferase reporter gene assay | [26918322](https://www.ncbi.nlm.nih.gov/pubmed/26918322) |
| [CHEMBL3778102](https://www.ebi.ac.uk/chembl/assay/inspect/CHEMBL3778102) | 1 | Inhibition of human RARalpha | [26819660](https://www.ncbi.nlm.nih.gov/pubmed/26819660) |
| [CHEMBL3599161](https://www.ebi.ac.uk/chembl/assay/inspect/CHEMBL3599161) | 1 | Agonist activity at Gal4 DNA binding domain-tagged human RARalpha ligand binding domain expressed in HEK293 cells assessed as activation of receptor-mediated transcriptional activity by luciferase reporter gene assay | [26048806](https://www.ncbi.nlm.nih.gov/pubmed/26048806) |
| [CHEMBL3599039](https://www.ebi.ac.uk/chembl/assay/inspect/CHEMBL3599039) | 1 | Antagonist activity at Gal4 DNA binding domain-tagged human RARalpha ligand binding domain expressed in HEK293 cells assessed as inhibition of receptor-mediated transcriptional activity by luciferase reporter gene assay | [26048806](https://www.ncbi.nlm.nih.gov/pubmed/26048806) |
| [CHEMBL3583847](https://www.ebi.ac.uk/chembl/assay/inspect/CHEMBL3583847) | 1 | Binding affinity to RARalpha (unknown origin) at 10 uM | [26023814](https://www.ncbi.nlm.nih.gov/pubmed/26023814) |
| [CHEMBL3405117](https://www.ebi.ac.uk/chembl/assay/inspect/CHEMBL3405117) | 1 | Agonist activity at RARalpha (unknown origin) expressed in HEK293 cells assessed as transcriptional activation at 0.01 to 0.1 uM after 48 hrs by luciferase reporter gene assay | [25838141](https://www.ncbi.nlm.nih.gov/pubmed/25838141) |
| [CHEMBL3405115](https://www.ebi.ac.uk/chembl/assay/inspect/CHEMBL3405115) | 4 | Agonist activity at RARalpha (unknown origin) expressed in HEK293 cells assessed as transcriptional activation after 48 hrs by luciferase reporter gene assay in presence of bexarotene | [25838141](https://www.ncbi.nlm.nih.gov/pubmed/25838141) |
| [CHEMBL3405114](https://www.ebi.ac.uk/chembl/assay/inspect/CHEMBL3405114) | 8 | Agonist activity at RARalpha (unknown origin) expressed in HEK293 cells assessed as transcriptional activation after 48 hrs by luciferase reporter gene assay | [25838141](https://www.ncbi.nlm.nih.gov/pubmed/25838141) |
| [CHEMBL3405113](https://www.ebi.ac.uk/chembl/assay/inspect/CHEMBL3405113) | 5 | Agonist activity at RARalpha (unknown origin) expressed in HEK293 cells assessed as transcriptional activation at 1 uM after 48 hrs by luciferase reporter gene assay | [25838141](https://www.ncbi.nlm.nih.gov/pubmed/25838141) |
| [CHEMBL3385866](https://www.ebi.ac.uk/chembl/assay/inspect/CHEMBL3385866) | 2 | Agonist activity at human RARalpha expressed in HEK293 cells by luciferase reporter gene assay | [25305688](https://www.ncbi.nlm.nih.gov/pubmed/25305688) |
| [CHEMBL3370745](https://www.ebi.ac.uk/chembl/assay/inspect/CHEMBL3370745) | 1 | Antagonist activity against RARalpha ligand binding domain (unknown origin) expressed in human MCF7 cells assessed as inhibition of ATRA-induced receptor transactivation at 10 uM after 18 hrs by luciferase reporter gene based mammalian one-hybrid assay | [25057340](https://www.ncbi.nlm.nih.gov/pubmed/25057340) |
| [CHEMBL3370749](https://www.ebi.ac.uk/chembl/assay/inspect/CHEMBL3370749) | 1 | Antagonist activity against RXRalpha/RARalpha ligand binding domain (unknown origin) expressed in human HEK293T cells assessed as inhibition of 9-cis-RA/T09-induced receptor transactivation at 2 and 10 uM after 18 hrs by luciferase reporter gene based mammalian one-hybrid assay | [25057340](https://www.ncbi.nlm.nih.gov/pubmed/25057340) |
| [CHEMBL3368375](https://www.ebi.ac.uk/chembl/assay/inspect/CHEMBL3368375) | 2 | Activation of human RAR-alpha expressed in HEK293 cells coexpressing with tk-betaRE-Luc after 24 hrs by luciferase reporter gene assay | [24959987](https://www.ncbi.nlm.nih.gov/pubmed/24959987) |
| [CHEMBL3295537](https://www.ebi.ac.uk/chembl/assay/inspect/CHEMBL3295537) | 3 | Antagonist activity at Gal4-fused mouse RAR-alpha transfected in human HeLa cells after 12 hrs by luciferase reporter gene assay | [24900875](https://www.ncbi.nlm.nih.gov/pubmed/24900875) |
| [CHEMBL3295530](https://www.ebi.ac.uk/chembl/assay/inspect/CHEMBL3295530) | 3 | Agonist activity at Gal4-fused mouse RAR-alpha transfected in human HeLa cells at 1 uM after 12 hrs by luciferase reporter gene assay | [24900875](https://www.ncbi.nlm.nih.gov/pubmed/24900875) |
| [CHEMBL3295525](https://www.ebi.ac.uk/chembl/assay/inspect/CHEMBL3295525) | 3 | Agonist activity at Gal4-fused mouse RAR-alpha transfected in human HeLa cells after 12 hrs by luciferase reporter gene assay | [24900875](https://www.ncbi.nlm.nih.gov/pubmed/24900875) |
| [CHEMBL3367188](https://www.ebi.ac.uk/chembl/assay/inspect/CHEMBL3367188) | 1 | Activity at RARalpha (unknown origin) | [24831826](https://www.ncbi.nlm.nih.gov/pubmed/24831826) |
| [CHEMBL3297476](https://www.ebi.ac.uk/chembl/assay/inspect/CHEMBL3297476) | 5 | Agonist activity at Gal4-fused human RAR-alpha expressed in HEK293 cells assessed as receptor-mediated transcriptional activity at 1 uM treated 24 hrs after transfection measured 48 hrs post-transfection by dual luciferase reporter assay | [24801499](https://www.ncbi.nlm.nih.gov/pubmed/24801499) |
| [CHEMBL3292973](https://www.ebi.ac.uk/chembl/assay/inspect/CHEMBL3292973) | 5 | Agonist activity at human RARalpha expressed in HEK293 cells assessed as transcriptional activation at 0.1 to 50 uM after 40 hrs by luciferase reporter gene assay | [24799257](https://www.ncbi.nlm.nih.gov/pubmed/24799257) |
| [CHEMBL3292971](https://www.ebi.ac.uk/chembl/assay/inspect/CHEMBL3292971) | 1 | Agonist activity at human RARalpha expressed in HEK293 cells assessed as transcriptional activation at 1 to 100 uM after 40 hrs by luciferase reporter gene assay in presence of RAR antagonist LE135 | [24799257](https://www.ncbi.nlm.nih.gov/pubmed/24799257) |
| [CHEMBL3292970](https://www.ebi.ac.uk/chembl/assay/inspect/CHEMBL3292970) | 1 | Agonist activity at human RARalpha expressed in HEK293 cells assessed as transcriptional activation at 1 to 100 uM after 40 hrs by luciferase reporter gene assay | [24799257](https://www.ncbi.nlm.nih.gov/pubmed/24799257) |
| [CHEMBL3118436](https://www.ebi.ac.uk/chembl/assay/inspect/CHEMBL3118436) | 25 | Agonist activity at Gal4-fused RARalpha (unknown origin) transfected in HEK293 cells assessed as induction of transcriptional activity at 4 uM after 6 hrs by luciferase/beta-galactosidase reporter gene assay relative to control | [24457093](https://www.ncbi.nlm.nih.gov/pubmed/24457093) |
| [CHEMBL3118434](https://www.ebi.ac.uk/chembl/assay/inspect/CHEMBL3118434) | 24 | Antagonist activity at Gal4-fused RARalpha (unknown origin) transfected in HEK293 cells assessed as ATRA-induced transcriptional activity at 4 uM by luciferase/beta-galactosidase reporter gene assay relative to ATRA-treated control | [24457093](https://www.ncbi.nlm.nih.gov/pubmed/24457093) |
| [CHEMBL3118432](https://www.ebi.ac.uk/chembl/assay/inspect/CHEMBL3118432) | 3 | Antagonist activity at Gal4-fused RARalpha (unknown origin) transfected in HEK293 cells assessed as inhibition of ATRA-induced transcriptional activity by luciferase/beta-galactosidase reporter gene assay | [24457093](https://www.ncbi.nlm.nih.gov/pubmed/24457093) |
| [CHEMBL3118431](https://www.ebi.ac.uk/chembl/assay/inspect/CHEMBL3118431) | 1 | Inverse agonist activity at RARalpha (unknown origin) | [24457093](https://www.ncbi.nlm.nih.gov/pubmed/24457093) |
| [CHEMBL3100731](https://www.ebi.ac.uk/chembl/assay/inspect/CHEMBL3100731) | 3 | Agonist activity at human RARalpha expressed in HEK293 cells assessed as transcriptional activation at 10'-6 M after 48 hrs by luciferase reporter gene assay | [24359708](https://www.ncbi.nlm.nih.gov/pubmed/24359708) |
| [CHEMBL2389682](https://www.ebi.ac.uk/chembl/assay/inspect/CHEMBL2389682) | 2 | Antagonist activity at human RARalpha expressed in human HeLa cells assessed as inhibition of ATRA-induced RARE transactivation after 24 hrs by chloramphenicol acetyltransferase reporter gene assay | [24040487](https://www.ncbi.nlm.nih.gov/pubmed/24040487) |
| [CHEMBL2389681](https://www.ebi.ac.uk/chembl/assay/inspect/CHEMBL2389681) | 2 | Antagonist activity at human RARalpha expressed in human HeLa cells assessed as inhibition of ATRA-induced RARE-chloramphenicol acetyltransferase reporter gene expression after 24 hrs by transactivation competition assay | [24040487](https://www.ncbi.nlm.nih.gov/pubmed/24040487) |
| [CHEMBL2401373](https://www.ebi.ac.uk/chembl/assay/inspect/CHEMBL2401373) | 36 | Transactivation of Gal4-fused RARalpha (unknown origin) expressed in African green monkey COS1 cells at 100 nM by luciferase reporter gene assay relative to DMSO-treated control | [23685180](https://www.ncbi.nlm.nih.gov/pubmed/23685180) |
| [CHEMBL2401367](https://www.ebi.ac.uk/chembl/assay/inspect/CHEMBL2401367) | 1 | Activation of RXRalpha (unknown origin) by CAT reporter gene assay | [23685180](https://www.ncbi.nlm.nih.gov/pubmed/23685180) |
| [CHEMBL2379064](https://www.ebi.ac.uk/chembl/assay/inspect/CHEMBL2379064) | 1 | Antagonist activity at RARalpha in human MCF7 cells assessed as inhibition of ATRA-induced luciferase activity after 48 hrs by luciferase reporter gene assay | [23474897](https://www.ncbi.nlm.nih.gov/pubmed/23474897) |
| [CHEMBL2379063](https://www.ebi.ac.uk/chembl/assay/inspect/CHEMBL2379063) | 1 | Antagonist activity at RARalpha in human MCF7 cells assessed as inhibition of ATRA-induced luciferase activity at 10 nM after 48 hrs by luciferase reporter gene assay | [23474897](https://www.ncbi.nlm.nih.gov/pubmed/23474897) |
| [CHEMBL2379966](https://www.ebi.ac.uk/chembl/assay/inspect/CHEMBL2379966) | 1 | Antagonist activity at RARalpha in human MCF7 cells assessed as inhibition of ATRA-induced luciferase activity at 0.1 to 1 nM after 48 hrs by luciferase reporter gene assay | [23474897](https://www.ncbi.nlm.nih.gov/pubmed/23474897) |
| [CHEMBL2341874](https://www.ebi.ac.uk/chembl/assay/inspect/CHEMBL2341874) | 1 | Transactivation of RARalpha (unknown origin) by luciferase reporter gene assay relative to Am80 | [23391145](https://www.ncbi.nlm.nih.gov/pubmed/23391145) |
| [CHEMBL2051205](https://www.ebi.ac.uk/chembl/assay/inspect/CHEMBL2051205) | 1 | Induction of RARalpha degradation | [22658364](https://www.ncbi.nlm.nih.gov/pubmed/22658364) |
| [CHEMBL1961828](https://www.ebi.ac.uk/chembl/assay/inspect/CHEMBL1961828) | 2 | Effect on RARA(NR1B1) dependent reporter activity in HEK293 cells at 20 uM | [22460951](https://www.ncbi.nlm.nih.gov/pubmed/22460951) |
| [CHEMBL2155741](https://www.ebi.ac.uk/chembl/assay/inspect/CHEMBL2155741) | 1 | Displacement of [3H]ATRA from RARalpha overexpressed in COS cell extract at 10 nM after 12 hrs by liquid scintillation counting | [21684744](https://www.ncbi.nlm.nih.gov/pubmed/21684744) |
| [CHEMBL2155740](https://www.ebi.ac.uk/chembl/assay/inspect/CHEMBL2155740) | 1 | Inhibition of transactivation of RARalpha overexpressed in COS cells after 24 hrs by luciferase reporter gene assay in presence of ATRA | [21684744](https://www.ncbi.nlm.nih.gov/pubmed/21684744) |
| [CHEMBL2155736](https://www.ebi.ac.uk/chembl/assay/inspect/CHEMBL2155736) | 1 | Displacement of [3H]ATRA from RARalpha overexpressed in COS cell extract after 12 hrs by liquid scintillation counting | [21684744](https://www.ncbi.nlm.nih.gov/pubmed/21684744) |
| [CHEMBL1815811](https://www.ebi.ac.uk/chembl/assay/inspect/CHEMBL1815811) | 2 | Transcriptional activation of human RARalpha at 1 uM by (TREpal)2-tk-CAT reporter gene assay | [21548569](https://www.ncbi.nlm.nih.gov/pubmed/21548569) |
| [CHEMBL1815808](https://www.ebi.ac.uk/chembl/assay/inspect/CHEMBL1815808) | 2 | Transcriptional activation of human RARalpha at 0.1 uM by luciferase reporter gene assay relative to control | [21548569](https://www.ncbi.nlm.nih.gov/pubmed/21548569) |
| [CHEMBL1768345](https://www.ebi.ac.uk/chembl/assay/inspect/CHEMBL1768345) | 2 | Antagonist activity at mouse RARalpha expressed in COS-1 cells assessed as inhibition of Am80-induced transactivation by luciferase reporter gene assay | [21459577](https://www.ncbi.nlm.nih.gov/pubmed/21459577) |
| [CHEMBL1679782](https://www.ebi.ac.uk/chembl/assay/inspect/CHEMBL1679782) | 1 | Antagonist activity at RARalpha by TR-FRET assay | [21218783](https://www.ncbi.nlm.nih.gov/pubmed/21218783) |
| [CHEMBL1679776](https://www.ebi.ac.uk/chembl/assay/inspect/CHEMBL1679776) | 2 | Agonist activity at RARalpha by TR-FRET assay | [21218783](https://www.ncbi.nlm.nih.gov/pubmed/21218783) |
| [CHEMBL1671298](https://www.ebi.ac.uk/chembl/assay/inspect/CHEMBL1671298) | 6 | Effect on human Gal4-fused RAR alpha at 10 uM by luciferase reporter gene assay | [21142112](https://www.ncbi.nlm.nih.gov/pubmed/21142112) |
| [CHEMBL1274439](https://www.ebi.ac.uk/chembl/assay/inspect/CHEMBL1274439) | 1 | Agonist activity at RARalpha | [20925433](https://www.ncbi.nlm.nih.gov/pubmed/20925433) |
| [CHEMBL1227766](https://www.ebi.ac.uk/chembl/assay/inspect/CHEMBL1227766) | 4 | Transactivation of RARalpha in human COS7 cells at 10 uM by luciferase reporter gene assay | [20656484](https://www.ncbi.nlm.nih.gov/pubmed/20656484) |
| [CHEMBL1211362](https://www.ebi.ac.uk/chembl/assay/inspect/CHEMBL1211362) | 3 | Agonist activity at RARalpha-LBD expressed in HEK293 cells assessed as Gal4-DBD interaction by cellular mammalian one hybrid assay relative to retinoic acid | [20638278](https://www.ncbi.nlm.nih.gov/pubmed/20638278) |
| [CHEMBL1211337](https://www.ebi.ac.uk/chembl/assay/inspect/CHEMBL1211337) | 3 | Agonist activity at RARalpha-LBD expressed in HEK293 cells assessed as Gal4-DBD interaction by cellular mammalian one hybrid assay | [20638278](https://www.ncbi.nlm.nih.gov/pubmed/20638278) |
| [CHEMBL1062038](https://www.ebi.ac.uk/chembl/assay/inspect/CHEMBL1062038) | 4 | Agonist activity at human RARalpha expressed in human Caco-2 cells assessed as induction of RARE-mediated transcriptional activity at 100 nM after 24 hrs by luciferase reporter gene assay relative to all-trans retinoic acid | [19791803](https://www.ncbi.nlm.nih.gov/pubmed/19791803) |
| [CHEMBL1061276](https://www.ebi.ac.uk/chembl/assay/inspect/CHEMBL1061276) | 4 | Agonist activity at human RARalpha expressed in human Caco-2 cells assessed as induction of RARE-mediated transcriptional activity at 1 uM after 24 hrs by luciferase reporter gene assay relative to all-trans retinoic acid | [19791803](https://www.ncbi.nlm.nih.gov/pubmed/19791803) |
| [CHEMBL1063213](https://www.ebi.ac.uk/chembl/assay/inspect/CHEMBL1063213) | 1 | Displacement of radioligand from RARalpha receptor | [19502054](https://www.ncbi.nlm.nih.gov/pubmed/19502054) |
| [CHEMBL1785658](https://www.ebi.ac.uk/chembl/assay/inspect/CHEMBL1785658) | 7 | Antagonist activity at yeast GAL4 fused mouse RARalpha ligand binding domain expressed in HeLa cells assessed as inhibition of TTNPB-induced receptor transactivation by luciferase reporter gene assay | [19482478](https://www.ncbi.nlm.nih.gov/pubmed/19482478) |
| [CHEMBL1785654](https://www.ebi.ac.uk/chembl/assay/inspect/CHEMBL1785654) | 5 | Agonist activity at yeast GAL4 fused mouse RARalpha ligand binding domain expressed in HeLa cells assessed as receptor transactivation by luciferase reporter gene assay | [19482478](https://www.ncbi.nlm.nih.gov/pubmed/19482478) |
| [CHEMBL1021936](https://www.ebi.ac.uk/chembl/assay/inspect/CHEMBL1021936) | 8 | Activity at RARalpha/RXRalpha ligand binding domain assessed as stabilization of RXR helix H12 by fluorescence anisotropy in presence of up to 10 uM coactivator peptide TIF-NR2 | [19408900](https://www.ncbi.nlm.nih.gov/pubmed/19408900) |
| [CHEMBL1021933](https://www.ebi.ac.uk/chembl/assay/inspect/CHEMBL1021933) | 1 | Activity at RARalpha/RXRalpha ligand binding domain assessed as stabilization of RXR helix H12 by fluorescence anisotropy in presence of 1 uM coactivator peptide TIF-NR2 | [19408900](https://www.ncbi.nlm.nih.gov/pubmed/19408900) |
| [CHEMBL1021932](https://www.ebi.ac.uk/chembl/assay/inspect/CHEMBL1021932) | 7 | Activity at RARalpha/RXRalpha ligand binding domain assessed as stabilization of RXR helix H12 by fluorescence anisotropy | [19408900](https://www.ncbi.nlm.nih.gov/pubmed/19408900) |
| [CHEMBL1022116](https://www.ebi.ac.uk/chembl/assay/inspect/CHEMBL1022116) | 10 | Activity at RARalpha expressed in mouse NIH3T3 cells by R-SAT assay | [19239230](https://www.ncbi.nlm.nih.gov/pubmed/19239230) |
| [CHEMBL1022115](https://www.ebi.ac.uk/chembl/assay/inspect/CHEMBL1022115) | 1 | Activity at RARalpha expressed in mouse NIH3T3 cells at <10 uM by R-SAT assay relative to Am-580 | [19239230](https://www.ncbi.nlm.nih.gov/pubmed/19239230) |
| [CHEMBL1022114](https://www.ebi.ac.uk/chembl/assay/inspect/CHEMBL1022114) | 10 | Activity at RARalpha expressed in mouse NIH3T3 cells by R-SAT assay relative to Am-580 | [19239230](https://www.ncbi.nlm.nih.gov/pubmed/19239230) |
| [CHEMBL1031896](https://www.ebi.ac.uk/chembl/assay/inspect/CHEMBL1031896) | 1 | Displacement of [3H]9cRA from RARalpha | [19216008](https://www.ncbi.nlm.nih.gov/pubmed/19216008) |
| [CHEMBL1029369](https://www.ebi.ac.uk/chembl/assay/inspect/CHEMBL1029369) | 7 | Inhibition of transactivation activity of Gal4-LBD fused human RARalpha (156 to 454) transfected in atRA-stimulated african green monkey CV1 cells at 0.2 uM after 6 hrs by Dual-light chemiluminescent assay | [19216008](https://www.ncbi.nlm.nih.gov/pubmed/19216008) |
| [CHEMBL1029368](https://www.ebi.ac.uk/chembl/assay/inspect/CHEMBL1029368) | 4 | Inhibition of transactivation activity of Gal4-LBD fused human RARalpha (156 to 454) transfected in atRA-stimulated african green monkey CV1 cells at 4 uM after 6 hrs by Dual-light chemiluminescent assay | [19216008](https://www.ncbi.nlm.nih.gov/pubmed/19216008) |
| [CHEMBL1029365](https://www.ebi.ac.uk/chembl/assay/inspect/CHEMBL1029365) | 8 | Transactivation of Gal4-LBD fused human RARalpha (156 to 454) transfected in african green monkey CV1 cells assessed as luciferase activity at 2 uM after 6 hrs by Dual-light chemiluminescent assay | [19216008](https://www.ncbi.nlm.nih.gov/pubmed/19216008) |
| [CHEMBL1011985](https://www.ebi.ac.uk/chembl/assay/inspect/CHEMBL1011985) | 5 | Activity at human RARalpha ligand binding domain expressed in COS7 cells co-transfected with Gal4-DBD assessed as transcriptional activation after 16 hrs by Gal4 response element-driven luciferase reporter gene assay relative to all-trans retinoic acid | [19058965](https://www.ncbi.nlm.nih.gov/pubmed/19058965) |
| [CHEMBL1011984](https://www.ebi.ac.uk/chembl/assay/inspect/CHEMBL1011984) | 10 | Activity at human RARalpha ligand binding domain expressed in COS7 cells co-transfected with Gal4-DBD assessed as transcriptional activation after 16 hrs by Gal4 response element-driven luciferase reporter gene assay | [19058965](https://www.ncbi.nlm.nih.gov/pubmed/19058965) |
| [CHEMBL1029015](https://www.ebi.ac.uk/chembl/assay/inspect/CHEMBL1029015) | 5 | Activity at human RARalpha ligand binding domain expressed in COS7 cells co-transfected with Gal4-DBD assessed as transcriptional activation at 10 uM after 16 hrs by Gal4 response element-driven luciferase reporter gene assay relative to all-trans retinoic acid | [19058965](https://www.ncbi.nlm.nih.gov/pubmed/19058965) |
| [CHEMBL1015265](https://www.ebi.ac.uk/chembl/assay/inspect/CHEMBL1015265) | 2 | Increase in transcriptional activity of RARalpha ligand binding domain expressed in human Hep G2 cells co-transfected with Gal4-DBD by luciferase reporter gene assay | [19053776](https://www.ncbi.nlm.nih.gov/pubmed/19053776) |
| [CHEMBL1023990](https://www.ebi.ac.uk/chembl/assay/inspect/CHEMBL1023990) | 8 | Agonist activity at human RARalpha expressed in human HeLa cells assessed as relative luminescence units at >=10 uM by luciferase assay relative to control | [18951029](https://www.ncbi.nlm.nih.gov/pubmed/18951029) |
| [CHEMBL970653](https://www.ebi.ac.uk/chembl/assay/inspect/CHEMBL970653) | 5 | Antagonist activity at human RARalpha expressed in african green monkey CV1 cells assessed as all-trans-retinoic acid-stimulated Gal4 transactivation activity by luciferase reporter gene assay | [18702457](https://www.ncbi.nlm.nih.gov/pubmed/18702457) |
| [CHEMBL970650](https://www.ebi.ac.uk/chembl/assay/inspect/CHEMBL970650) | 11 | Activity at human RARalpha expressed in african green monkey CV1 cells assessed as enhanced Gal4 transactivation activity by luciferase reporter gene assay | [18702457](https://www.ncbi.nlm.nih.gov/pubmed/18702457) |
| [CHEMBL954844](https://www.ebi.ac.uk/chembl/assay/inspect/CHEMBL954844) | 3 | Increase in transcriptional activity of RARalpha receptor epressed in human HaCaT cells co-transfected with DR5-tk-CAT reporter plasmid assessed as beta-galactosidase activity by CAT-ELISA | [18511283](https://www.ncbi.nlm.nih.gov/pubmed/18511283) |
| [CHEMBL868240](https://www.ebi.ac.uk/chembl/assay/inspect/CHEMBL868240) | 3 | Activity at human recombinant RARalpha by transactivation of TK-MH100x4-LUC reporter gene in HEK293 cells at 10 uM | [16617018](https://www.ncbi.nlm.nih.gov/pubmed/16617018) |
| [CHEMBL916388](https://www.ebi.ac.uk/chembl/assay/inspect/CHEMBL916388) | 7 | Displacement of [11,12-3H]ARTA from RARalpha | [15615521](https://www.ncbi.nlm.nih.gov/pubmed/15615521) |
| [CHEMBL834984](https://www.ebi.ac.uk/chembl/assay/inspect/CHEMBL834984) | 1 | Antagonism of retinoic acid receptor alpha in ATRA treated CV-1 cells co-expressing CRBP-I-tk-CAT reporter at 10e-7 M | [15317450](https://www.ncbi.nlm.nih.gov/pubmed/15317450) |
| [CHEMBL834860](https://www.ebi.ac.uk/chembl/assay/inspect/CHEMBL834860) | 1 | Antagonism of retinoic acid receptor alpha in ATRA treated CV-1 cells co-expressing CRBP-I-tk-CAT reporter at 10e-6 M | [15317450](https://www.ncbi.nlm.nih.gov/pubmed/15317450) |
| [CHEMBL834854](https://www.ebi.ac.uk/chembl/assay/inspect/CHEMBL834854) | 1 | Antagonism of retinoic acid receptor alpha in ATRA treated CV-1 cells co-expressing CRBP-I-tk-CAT reporter; control assay without compound | [15317450](https://www.ncbi.nlm.nih.gov/pubmed/15317450) |
| [CHEMBL834845](https://www.ebi.ac.uk/chembl/assay/inspect/CHEMBL834845) | 2 | Antagonism of retinoic acid receptor alpha activity induced by retinoic acid at 10e-7 M | [15317450](https://www.ncbi.nlm.nih.gov/pubmed/15317450) |
| [CHEMBL834844](https://www.ebi.ac.uk/chembl/assay/inspect/CHEMBL834844) | 2 | Antagonism of retinoic acid receptor alpha activity induced by retinoic acid at 10e-6 M | [15317450](https://www.ncbi.nlm.nih.gov/pubmed/15317450) |
| [CHEMBL831146](https://www.ebi.ac.uk/chembl/assay/inspect/CHEMBL831146) | 2 | Antagonism of retinoic acid receptor alpha activity induced by retinoic acid at 10e-5 M | [15317450](https://www.ncbi.nlm.nih.gov/pubmed/15317450) |
| [CHEMBL836045](https://www.ebi.ac.uk/chembl/assay/inspect/CHEMBL836045) | 8 | Agonist activity for Retinoic acid receptor alpha as photons in presence and absence of ligand | [15261282](https://www.ncbi.nlm.nih.gov/pubmed/15261282) |
| [CHEMBL798144](https://www.ebi.ac.uk/chembl/assay/inspect/CHEMBL798144) | 3 | Displacement of [3H]9-cis-RA from Retinoic acid receptor alpha LBD | [15214780](https://www.ncbi.nlm.nih.gov/pubmed/15214780) |
| [CHEMBL659140](https://www.ebi.ac.uk/chembl/assay/inspect/CHEMBL659140) | 2 | Transcriptional activation in CV-1 cells expressing RAR alpha at 1E-6M | [15214780](https://www.ncbi.nlm.nih.gov/pubmed/15214780) |
| [CHEMBL799800](https://www.ebi.ac.uk/chembl/assay/inspect/CHEMBL799800) | 17 | Binding affinity for retinoic acid receptor alpha (RARalpha), using 9-cis-[3H]-retinoic acid | [15056000](https://www.ncbi.nlm.nih.gov/pubmed/15056000) |
| [CHEMBL884539](https://www.ebi.ac.uk/chembl/assay/inspect/CHEMBL884539) | 1 | Inhibitory concentration for lipogenesis induced by retinoic acid receptor alpha in C3H10T1/2 clone 8 fibroblast cells | [15056000](https://www.ncbi.nlm.nih.gov/pubmed/15056000) |
| [CHEMBL799709](https://www.ebi.ac.uk/chembl/assay/inspect/CHEMBL799709) | 3 | Effective concentration for lipogenesis induced by retinoic acid receptor alpha in C3H10T1/2 clone 8 fibroblast cells; Not determined | [15056000](https://www.ncbi.nlm.nih.gov/pubmed/15056000) |
| [CHEMBL799708](https://www.ebi.ac.uk/chembl/assay/inspect/CHEMBL799708) | 13 | Effective concentration for retinoic acid receptor alpha induced lipogenesis in C3H10T1/2 clone 8 fibroblast cells | [15056000](https://www.ncbi.nlm.nih.gov/pubmed/15056000) |
| [CHEMBL799707](https://www.ebi.ac.uk/chembl/assay/inspect/CHEMBL799707) | 5 | Effective concentration for Retinoic acid receptor alpha activity in CV-1 cells; Not determined | [15056000](https://www.ncbi.nlm.nih.gov/pubmed/15056000) |
| [CHEMBL799706](https://www.ebi.ac.uk/chembl/assay/inspect/CHEMBL799706) | 12 | Effective concentration for Retinoic acid receptor alpha activity in CV-1 cells | [15056000](https://www.ncbi.nlm.nih.gov/pubmed/15056000) |
| [CHEMBL800465](https://www.ebi.ac.uk/chembl/assay/inspect/CHEMBL800465) | 1 | Displacement of [3H]ATRA from retinoic acid receptor RAR alpha; NT denotes not tested | [15006411](https://www.ncbi.nlm.nih.gov/pubmed/15006411) |
| [CHEMBL799936](https://www.ebi.ac.uk/chembl/assay/inspect/CHEMBL799936) | 18 | Binding affinity against retinoic acid receptor alpha by [3H]ATRA displacement. | [15006411](https://www.ncbi.nlm.nih.gov/pubmed/15006411) |
| [CHEMBL803374](https://www.ebi.ac.uk/chembl/assay/inspect/CHEMBL803374) | 1 | Displacement of [3H]-ATRA from RAR alpha receptor expressed in CV-1 cells; Not tested | [14592510](https://www.ncbi.nlm.nih.gov/pubmed/14592510) |
| [CHEMBL803373](https://www.ebi.ac.uk/chembl/assay/inspect/CHEMBL803373) | 16 | Binding affinity against RAR alpha receptor using [3H]ATRA as radioligand in CV-1 cells | [14592510](https://www.ncbi.nlm.nih.gov/pubmed/14592510) |
| [CHEMBL656174](https://www.ebi.ac.uk/chembl/assay/inspect/CHEMBL656174) | 17 | Agonist synergy against RAR alpha was tested along with 3 nM TTNPB | [14592510](https://www.ncbi.nlm.nih.gov/pubmed/14592510) |
| [CHEMBL799935](https://www.ebi.ac.uk/chembl/assay/inspect/CHEMBL799935) | 4 | Inhibition of [3H]-retinoic acid binding to murine retinoic acid receptor RAR alpha; ND means not determined | [12723955](https://www.ncbi.nlm.nih.gov/pubmed/12723955) |
| [CHEMBL799934](https://www.ebi.ac.uk/chembl/assay/inspect/CHEMBL799934) | 2 | Inhibition of [3H]-retinoic acid binding to murine retinoic acid receptor RAR alpha; NA means not applicable | [12723955](https://www.ncbi.nlm.nih.gov/pubmed/12723955) |
| [CHEMBL799933](https://www.ebi.ac.uk/chembl/assay/inspect/CHEMBL799933) | 8 | Inhibition of [3H]-retinoic acid binding to murine retinoic acid receptor RAR alpha | [12723955](https://www.ncbi.nlm.nih.gov/pubmed/12723955) |
| [CHEMBL799796](https://www.ebi.ac.uk/chembl/assay/inspect/CHEMBL799796) | 6 | Inhibition of [3H]ATRA binding to human Retinoic acid receptor RAR alpha | [12482435](https://www.ncbi.nlm.nih.gov/pubmed/12482435) |
| [CHEMBL799704](https://www.ebi.ac.uk/chembl/assay/inspect/CHEMBL799704) | 3 | Transcriptional activation in CV-1 cells expressing human Retinoic acid receptor RAR alpha; Not active | [12482435](https://www.ncbi.nlm.nih.gov/pubmed/12482435) |
| [CHEMBL799703](https://www.ebi.ac.uk/chembl/assay/inspect/CHEMBL799703) | 4 | Transcriptional activation in CV-1 cells expressing human Retinoic acid receptor RAR alpha | [12482435](https://www.ncbi.nlm.nih.gov/pubmed/12482435) |
| [CHEMBL806021](https://www.ebi.ac.uk/chembl/assay/inspect/CHEMBL806021) | 1 | Inhibition of [3H]ATRA binding to human Retinoic acid receptor RAR alpha | [12482435](https://www.ncbi.nlm.nih.gov/pubmed/12482435) |
| [CHEMBL644678](https://www.ebi.ac.uk/chembl/assay/inspect/CHEMBL644678) | 6 | Percent transcriptional activation of RAR alpha compared to 1 mM ATRA | [12372520](https://www.ncbi.nlm.nih.gov/pubmed/12372520) |
| [CHEMBL644526](https://www.ebi.ac.uk/chembl/assay/inspect/CHEMBL644526) | 1 | Percent transcriptional activation of RAR alpha compared to 1 mM ATRA | [12372520](https://www.ncbi.nlm.nih.gov/pubmed/12372520) |
| [CHEMBL644525](https://www.ebi.ac.uk/chembl/assay/inspect/CHEMBL644525) | 7 | Effective concentration for retinoic acid receptor RAR alpha transcriptional activation | [12372520](https://www.ncbi.nlm.nih.gov/pubmed/12372520) |
| [CHEMBL644524](https://www.ebi.ac.uk/chembl/assay/inspect/CHEMBL644524) | 7 | Binding affinity fo retinoic acid receptor RAR alpha | [12372520](https://www.ncbi.nlm.nih.gov/pubmed/12372520) |
| [CHEMBL724247](https://www.ebi.ac.uk/chembl/assay/inspect/CHEMBL724247) | 1 | Topical irritation induced by RAR alpha-selective retinoid Am-580 in mice; ND denotes not determined. | [12372520](https://www.ncbi.nlm.nih.gov/pubmed/12372520) |
| [CHEMBL724246](https://www.ebi.ac.uk/chembl/assay/inspect/CHEMBL724246) | 2 | Topical irritation induced by RAR alpha-selective retinoid Am-580 in mice | [12372520](https://www.ncbi.nlm.nih.gov/pubmed/12372520) |
| [CHEMBL772307](https://www.ebi.ac.uk/chembl/assay/inspect/CHEMBL772307) | 4 | Inhibition of [3H]ATRA binding to baculovirus expressed retinoic acid receptor RAR-alpha | [11428923](https://www.ncbi.nlm.nih.gov/pubmed/11428923) |
| [CHEMBL772306](https://www.ebi.ac.uk/chembl/assay/inspect/CHEMBL772306) | 2 | Transcriptional activation in CV-1 cells expressing retinoic acid receptor RAR alpha | [11428923](https://www.ncbi.nlm.nih.gov/pubmed/11428923) |
| [CHEMBL799986](https://www.ebi.ac.uk/chembl/assay/inspect/CHEMBL799986) | 3 | Agonistic activity on Retinoic acid receptor alpha by transcriptional activation in COS-1 cells incubated at 10e-9 M | [11392543](https://www.ncbi.nlm.nih.gov/pubmed/11392543) |
| [CHEMBL878612](https://www.ebi.ac.uk/chembl/assay/inspect/CHEMBL878612) | 3 | Agonistic activity on Retinoic acid receptor alpha by transcriptional activation in COS-1 cells incubated at 10e-8 M | [11392543](https://www.ncbi.nlm.nih.gov/pubmed/11392543) |
| [CHEMBL799985](https://www.ebi.ac.uk/chembl/assay/inspect/CHEMBL799985) | 3 | Agonistic activity on Retinoic acid receptor alpha by transcriptional activation in COS-1 cells incubated at 10e-7 M | [11392543](https://www.ncbi.nlm.nih.gov/pubmed/11392543) |
| [CHEMBL799984](https://www.ebi.ac.uk/chembl/assay/inspect/CHEMBL799984) | 2 | Agonistic activity on Retinoic acid receptor alpha by transcriptional activation in COS-1 cells incubated at 10e-6 M | [11392543](https://www.ncbi.nlm.nih.gov/pubmed/11392543) |
| [CHEMBL799983](https://www.ebi.ac.uk/chembl/assay/inspect/CHEMBL799983) | 2 | Agonistic activity on Retinoic acid receptor alpha by transcriptional activation in COS-1 cells incubated at 10e-10 M | [11392543](https://www.ncbi.nlm.nih.gov/pubmed/11392543) |
| [CHEMBL802578](https://www.ebi.ac.uk/chembl/assay/inspect/CHEMBL802578) | 6 | In vitro binding affinity towards Retinoic acid receptor alpha evaluated relative to that of All-trans retinoic acids(Mean of ATRA IC50 = 0.66 nM) | [11354380](https://www.ncbi.nlm.nih.gov/pubmed/11354380) |
| [CHEMBL802575](https://www.ebi.ac.uk/chembl/assay/inspect/CHEMBL802575) | 2 | Retinoid activity at Retinoic acid receptor alpha was evaluated relative to that of All-trans retinoic acids(Mean of ATRA EC50 = 1.06 nM); Not detectable | [11354380](https://www.ncbi.nlm.nih.gov/pubmed/11354380) |
| [CHEMBL802574](https://www.ebi.ac.uk/chembl/assay/inspect/CHEMBL802574) | 4 | Retinoid activity at Retinoic acid receptor alpha was evaluated relative to that of All-trans retinoic acids(Mean of ATRA EC50 = 1.06 nM) | [11354380](https://www.ncbi.nlm.nih.gov/pubmed/11354380) |
| [CHEMBL799870](https://www.ebi.ac.uk/chembl/assay/inspect/CHEMBL799870) | 1 | Antagonistic activity was evaluated in terms of inhibition of Retinoic acid receptor alpha transactivation by ATRA (50 nM) | [11354380](https://www.ncbi.nlm.nih.gov/pubmed/11354380) |
| [CHEMBL799719](https://www.ebi.ac.uk/chembl/assay/inspect/CHEMBL799719) | 9 | Binding affinity towards retinoic acid receptor alpha was determined using [3H]ATRA (5 nM) as radioligand | [11277515](https://www.ncbi.nlm.nih.gov/pubmed/11277515) |
| [CHEMBL799869](https://www.ebi.ac.uk/chembl/assay/inspect/CHEMBL799869) | 5 | Antagonist activity of TTNPB (10 nM) function at retinoic acid receptor alpha | [11277515](https://www.ncbi.nlm.nih.gov/pubmed/11277515) |
| [CHEMBL799867](https://www.ebi.ac.uk/chembl/assay/inspect/CHEMBL799867) | 5 | Efficacy of TTNPB (10 nM) function at retinoic acid receptor alpha (10 uM) | [11277515](https://www.ncbi.nlm.nih.gov/pubmed/11277515) |
| [CHEMBL798385](https://www.ebi.ac.uk/chembl/assay/inspect/CHEMBL798385) | 3 | Ability to inhibit TTNPB-induced transactivation at retinoic acid receptor alpha | [11277515](https://www.ncbi.nlm.nih.gov/pubmed/11277515) |
| [CHEMBL799982](https://www.ebi.ac.uk/chembl/assay/inspect/CHEMBL799982) | 1 | Inhibition of [3H]ATRA binding to Retinoic acid receptor alpha | [10956201](https://www.ncbi.nlm.nih.gov/pubmed/10956201) |
| [CHEMBL799981](https://www.ebi.ac.uk/chembl/assay/inspect/CHEMBL799981) | 11 | Inhibition of [3H]-ATRA binding to Retinoic acid receptor alpha (RAR-alpha) | [10956201](https://www.ncbi.nlm.nih.gov/pubmed/10956201) |
| [CHEMBL799980](https://www.ebi.ac.uk/chembl/assay/inspect/CHEMBL799980) | 11 | Relative transcriptional activation in COS cells expressing Retinoic acid receptor alpha compared to ATRA | [10956201](https://www.ncbi.nlm.nih.gov/pubmed/10956201) |
| [CHEMBL838382](https://www.ebi.ac.uk/chembl/assay/inspect/CHEMBL838382) | 1 | Transcriptional activation in COS cells expressing RAR-alpha | [10956201](https://www.ncbi.nlm.nih.gov/pubmed/10956201) |
| [CHEMBL802580](https://www.ebi.ac.uk/chembl/assay/inspect/CHEMBL802580) | 9 | Percent activation of Retinoic acid receptor alpha at 1 uM relative to 1 uM trans-retinoic acid | [10890153](https://www.ncbi.nlm.nih.gov/pubmed/10890153) |
| [CHEMBL801812](https://www.ebi.ac.uk/chembl/assay/inspect/CHEMBL801812) | 9 | Transcriptional activation in CV-1 cells expressing Retinoic acid receptor alpha | [10890153](https://www.ncbi.nlm.nih.gov/pubmed/10890153) |
| [CHEMBL802579](https://www.ebi.ac.uk/chembl/assay/inspect/CHEMBL802579) | 7 | Percent transcriptional activation of Retinoic acid receptor RAR alpha at 1 uM compared to 1 uM trans-retinoic acid | [10890152](https://www.ncbi.nlm.nih.gov/pubmed/10890152) |
| [CHEMBL801811](https://www.ebi.ac.uk/chembl/assay/inspect/CHEMBL801811) | 7 | Transcriptional activation in CV-1 cells expressing Retinoic acid receptor RAR alpha | [10890152](https://www.ncbi.nlm.nih.gov/pubmed/10890152) |
| [CHEMBL802577](https://www.ebi.ac.uk/chembl/assay/inspect/CHEMBL802577) | 2 | Binding affinity for Retinoic acid receptor alpha relative to ATRA; Not detectable | [10762039](https://www.ncbi.nlm.nih.gov/pubmed/10762039) |
| [CHEMBL802576](https://www.ebi.ac.uk/chembl/assay/inspect/CHEMBL802576) | 8 | Binding affinity for Retinoic acid receptor alpha relative to ATRA | [10762039](https://www.ncbi.nlm.nih.gov/pubmed/10762039) |
| [CHEMBL799873](https://www.ebi.ac.uk/chembl/assay/inspect/CHEMBL799873) | 1 | Binding affinity for Retinoic acid receptor alpha | [10762039](https://www.ncbi.nlm.nih.gov/pubmed/10762039) |
| [CHEMBL659135](https://www.ebi.ac.uk/chembl/assay/inspect/CHEMBL659135) | 1 | Relative transcriptional activation in CV-1 cells expressing RAR alpha receptor compared to ATRA; Not detectable | [10762039](https://www.ncbi.nlm.nih.gov/pubmed/10762039) |
| [CHEMBL659134](https://www.ebi.ac.uk/chembl/assay/inspect/CHEMBL659134) | 9 | Relative transcriptional activation in CV-1 cells expressing RAR alpha receptor compared to ATRA | [10762039](https://www.ncbi.nlm.nih.gov/pubmed/10762039) |
| [CHEMBL654646](https://www.ebi.ac.uk/chembl/assay/inspect/CHEMBL654646) | 1 | Transcriptional activation in CV-1 cells expressing RAR alpha receptor | [10762039](https://www.ncbi.nlm.nih.gov/pubmed/10762039) |
| [CHEMBL799757](https://www.ebi.ac.uk/chembl/assay/inspect/CHEMBL799757) | 4 | Inhibition of [3H]ATRA-Hl60 binding to Retinoic acid receptor alpha relative to ATRA | [10762038](https://www.ncbi.nlm.nih.gov/pubmed/10762038) |
| [CHEMBL802581](https://www.ebi.ac.uk/chembl/assay/inspect/CHEMBL802581) | 1 | Relative binding affinity for Retinoic acid receptor alpha as IC50/IC50(ATRA) | [10762038](https://www.ncbi.nlm.nih.gov/pubmed/10762038) |
| [CHEMBL799872](https://www.ebi.ac.uk/chembl/assay/inspect/CHEMBL799872) | 1 | Inhibition of [3H]ATRA-Hl60 binding to Retinoic acid receptor alpha | [10762038](https://www.ncbi.nlm.nih.gov/pubmed/10762038) |
| [CHEMBL659144](https://www.ebi.ac.uk/chembl/assay/inspect/CHEMBL659144) | 1 | Relative transcriptional activation in CV-1 cells expressing RAR alpha receptor compared to ATRA; Not detectable | [10762038](https://www.ncbi.nlm.nih.gov/pubmed/10762038) |
| [CHEMBL659143](https://www.ebi.ac.uk/chembl/assay/inspect/CHEMBL659143) | 4 | Relative transcriptional activation in CV-1 cells expressing RAR alpha receptor compared to ATRA | [10762038](https://www.ncbi.nlm.nih.gov/pubmed/10762038) |
| [CHEMBL654645](https://www.ebi.ac.uk/chembl/assay/inspect/CHEMBL654645) | 1 | Transcriptional activation in CV-1 cells expressing RAR alpha receptor | [10762038](https://www.ncbi.nlm.nih.gov/pubmed/10762038) |
| [CHEMBL799793](https://www.ebi.ac.uk/chembl/assay/inspect/CHEMBL799793) | 1 | Binding affinity for Retinoic Acid Receptor alpha (RAR alpha). | [10669568](https://www.ncbi.nlm.nih.gov/pubmed/10669568) |
| [CHEMBL799792](https://www.ebi.ac.uk/chembl/assay/inspect/CHEMBL799792) | 6 | Binding affinity for Retinoic Acid Receptor alpha (RAR alpha) | [10669568](https://www.ncbi.nlm.nih.gov/pubmed/10669568) |
| [CHEMBL799791](https://www.ebi.ac.uk/chembl/assay/inspect/CHEMBL799791) | 3 | Binding affinity for Retinoic Acid Receptor alpha (RAR alpha) | [10669568](https://www.ncbi.nlm.nih.gov/pubmed/10669568) |
| [CHEMBL799714](https://www.ebi.ac.uk/chembl/assay/inspect/CHEMBL799714) | 2 | Induction of HL-60 cell differentiation at Retinoic Acid Receptor alpha (RAR alpha) | [10669568](https://www.ncbi.nlm.nih.gov/pubmed/10669568) |
| [CHEMBL800632](https://www.ebi.ac.uk/chembl/assay/inspect/CHEMBL800632) | 8 | Transcriptional activation of Retinoic Acid Receptor alpha (RAR alpha) | [10669568](https://www.ncbi.nlm.nih.gov/pubmed/10669568) |
| [CHEMBL800631](https://www.ebi.ac.uk/chembl/assay/inspect/CHEMBL800631) | 2 | Transcriptional activation of Retinoic Acid Receptor alpha (RAR alpha) | [10669568](https://www.ncbi.nlm.nih.gov/pubmed/10669568) |
| [CHEMBL799979](https://www.ebi.ac.uk/chembl/assay/inspect/CHEMBL799979) | 2 | Binding affinity for Retinoic Acid Receptor alpha (RAR alpha);ND means not detectable. | [10669568](https://www.ncbi.nlm.nih.gov/pubmed/10669568) |
| [CHEMBL799978](https://www.ebi.ac.uk/chembl/assay/inspect/CHEMBL799978) | 2 | Binding affinity for Retinoic Acid Receptor alpha (RAR alpha);ND means not detectable. | [10669568](https://www.ncbi.nlm.nih.gov/pubmed/10669568) |
| [CHEMBL698011](https://www.ebi.ac.uk/chembl/assay/inspect/CHEMBL698011) | 3 | Transcriptional activation in COS-1 cells expressing Retinoic Acid Receptor alpha (RAR alpha) | [10669568](https://www.ncbi.nlm.nih.gov/pubmed/10669568) |
| [CHEMBL698008](https://www.ebi.ac.uk/chembl/assay/inspect/CHEMBL698008) | 1 | Transcriptional activation in COS-1 cells expressing Retinoic Acid Receptor alpha (RAR alpha) | [10669568](https://www.ncbi.nlm.nih.gov/pubmed/10669568) |
| [CHEMBL688572](https://www.ebi.ac.uk/chembl/assay/inspect/CHEMBL688572) | 12 | Induction of HL-60 cell differentiation at Retinoic Acid Receptor alpha (RAR alpha) | [10669568](https://www.ncbi.nlm.nih.gov/pubmed/10669568) |
| [CHEMBL800770](https://www.ebi.ac.uk/chembl/assay/inspect/CHEMBL800770) | 1 | Transcriptional activation of Retinoic acid receptor RAR alpha | [10585206](https://www.ncbi.nlm.nih.gov/pubmed/10585206) |
| [CHEMBL798139](https://www.ebi.ac.uk/chembl/assay/inspect/CHEMBL798139) | 5 | Transcriptional activation of Retinoic acid receptor RAR alpha; NA is Not Active | [10585206](https://www.ncbi.nlm.nih.gov/pubmed/10585206) |
| [CHEMBL800328](https://www.ebi.ac.uk/chembl/assay/inspect/CHEMBL800328) | 6 | In vitro binding affinity for Retinoic acid receptor RAR alpha | [10585206](https://www.ncbi.nlm.nih.gov/pubmed/10585206) |
| [CHEMBL799860](https://www.ebi.ac.uk/chembl/assay/inspect/CHEMBL799860) | 1 | Transcriptional activation of Retinoic acid receptor RAR alpha | [10585206](https://www.ncbi.nlm.nih.gov/pubmed/10585206) |
| [CHEMBL799713](https://www.ebi.ac.uk/chembl/assay/inspect/CHEMBL799713) | 2 | Transcriptional activation in CV-1 cells expressing human Retinoic acid receptor RAR alpha | [10543887](https://www.ncbi.nlm.nih.gov/pubmed/10543887) |
| [CHEMBL798142](https://www.ebi.ac.uk/chembl/assay/inspect/CHEMBL798142) | 3 | Percent transcriptional activation relative to 9-cis RA in CV-1 cells expressing Retinoic acid receptor RAR alpha | [10479291](https://www.ncbi.nlm.nih.gov/pubmed/10479291) |
| [CHEMBL798140](https://www.ebi.ac.uk/chembl/assay/inspect/CHEMBL798140) | 3 | Transcriptional activation in CV-1 cells expressing human Retinoic acid receptor alpha | [10479291](https://www.ncbi.nlm.nih.gov/pubmed/10479291) |
| [CHEMBL846504](https://www.ebi.ac.uk/chembl/assay/inspect/CHEMBL846504) | 6 | Binding affinity of [3H]- RA to baculovirus expressed human RAR alpha | [10201840](https://www.ncbi.nlm.nih.gov/pubmed/10201840) |
| [CHEMBL846503](https://www.ebi.ac.uk/chembl/assay/inspect/CHEMBL846503) | 5 | Relative inhibition of TTNPB activity vs background activity (100% inhibition) against RAR alpha | [10201840](https://www.ncbi.nlm.nih.gov/pubmed/10201840) |
| [CHEMBL874059](https://www.ebi.ac.uk/chembl/assay/inspect/CHEMBL874059) | 5 | Antagonistic activity against RAR alpha in transcriptional activation assay with 32 nM TTNPB | [10201840](https://www.ncbi.nlm.nih.gov/pubmed/10201840) |
| [CHEMBL846502](https://www.ebi.ac.uk/chembl/assay/inspect/CHEMBL846502) | 1 | Antagonistic activity against RAR alpha in transcriptional activation assay with 32 nM TTNPB; NT=Not tested | [10201840](https://www.ncbi.nlm.nih.gov/pubmed/10201840) |
| [CHEMBL799718](https://www.ebi.ac.uk/chembl/assay/inspect/CHEMBL799718) | 4 | Inhibition of [3H]RA binding to retinoic acid receptor RAR alpha | [10098670](https://www.ncbi.nlm.nih.gov/pubmed/10098670) |
| [CHEMBL799874](https://www.ebi.ac.uk/chembl/assay/inspect/CHEMBL799874) | 16 | Ability to displace 3[H](all-E)-retinoic acid (5 nM) from alpha retinoic acid receptor (alpha RAR) using transactivation assay | [10098666](https://www.ncbi.nlm.nih.gov/pubmed/10098666) |
| [CHEMBL802572](https://www.ebi.ac.uk/chembl/assay/inspect/CHEMBL802572) | 4 | Inhibition of [3H]ATRA binding to baculovirus expressed Retinoic acid receptor RAR alpha | [10052980](https://www.ncbi.nlm.nih.gov/pubmed/10052980) |
| [CHEMBL799868](https://www.ebi.ac.uk/chembl/assay/inspect/CHEMBL799868) | 2 | Percent transcriptional activation relative to ATRA in CV-1 cells expressing Retinoic acid receptor RAR alpha | [10052980](https://www.ncbi.nlm.nih.gov/pubmed/10052980) |
| [CHEMBL799864](https://www.ebi.ac.uk/chembl/assay/inspect/CHEMBL799864) | 2 | Transcriptional activation in CV-1 cells expressing Retinoic acid receptor RAR alpha | [10052980](https://www.ncbi.nlm.nih.gov/pubmed/10052980) |

### Table S13. Summary of the Protein Data Bank records for Retinoic Acid Receptor beta (RARb)

| PDB ID | Structure Title | PMID | Year Dep | Ligands Used |
| --- | --- | --- | --- | --- |
| [1HRA](http://www.rcsb.org/pdb/explore.do?structureId=1HRA) | THE SOLUTION STRUCTURE OF THE HUMAN RETINOIC ACID RECEPTOR-BETA DNA-BINDING DOMAIN | [8383553](https://www.ncbi.nlm.nih.gov/pubmed/8383553) | 1993 | ZINC ION |
| [1HRA](http://www.rcsb.org/pdb/explore.do?structureId=1HRA) | THE SOLUTION STRUCTURE OF THE HUMAN RETINOIC ACID RECEPTOR-BETA DNA-BINDING DOMAIN |  | 1993 | ZINC ION |
| [1XAP](http://www.rcsb.org/pdb/explore.do?structureId=1XAP) | Structure of the ligand binding domain of the Retinoic Acid Receptor beta | [15319780](https://www.ncbi.nlm.nih.gov/pubmed/15319780) | 2004 | 4-[(1E)-2-(5,5,8,8-TETRAMETHYL-5,6,7,8-TETRAHYDRONAPHTHALEN-2-YL)PROP-1-ENYL]BENZOIC ACID |
| [1XDK](http://www.rcsb.org/pdb/explore.do?structureId=1XDK) | Crystal Structure of the RARbeta/RXRalpha Ligand Binding Domain Heterodimer in Complex with 9-cis Retinoic Acid and a Fragment of the TRAP220 Coactivator | [15528208](https://www.ncbi.nlm.nih.gov/pubmed/15528208) | 2004 | (9cis)-retinoic acid |
| [4DM6](http://www.rcsb.org/pdb/explore.do?structureId=4DM6) | Crystal structure of RARb LBD homodimer in complex with TTNPB | [22355136](https://www.ncbi.nlm.nih.gov/pubmed/22355136) | 2012 | 4-[(1E)-2-(5,5,8,8-TETRAMETHYL-5,6,7,8-TETRAHYDRONAPHTHALEN-2-YL)PROP-1-ENYL]BENZOIC ACID |
| [4DM8](http://www.rcsb.org/pdb/explore.do?structureId=4DM8) | Crystal structure of RARb LBD in complex with 9cis retinoic acid | [22355136](https://www.ncbi.nlm.nih.gov/pubmed/22355136) | 2012 | RETINOIC ACID |
| [4J5W](http://www.rcsb.org/pdb/explore.do?structureId=4J5W) | Crystal Structure of the apo-PXR/RXRalpha LBD Heterotetramer Complex | [23602807](https://www.ncbi.nlm.nih.gov/pubmed/23602807) | 2013 | MAGNESIUM ION |
| [4JYG](http://www.rcsb.org/pdb/explore.do?structureId=4JYG) | Crystal structure of RARbeta LBD in complex with agonist BMS411 [4-{[(5,5-dimethyl-8-phenyl-5,6-dihydronaphthalen-2-yl)carbonyl]amino}benzoic acid] | [25933005](https://www.ncbi.nlm.nih.gov/pubmed/25933005) | 2013 | 2-{2-[2-(2-{2-[2-(2-ETHOXY-ETHOXY)-ETHOXY]-ETHOXY}-ETHOXY)-ETHOXY]-ETHOXY}-ETHANOL / 4-{[(5,5-dimethyl-8-phenyl-5,6-dihydronaphthalen-2-yl)carbonyl]amino}benzoic acid / CITRATE ANION |
| [4JYH](http://www.rcsb.org/pdb/explore.do?structureId=4JYH) | Crystal structure of RARbeta LBD in complex with selective agonist BMS948 [4-{[(8-phenylnaphthalen-2-yl)carbonyl]amino}benzoic acid] | [25933005](https://www.ncbi.nlm.nih.gov/pubmed/25933005) | 2013 | 4-{[(8-phenylnaphthalen-2-yl)carbonyl]amino}benzoic acid / CITRATE ANION |
| [4JYI](http://www.rcsb.org/pdb/explore.do?structureId=4JYI) | Crystal structure of RARbeta LBD in complex with selective partial agonist BMS641 [3-chloro-4-[(E)-2-(5,5-dimethyl-8-phenyl-5,6-dihydronaphthalen-2-yl)ethenyl]benzoic acid] | [25933005](https://www.ncbi.nlm.nih.gov/pubmed/25933005) | 2013 | 3-chloro-4-[(E)-2-(5,5-dimethyl-8-phenyl-5,6-dihydronaphthalen-2-yl)ethenyl]benzoic acid / CITRATE ANION |
| [5UAN](http://www.rcsb.org/pdb/explore.do?structureId=5UAN) | Crystal structure of multi-domain RAR-beta-RXR-alpha heterodimer on DNA | [29021580](https://www.ncbi.nlm.nih.gov/pubmed/29021580) | 2016 | (9cis)-retinoic acid / RETINOIC ACID / ZINC ION |
| [6SSQ](http://www.rcsb.org/pdb/explore.do?structureId=6SSQ) | Crystal structure of RARbeta LBD in complex with LG 100754 | [31694317](https://www.ncbi.nlm.nih.gov/pubmed/31694317) | 2019 | (2E,4E,6Z)-3-methyl-7-(5,5,8,8-tetramethyl-3-propoxy-5,6,7,8-tetrahydronaphthalen-2-yl)octa-2,4,6-trienoic acid / CITRATE ANION / GLYCEROL |

### Table S14. Summary of the ChEMBL records for Retinoic Acid Receptor beta (RARb)

| Assay ID | Chem Ct | Assay Description | PMID |
| --- | --- | --- | --- |
| [CHEMBL4401913](https://www.ebi.ac.uk/chembl/assay/inspect/CHEMBL4401913) | 20 | Transactivation of GAL4-fused mouse RARbeta-LBD expressed in COS-7 cells after 24 hrs by bright-Glo reagent based assay | [30792038](https://www.ncbi.nlm.nih.gov/pubmed/30792038) |
| [CHEMBL4187006](https://www.ebi.ac.uk/chembl/assay/inspect/CHEMBL4187006) | 1 | Transactivation of mouse Gal4-fused RARbeta-LBD expressed in COS-7 cells after 1 day by bright-Glo reagent based assay | [29288071](https://www.ncbi.nlm.nih.gov/pubmed/29288071) |
| [CHEMBL4187005](https://www.ebi.ac.uk/chembl/assay/inspect/CHEMBL4187005) | 23 | Transactivation of mouse Gal4-fused RARbeta-LBD expressed in COS-7 cells after 1 day by bright-Glo reagent based assay relative to control ATRA | [29288071](https://www.ncbi.nlm.nih.gov/pubmed/29288071) |
| [CHEMBL3295538](https://www.ebi.ac.uk/chembl/assay/inspect/CHEMBL3295538) | 3 | Antagonist activity at Gal4-fused mouse RAR-beta transfected in human HeLa cells after 12 hrs by luciferase reporter gene assay | [24900875](https://www.ncbi.nlm.nih.gov/pubmed/24900875) |
| [CHEMBL3295531](https://www.ebi.ac.uk/chembl/assay/inspect/CHEMBL3295531) | 3 | Agonist activity at Gal4-fused mouse RAR-beta transfected in human HeLa cells at 1 uM after 12 hrs by luciferase reporter gene assay | [24900875](https://www.ncbi.nlm.nih.gov/pubmed/24900875) |
| [CHEMBL3295528](https://www.ebi.ac.uk/chembl/assay/inspect/CHEMBL3295528) | 1 | Agonist activity at Gal4-fused mouse RAR-beta transfected in human HeLa cells at >1 uM after 12 hrs by luciferase reporter gene assay | [24900875](https://www.ncbi.nlm.nih.gov/pubmed/24900875) |
| [CHEMBL3295526](https://www.ebi.ac.uk/chembl/assay/inspect/CHEMBL3295526) | 3 | Agonist activity at Gal4-fused mouse RAR-beta transfected in human HeLa cells at 10 uM after 12 hrs by luciferase reporter gene assay | [24900875](https://www.ncbi.nlm.nih.gov/pubmed/24900875) |
| [CHEMBL1785132](https://www.ebi.ac.uk/chembl/assay/inspect/CHEMBL1785132) | 7 | Antagonist activity at yeast GAL4 fused mouse RARbeta ligand binding domain expressed in HeLa cells assessed as inhibition of TTNPB-induced receptor transactivation by luciferase reporter gene assay | [19482478](https://www.ncbi.nlm.nih.gov/pubmed/19482478) |
| [CHEMBL1768347](https://www.ebi.ac.uk/chembl/assay/inspect/CHEMBL1768347) | 2 | Antagonist activity at mouse RARbeta expressed in COS-1 cells assessed as inhibition of Am80-induced transactivation by luciferase reporter gene assay | [21459577](https://www.ncbi.nlm.nih.gov/pubmed/21459577) |
| [CHEMBL795961](https://www.ebi.ac.uk/chembl/assay/inspect/CHEMBL795961) | 1 | Dissociation constant for binding to Retinoic acid receptor beta;NA=not active | [8784454](https://www.ncbi.nlm.nih.gov/pubmed/8784454) |
| [CHEMBL795960](https://www.ebi.ac.uk/chembl/assay/inspect/CHEMBL795960) | 7 | Dissociation constant for binding to Retinoic acid receptor beta | [8784454](https://www.ncbi.nlm.nih.gov/pubmed/8784454) |
| [CHEMBL795959](https://www.ebi.ac.uk/chembl/assay/inspect/CHEMBL795959) | 8 | Inhibition of binding to retinoid A receptor RAR beta | [9572893](https://www.ncbi.nlm.nih.gov/pubmed/9572893) |
| [CHEMBL795958](https://www.ebi.ac.uk/chembl/assay/inspect/CHEMBL795958) | 6 | Inhibition of murine Retinoic acid receptor RAR beta | [7608895](https://www.ncbi.nlm.nih.gov/pubmed/7608895) |
| [CHEMBL795957](https://www.ebi.ac.uk/chembl/assay/inspect/CHEMBL795957) | 7 | Transcriptional activation in CV-1 cells expressing retinoid A receptor RAR beta | [9572893](https://www.ncbi.nlm.nih.gov/pubmed/9572893) |
| [CHEMBL795956](https://www.ebi.ac.uk/chembl/assay/inspect/CHEMBL795956) | 1 | Transcriptional activation in CV-1 cells expressing retinoid A receptor RAR beta | [9572893](https://www.ncbi.nlm.nih.gov/pubmed/9572893) |

### Table S15. Summary of Protein Data Bank results for Retinoic Acid Receptor gamma (RARg)

| PDB ID | Structure Title | PMID | Year Dep | Ligands Used |
| --- | --- | --- | --- | --- |
| [1EXA](http://www.rcsb.org/pdb/explore.do?structureId=1EXA) | ENANTIOMER DISCRIMINATION ILLUSTRATED BY CRYSTAL STRUCTURES OF THE HUMAN RETINOIC ACID RECEPTOR HRARGAMMA LIGAND BINDING DOMAIN: THE COMPLEX WITH THE ACTIVE R-ENANTIOMER BMS270394. | [10841540](https://www.ncbi.nlm.nih.gov/pubmed/10841540) | 2000 | DODECYL-ALPHA-D-MALTOSIDE / R-3-FLUORO-4-[2-HYDROXY-2-(5,5,8,8-TETRAMETHYL-5,6,7,8,-TETRAHYDRO-NAPHTALEN-2-YL)-ACETYLAMINO]-BENZOIC ACID |
| [1EXX](http://www.rcsb.org/pdb/explore.do?structureId=1EXX) | ENANTIOMER DISCRIMINATION ILLUSTRATED BY CRYSTAL STRUCTURES OF THE HUMAN RETINOIC ACID RECEPTOR HRARGAMMA LIGAND BINDING DOMAIN: THE COMPLEX WITH THE INACTIVE S-ENANTIOMER BMS270395. | [10841540](https://www.ncbi.nlm.nih.gov/pubmed/10841540) | 2000 | 3-FLUORO-4-[2-HYDROXY-2-(5,5,8,8-TETRAMETHYL-5,6,7,8,-TETRAHYDRO-NAPHTALEN-2-YL)-ACETYLAMINO]-BENZOIC ACID / DODECYL-ALPHA-D-MALTOSIDE |
| [1FCX](http://www.rcsb.org/pdb/explore.do?structureId=1FCX) | ISOTYPE SELECTIVITY OF THE HUMAN RETINOIC ACID NUCLEAR RECEPTOR HRAR: THE COMPLEX WITH THE RARGAMMA-SELECTIVE RETINOID BMS184394 | [10964567](https://www.ncbi.nlm.nih.gov/pubmed/10964567) | 2000 | 6-[HYDROXY-(5,5,8,8-TETRAMETHYL-5,6,7,8-TETRAHYDRO-NAPHTALEN-2-YL)-METHYL]-NAPHTALENE-2-CARBOXYLIC ACID / DODECYL-ALPHA-D-MALTOSIDE |
| [1FCY](http://www.rcsb.org/pdb/explore.do?structureId=1FCY) | ISOTYPE SELECTIVITY OF THE HUMAN RETINOIC ACID NUCLEAR RECEPTOR HRAR: THE COMPLEX WITH THE RARBETA/GAMMA-SELECTIVE RETINOID CD564 | [10964567](https://www.ncbi.nlm.nih.gov/pubmed/10964567) | 2000 | 6-(5,5,8,8-TETRAMETHYL-5,6,7,8-TETRAHYDRO-NAPHTALENE-2-CARBONYL)-NAPHTALENE-2-CARBOXYLIC ACID / DODECYL-ALPHA-D-MALTOSIDE |
| [1FCZ](http://www.rcsb.org/pdb/explore.do?structureId=1FCZ) | ISOTYPE SELECTIVITY OF THE HUMAN RETINOIC ACID NUCLEAR RECEPTOR HRAR: THE COMPLEX WITH THE PANAGONIST RETINOID BMS181156 | [10964567](https://www.ncbi.nlm.nih.gov/pubmed/10964567) | 2000 | 4-[3-OXO-3-(5,5,8,8-TETRAMETHYL-5,6,7,8-TETRAHYDRO-NAPHTHALEN-2-YL)-PROPENYL]-BENZOIC ACID / DODECYL-ALPHA-D-MALTOSIDE |
| [1FD0](http://www.rcsb.org/pdb/explore.do?structureId=1FD0) | ISOTYPE SELECTIVITY OF THE HUMAN RETINOIC ACID NUCLEAR RECEPTOR HRAR: THE COMPLEX WITH THE RARGAMMA-SELECTIVE RETINOID SR11254 | [12220491](https://www.ncbi.nlm.nih.gov/pubmed/12220491) | 2000 | 6-[HYDROXYIMINO-(5,5,8,8-TETRAMETHYL-5,6,7,8-TETRAHYDRO-NAPHTALEN-2-YL)-METHYL]-NAPHTALENE-2-CARBOXYLIC ACID / DODECYL-ALPHA-D-MALTOSIDE |
| [2LBD](http://www.rcsb.org/pdb/explore.do?structureId=2LBD) | LIGAND-BINDING DOMAIN OF THE HUMAN RETINOIC ACID RECEPTOR GAMMA BOUND TO ALL-TRANS RETINOIC ACID | [7501014](https://www.ncbi.nlm.nih.gov/pubmed/7501014) | 1997 | RETINOIC ACID |
| [3LBD](http://www.rcsb.org/pdb/explore.do?structureId=3LBD) | LIGAND-BINDING DOMAIN OF THE HUMAN RETINOIC ACID RECEPTOR GAMMA BOUND TO 9-CIS RETINOIC ACID | [9501913](https://www.ncbi.nlm.nih.gov/pubmed/9501913) | 1998 | (9cis)-retinoic acid |
| [4LBD](http://www.rcsb.org/pdb/explore.do?structureId=4LBD) | LIGAND-BINDING DOMAIN OF THE HUMAN RETINOIC ACID RECEPTOR GAMMA BOUND TO THE SYNTHETIC AGONIST BMS961 | [9501913](https://www.ncbi.nlm.nih.gov/pubmed/9501913) | 1998 | 3-FLUORO-4-[2-HYDROXY-2-(5,5,8,8-TETRAMETHYL-5,6,7,8,-TETRAHYDRO-NAPHTALEN-2-YL)-ACETYLAMINO]-BENZOIC ACID |
| [5M24](http://www.rcsb.org/pdb/explore.do?structureId=5M24) | RARg mutant-S371E | [28125680](https://www.ncbi.nlm.nih.gov/pubmed/28125680) | 2016 | (9cis)-retinoic acid / CHLORIDE ION / DODECYL-ALPHA-D-MALTOSIDE |
| [6FX0](http://www.rcsb.org/pdb/explore.do?structureId=6FX0) | Structure-based design of Trifarotene (CD5789), a potent and selective RAR gamma agonist for the treatment of acne | [29706423](https://www.ncbi.nlm.nih.gov/pubmed/29706423) | 2018 | 6-[3-(1-adamantyl)-4-oxidanyl-phenyl]naphthalene-2-carboxylic acid / TETRAETHYLENE GLYCOL |

### Table S16. Summary of ChEMBL results for Retinoic Acid Receptor gamma (RARg)

| Assay ID | Chem Ct | Assay Description | PMID |
| --- | --- | --- | --- |
| [CHEMBL3295539](https://www.ebi.ac.uk/chembl/assay/inspect/CHEMBL3295539) | 3 | Antagonist activity at Gal4-fused mouse RAR-gamma transfected in human HeLa cells after 12 hrs by luciferase reporter gene assay | [24900875](https://www.ncbi.nlm.nih.gov/pubmed/24900875) |
| [CHEMBL3295532](https://www.ebi.ac.uk/chembl/assay/inspect/CHEMBL3295532) | 3 | Agonist activity at Gal4-fused mouse RAR-gamma transfected in human HeLa cells at 1 uM after 12 hrs by luciferase reporter gene assay | [24900875](https://www.ncbi.nlm.nih.gov/pubmed/24900875) |
| [CHEMBL3295527](https://www.ebi.ac.uk/chembl/assay/inspect/CHEMBL3295527) | 3 | Agonist activity at Gal4-fused mouse RAR-gamma transfected in human HeLa cells at 10 uM after 12 hrs by luciferase reporter gene assay | [24900875](https://www.ncbi.nlm.nih.gov/pubmed/24900875) |
| [CHEMBL1785655](https://www.ebi.ac.uk/chembl/assay/inspect/CHEMBL1785655) | 6 | Agonist activity at yeast GAL4 fused mouse RARgamma ligand binding domain expressed in HeLa cells assessed as receptor transactivation by luciferase reporter gene assay | [19482478](https://www.ncbi.nlm.nih.gov/pubmed/19482478) |
| [CHEMBL1785133](https://www.ebi.ac.uk/chembl/assay/inspect/CHEMBL1785133) | 8 | Antagonist activity at yeast GAL4 fused mouse RARgamma ligand binding domain expressed in HeLa cells assessed as inhibition of TTNPB-induced receptor transactivation by luciferase reporter gene assay | [19482478](https://www.ncbi.nlm.nih.gov/pubmed/19482478) |
| [CHEMBL1768348](https://www.ebi.ac.uk/chembl/assay/inspect/CHEMBL1768348) | 2 | Antagonist activity at mouse RARgamma expressed in COS-1 cells assessed as inhibition of ATRA-induced transactivation by luciferase reporter gene assay | [21459577](https://www.ncbi.nlm.nih.gov/pubmed/21459577) |
| [CHEMBL804940](https://www.ebi.ac.uk/chembl/assay/inspect/CHEMBL804940) | 1 | Dissociation constant for binding to Retinoic acid receptor gamma;NA=not active | [8784454](https://www.ncbi.nlm.nih.gov/pubmed/8784454) |
| [CHEMBL804939](https://www.ebi.ac.uk/chembl/assay/inspect/CHEMBL804939) | 7 | Dissociation constant for binding to Retinoic acid receptor gamma | [8784454](https://www.ncbi.nlm.nih.gov/pubmed/8784454) |
| [CHEMBL804938](https://www.ebi.ac.uk/chembl/assay/inspect/CHEMBL804938) | 8 | Inhibition of binding to retinoid A receptor RAR gamma | [9572893](https://www.ncbi.nlm.nih.gov/pubmed/9572893) |
| [CHEMBL804937](https://www.ebi.ac.uk/chembl/assay/inspect/CHEMBL804937) | 6 | Inhibition of binding to murine Retinoic acid receptor RAR gamma | [7608895](https://www.ncbi.nlm.nih.gov/pubmed/7608895) |
| [CHEMBL804936](https://www.ebi.ac.uk/chembl/assay/inspect/CHEMBL804936) | 1 | Transcriptional activation in CV-1 cells expressing retinoic A receptor RAR gamma | [9572893](https://www.ncbi.nlm.nih.gov/pubmed/9572893) |
| [CHEMBL804935](https://www.ebi.ac.uk/chembl/assay/inspect/CHEMBL804935) | 2 | Transcriptional activation in CV-1 cells expressing retinoid A receptor RAR gamma | [9572893](https://www.ncbi.nlm.nih.gov/pubmed/9572893) |
| [CHEMBL804934](https://www.ebi.ac.uk/chembl/assay/inspect/CHEMBL804934) | 1 | Transcriptional activation in CV-1 cells expressing retinoid A receptor RAR gamma | [9572893](https://www.ncbi.nlm.nih.gov/pubmed/9572893) |

### Table S17. ToxCast and Tox21 results. Filtering of results is described in methods. Assays are organized by specific protein targets, metabolism targets, and testing the retinoid pathway in general. Descriptions of the assays can be found in Supplemental Table S18.

|  |  | **Specific Targets** | | | | | **Metabolism** | | **Pathway** | | |
| --- | --- | --- | --- | --- | --- | --- | --- | --- | --- | --- | --- |
|  |  | **RARa** | **RARb** | **RARg** | **RAR** | **RAR** | **CYP1A1** | **CYP2C8** |  |  |  |
| **DSSToxID** | **Chemical Name** | **ATG_RARa_trans_up** | **ATG_RARb_TRANS_up** | **ATG_RARg_TRANS_up** | **NVS_NR_hRAR_Agonist** | **NVS_NR_hRAR_Antagonist** | **NVS_ADME_hCYP1A1** | **NVS_ADME_hCYP2C8** | **ATG_DR5_CIS_up** | **Tox21_RAR_LUC_agonist** | **Tox21_RAR_LUC_antagonist** |
| [DTXSID7021239](https://comptox.epa.gov/dashboard/dsstoxdb/results?search=DTXSID7021239) | all-trans-Retinoic acid |  |  |  | 0.126 |  | 1.317 |  | 0.006 |  |  |
| [DTXSID8024151](https://comptox.epa.gov/dashboard/dsstoxdb/results?search=DTXSID8024151) | Imazalil | 0.908 |  |  |  |  | 1.413 |  |  |  |  |
| [DTXSID6020561](https://comptox.epa.gov/dashboard/dsstoxdb/results?search=DTXSID6020561) | Endrin |  | 1.606 | 1.698 |  |  |  |  | 0.806 |  |  |
| [DTXSID9020453](https://comptox.epa.gov/dashboard/dsstoxdb/results?search=DTXSID9020453) | Dieldrin | 0.77 |  | 1.68 |  |  |  |  | 0.579 |  |  |
| [DTXSID4038922](https://comptox.epa.gov/dashboard/dsstoxdb/results?search=DTXSID4038922) | Tetrabromobisphenol A bis(2-hydroxyethyl) ether | 1.503 |  | 0.84 |  |  |  |  | 0.22 |  |  |
| [DTXSID3023556](https://comptox.epa.gov/dashboard/dsstoxdb/results?search=DTXSID3023556) | Retinol | 0.076 |  | 0.227 |  |  |  |  | 0.197 |  |  |
| [DTXSID0020319](https://comptox.epa.gov/dashboard/dsstoxdb/results?search=DTXSID0020319) | Chlorothalonil |  |  |  |  |  |  | 0.182 | 0.185 |  |  |
| [DTXSID2020347](https://comptox.epa.gov/dashboard/dsstoxdb/results?search=DTXSID2020347) | Coumaphos |  |  |  |  |  | 0.274 |  | 1.596 |  |  |
| [DTXSID7032638](https://comptox.epa.gov/dashboard/dsstoxdb/results?search=DTXSID7032638) | Pyraclostrobin |  |  |  |  |  | 1.678 |  | 0.543 |  |  |
| [DTXSID5040758](https://comptox.epa.gov/dashboard/dsstoxdb/results?search=DTXSID5040758) | AM580 |  |  | 0.08 |  |  |  |  | 0.083 | 0.023 |  |
| [DTXSID0048185](https://comptox.epa.gov/dashboard/dsstoxdb/results?search=DTXSID0048185) | Apomorphine hydrochloride hydrate |  |  | 1.802 |  |  |  |  | 1.683 |  |  |
| [DTXSID2022880](https://comptox.epa.gov/dashboard/dsstoxdb/results?search=DTXSID2022880) | Danazol | 0.734 |  |  |  |  |  |  | 0.535 |  |  |
| [DTXSID2032500](https://comptox.epa.gov/dashboard/dsstoxdb/results?search=DTXSID2032500) | Triflumizole | 1.453 |  |  |  |  |  |  | ##### |  |  |
| [DTXSID7047279](https://comptox.epa.gov/dashboard/dsstoxdb/results?search=DTXSID7047279) | CP-532623 | 1.561 |  |  |  |  |  |  | 0.367 |  |  |
| [DTXSID9037539](https://comptox.epa.gov/dashboard/dsstoxdb/results?search=DTXSID9037539) | Endosulfan I | 1.384 |  |  |  |  |  |  | 1.827 |  |  |
| [DTXSID4024270](https://comptox.epa.gov/dashboard/dsstoxdb/results?search=DTXSID4024270) | Prochloraz |  |  |  |  |  | 0.413 | 1.679 |  |  |  |
| [DTXSID9032329](https://comptox.epa.gov/dashboard/dsstoxdb/results?search=DTXSID9032329) | Bensulide |  |  |  |  |  | 0.551 | 0.662 |  |  |  |
| [DTXSID0021385](https://comptox.epa.gov/dashboard/dsstoxdb/results?search=DTXSID0021385) | Folpet |  |  |  |  |  |  | 0.197 |  |  |  |
| [DTXSID0032655](https://comptox.epa.gov/dashboard/dsstoxdb/results?search=DTXSID0032655) | Triticonazole |  |  |  |  |  | 0.793 |  |  |  |  |
| [DTXSID1024259](https://comptox.epa.gov/dashboard/dsstoxdb/results?search=DTXSID1024259) | Phosalone |  |  |  |  |  | 0.489 |  |  |  |  |
| [DTXSID2040363](https://comptox.epa.gov/dashboard/dsstoxdb/results?search=DTXSID2040363) | Diniconazole |  |  |  |  |  | 0.675 |  |  |  |  |
| [DTXSID3020122](https://comptox.epa.gov/dashboard/dsstoxdb/results?search=DTXSID3020122) | Azinphos-methyl |  |  |  |  |  | 0.331 |  |  |  |  |
| [DTXSID4020242](https://comptox.epa.gov/dashboard/dsstoxdb/results?search=DTXSID4020242) | Captafol |  |  |  |  |  |  | 0.091 |  |  |  |
| [DTXSID4032372](https://comptox.epa.gov/dashboard/dsstoxdb/results?search=DTXSID4032372) | Difenoconazole |  |  |  |  |  | 1.459 |  |  |  |  |
| [DTXSID4042121](https://comptox.epa.gov/dashboard/dsstoxdb/results?search=DTXSID4042121) | Mepanipyrim |  |  |  |  |  | 0.567 |  |  |  |  |
| [DTXSID5020653](https://comptox.epa.gov/dashboard/dsstoxdb/results?search=DTXSID5020653) | Gentian Violet |  |  |  |  |  | 1.816 |  |  |  |  |
| [DTXSID5032525](https://comptox.epa.gov/dashboard/dsstoxdb/results?search=DTXSID5032525) | Bifenazate |  |  |  |  |  | 0.484 |  |  |  |  |
| [DTXSID8022325](https://comptox.epa.gov/dashboard/dsstoxdb/results?search=DTXSID8022325) | 2,2-Bis(4-hydroxyphenyl)-1,1,1-trichloroethane |  |  |  |  |  |  | 1.835 |  |  |  |
| [DTXSID9047259](https://comptox.epa.gov/dashboard/dsstoxdb/results?search=DTXSID9047259) | 1,3-Dichloro-6,7,8,9,10,12-hexahydroazepino[2,1-b] quinazoline hydrochloride (1:1) |  |  |  |  |  | 0.791 |  |  |  |  |
| [DTXSID9047598](https://comptox.epa.gov/dashboard/dsstoxdb/results?search=DTXSID9047598) | 4,4'-Sulfonylbis[2-(prop-2-en-1-yl)phenol] |  |  |  |  |  | 1.946 |  |  |  |  |
| [DTXSID6044519](https://comptox.epa.gov/dashboard/dsstoxdb/results?search=DTXSID6044519) | 1,1,3,3,5-Pentamethyl-4,6-dinitro-2,3-dihydro-1H-indene |  |  | 1.606 |  |  |  |  |  |  |  |
| [DTXSID2026602](https://comptox.epa.gov/dashboard/dsstoxdb/results?search=DTXSID2026602) | 2,4-Di-tert-butylphenol |  |  | 1.514 |  |  |  |  |  |  |  |
| [DTXSID7042065](https://comptox.epa.gov/dashboard/dsstoxdb/results?search=DTXSID7042065) | Isodrin |  |  | 1.077 |  |  |  |  |  |  |  |
| [DTXSID8026193](https://comptox.epa.gov/dashboard/dsstoxdb/results?search=DTXSID8026193) | 1,2,3-Trichlorobenzene |  |  | 0.953 |  |  |  |  |  |  |  |
| [DTXSID8020040](https://comptox.epa.gov/dashboard/dsstoxdb/results?search=DTXSID8020040) | Aldrin |  |  | 0.912 |  |  |  |  |  |  |  |
| [DTXSID0023901](https://comptox.epa.gov/dashboard/dsstoxdb/results?search=DTXSID0023901) | Bentazone |  |  |  | 1.899 |  |  |  |  |  |  |
| [DTXSID2026523](https://comptox.epa.gov/dashboard/dsstoxdb/results?search=DTXSID2026523) | Symclosene |  |  |  |  | 0.264 |  |  |  |  |  |
| [DTXSID4021559](https://comptox.epa.gov/dashboard/dsstoxdb/results?search=DTXSID4021559) | 2,6-Di-tert-butyl-4-nitrophenol | 1.887 |  |  |  |  |  |  |  |  |  |
| [DTXSID4024359](https://comptox.epa.gov/dashboard/dsstoxdb/results?search=DTXSID4024359) | 2,4,5-Trichlorophenol |  | 1.058 |  |  |  |  |  |  |  |  |
| [DTXSID7025895](https://comptox.epa.gov/dashboard/dsstoxdb/results?search=DTXSID7025895) | N-Phenyl-1,4-benzenediamine |  | 0.245 |  |  |  |  |  |  |  |  |
| [DTXSID8037706](https://comptox.epa.gov/dashboard/dsstoxdb/results?search=DTXSID8037706) | Potassium perfluorooctanesulfonate |  |  |  | 1.912 |  |  |  |  |  |  |
| [DTXSID8041379](https://comptox.epa.gov/dashboard/dsstoxdb/results?search=DTXSID8041379) | 2,6-Di-tert-butyl-4-methoxyphenol | 1.071 |  |  |  |  |  |  |  |  |  |
| [DTXSID8041909](https://comptox.epa.gov/dashboard/dsstoxdb/results?search=DTXSID8041909) | Dysprosium(III) chloride |  |  |  |  | 0.854 |  |  |  |  |  |
| [DTXSID9022310](https://comptox.epa.gov/dashboard/dsstoxdb/results?search=DTXSID9022310) | Daidzein |  |  |  | 0.937 |  |  |  |  |  |  |
| [DTXSID0023036](https://comptox.epa.gov/dashboard/dsstoxdb/results?search=DTXSID0023036) | Etretinate |  |  |  |  |  |  |  |  | 1.141 |  |
| [DTXSID0033382](https://comptox.epa.gov/dashboard/dsstoxdb/results?search=DTXSID0033382) | Daunomycin hydrochloride |  |  |  |  |  |  |  |  |  | 1.6299 |
| [DTXSID0034223](https://comptox.epa.gov/dashboard/dsstoxdb/results?search=DTXSID0034223) | Tebufenpyrad |  |  |  |  |  |  |  | 0.48 |  |  |
| [DTXSID0045173](https://comptox.epa.gov/dashboard/dsstoxdb/results?search=DTXSID0045173) | Mitoxantrone dihydrochloride |  |  |  |  |  |  |  |  |  | 0.1172 |
| [DTXSID0047379](https://comptox.epa.gov/dashboard/dsstoxdb/results?search=DTXSID0047379) | SSR126768 |  |  |  |  |  |  |  | 0.423 |  |  |
| [DTXSID0047797](https://comptox.epa.gov/dashboard/dsstoxdb/results?search=DTXSID0047797) | Idarubicin hydrochloride |  |  |  |  |  |  |  |  |  | 1.2778 |
| [DTXSID1020560](https://comptox.epa.gov/dashboard/dsstoxdb/results?search=DTXSID1020560) | Endosulfan |  |  |  |  |  |  |  | 0.894 |  |  |
| [DTXSID1032488](https://comptox.epa.gov/dashboard/dsstoxdb/results?search=DTXSID1032488) | Thiazopyr |  |  |  |  |  |  |  | 1.305 |  |  |
| [DTXSID1034501](https://comptox.epa.gov/dashboard/dsstoxdb/results?search=DTXSID1034501) | gamma-Cyhalothrin |  |  |  |  |  |  |  | 0.974 |  |  |
| [DTXSID1040619](https://comptox.epa.gov/dashboard/dsstoxdb/results?search=DTXSID1040619) | Bexarotene |  |  |  |  |  |  |  | 0.014 |  |  |
| [DTXSID1047368](https://comptox.epa.gov/dashboard/dsstoxdb/results?search=DTXSID1047368) | SSR69071 |  |  |  |  |  |  |  | 1.011 |  |  |
| [DTXSID2021311](https://comptox.epa.gov/dashboard/dsstoxdb/results?search=DTXSID2021311) | 2,4,6-Tris(tert-butyl)phenol |  |  |  |  |  |  |  | 0.756 |  |  |
| [DTXSID2032550](https://comptox.epa.gov/dashboard/dsstoxdb/results?search=DTXSID2032550) | Fenpyroximate (Z,E) |  |  |  |  |  |  |  | 0.026 |  |  |
| [DTXSID2034625](https://comptox.epa.gov/dashboard/dsstoxdb/results?search=DTXSID2034625) | Fluoxastrobin |  |  |  |  |  |  |  | 0.577 |  |  |
| [DTXSID2047272](https://comptox.epa.gov/dashboard/dsstoxdb/results?search=DTXSID2047272) | CJ-013610 |  |  |  |  |  |  |  | 1.857 |  |  |
| [DTXSID3020419](https://comptox.epa.gov/dashboard/dsstoxdb/results?search=DTXSID3020419) | Dibutyltin diacetate |  |  |  |  |  |  |  |  |  | 0.2183 |
| [DTXSID3020621](https://comptox.epa.gov/dashboard/dsstoxdb/results?search=DTXSID3020621) | Fenvalerate |  |  |  |  |  |  |  | 0.755 |  |  |
| [DTXSID3024239](https://comptox.epa.gov/dashboard/dsstoxdb/results?search=DTXSID3024239) | Oxadiazon |  |  |  |  |  |  |  | 0.726 |  |  |
| [DTXSID3042390](https://comptox.epa.gov/dashboard/dsstoxdb/results?search=DTXSID3042390) | Sodium (2-pyridylthio)-N-oxide |  |  |  |  |  |  |  |  |  | 0.5377 |
| [DTXSID3042500](https://comptox.epa.gov/dashboard/dsstoxdb/results?search=DTXSID3042500) | Triphenyltin fluoride |  |  |  |  |  |  |  |  |  | 0.0972 |
| [DTXSID4022153](https://comptox.epa.gov/dashboard/dsstoxdb/results?search=DTXSID4022153) | Tetrabutyltin |  |  |  |  |  |  |  | 0.279 |  |  |
| [DTXSID4024276](https://comptox.epa.gov/dashboard/dsstoxdb/results?search=DTXSID4024276) | Propargite |  |  |  |  |  |  |  | 0.903 |  |  |
| [DTXSID4040767](https://comptox.epa.gov/dashboard/dsstoxdb/results?search=DTXSID4040767) | 3,5-Dichlorosalicyl-3,4-dichloroanilide |  |  |  |  |  |  |  | 1.199 |  |  |
| [DTXSID4047383](https://comptox.epa.gov/dashboard/dsstoxdb/results?search=DTXSID4047383) | AVE3247 |  |  |  |  |  |  |  | 1.38 |  |  |
| [DTXSID4048511](https://comptox.epa.gov/dashboard/dsstoxdb/results?search=DTXSID4048511) | PharmaGSID_48511 |  |  |  |  |  |  |  | 0.546 |  |  |
| [DTXSID4048519](https://comptox.epa.gov/dashboard/dsstoxdb/results?search=DTXSID4048519) | PharmaGSID_48519 |  |  |  |  |  |  |  | 1.222 |  |  |
| [DTXSID5021463](https://comptox.epa.gov/dashboard/dsstoxdb/results?search=DTXSID5021463) | Zinc diethyldithiocarbamate |  |  |  |  |  |  |  |  |  | 1.2383 |
| [DTXSID5025998](https://comptox.epa.gov/dashboard/dsstoxdb/results?search=DTXSID5025998) | Retinal |  |  |  |  |  |  |  |  | 0.504 |  |
| [DTXSID5032573](https://comptox.epa.gov/dashboard/dsstoxdb/results?search=DTXSID5032573) | Pyridaben |  |  |  |  |  |  |  | 1.103 |  |  |
| [DTXSID5034981](https://comptox.epa.gov/dashboard/dsstoxdb/results?search=DTXSID5034981) | Tributyltin benzoate |  |  |  |  |  |  |  | 0.023 |  |  |
| [DTXSID5046481](https://comptox.epa.gov/dashboard/dsstoxdb/results?search=DTXSID5046481) | Adapalene |  |  |  |  |  |  |  |  | 0.033 |  |
| [DTXSID5046691](https://comptox.epa.gov/dashboard/dsstoxdb/results?search=DTXSID5046691) | Tazarotene |  |  |  |  |  |  |  |  | 0.438 |  |
| [DTXSID5046776](https://comptox.epa.gov/dashboard/dsstoxdb/results?search=DTXSID5046776) | Ataluren |  |  |  |  |  |  |  |  |  | 0.5982 |
| [DTXSID5046853](https://comptox.epa.gov/dashboard/dsstoxdb/results?search=DTXSID5046853) | Tamibarotene |  |  |  |  |  |  |  |  | 0.158 |  |
| [DTXSID5047322](https://comptox.epa.gov/dashboard/dsstoxdb/results?search=DTXSID5047322) | SB243213A |  |  |  |  |  |  |  | 1.289 |  |  |
| [DTXSID6020690](https://comptox.epa.gov/dashboard/dsstoxdb/results?search=DTXSID6020690) | Hexachlorophene |  |  |  |  |  |  |  | 0.714 |  |  |
| [DTXSID6021248](https://comptox.epa.gov/dashboard/dsstoxdb/results?search=DTXSID6021248) | Rotenone |  |  |  |  |  |  |  | 0.022 |  |  |
| [DTXSID6021408](https://comptox.epa.gov/dashboard/dsstoxdb/results?search=DTXSID6021408) | Triphenyltin acetate |  |  |  |  |  |  |  |  |  | 0.0408 |
| [DTXSID6025513](https://comptox.epa.gov/dashboard/dsstoxdb/results?search=DTXSID6025513) | Malachite green oxalate |  |  |  |  |  |  |  |  |  | 0.4936 |
| [DTXSID7020267](https://comptox.epa.gov/dashboard/dsstoxdb/results?search=DTXSID7020267) | Chlordane |  |  |  |  |  |  |  | 1.784 |  |  |
| [DTXSID7020558](https://comptox.epa.gov/dashboard/dsstoxdb/results?search=DTXSID7020558) | Emetine dihydrochloride |  |  |  |  |  |  |  |  |  | 0.1883 |
| [DTXSID7022883](https://comptox.epa.gov/dashboard/dsstoxdb/results?search=DTXSID7022883) | Daunorubicin |  |  |  |  |  |  |  |  |  | 0.9245 |
| [DTXSID7026314](https://comptox.epa.gov/dashboard/dsstoxdb/results?search=DTXSID7026314) | Zinc pyrithione |  |  |  |  |  |  |  |  |  | 0.0241 |
| [DTXSID7029241](https://comptox.epa.gov/dashboard/dsstoxdb/results?search=DTXSID7029241) | 2,4-Bis(1-methyl-1-phenylethyl)phenol |  |  |  |  |  |  |  | 1.863 |  |  |
| [DTXSID8020381](https://comptox.epa.gov/dashboard/dsstoxdb/results?search=DTXSID8020381) | Deltamethrin |  |  |  |  |  |  |  | 0.545 |  |  |
| [DTXSID8024238](https://comptox.epa.gov/dashboard/dsstoxdb/results?search=DTXSID8024238) | Oryzalin |  |  |  |  |  |  |  | 1.116 |  |  |
| [DTXSID8034401](https://comptox.epa.gov/dashboard/dsstoxdb/results?search=DTXSID8034401) | Buprofezin |  |  |  |  |  |  |  | 1.084 |  |  |
| [DTXSID8035180](https://comptox.epa.gov/dashboard/dsstoxdb/results?search=DTXSID8035180) | Allethrin |  |  |  |  |  |  |  | 0.746 |  |  |
| [DTXSID8047347](https://comptox.epa.gov/dashboard/dsstoxdb/results?search=DTXSID8047347) | SR271425 |  |  |  |  |  |  |  | 0.419 |  |  |
| [DTXSID9021342](https://comptox.epa.gov/dashboard/dsstoxdb/results?search=DTXSID9021342) | Bithionol |  |  |  |  |  |  |  | 1.807 |  |  |
| [DTXSID9032379](https://comptox.epa.gov/dashboard/dsstoxdb/results?search=DTXSID9032379) | Dithiopyr |  |  |  |  |  |  |  | 1.187 |  |  |
| [DTXSID9032533](https://comptox.epa.gov/dashboard/dsstoxdb/results?search=DTXSID9032533) | Chlorfenapyr |  |  |  |  |  |  |  | 0.503 |  |  |
| [DTXSID9034997](https://comptox.epa.gov/dashboard/dsstoxdb/results?search=DTXSID9034997) | Tributyltetradecylphosphonium chloride |  |  |  |  |  |  |  | 1.307 |  |  |
| [DTXSID9040760](https://comptox.epa.gov/dashboard/dsstoxdb/results?search=DTXSID9040760) | {4-[({2-[3-Fluoro-4-(trifluoromethyl)phenyl]-4-methyl-1,3-thiazol-5-yl}methyl)sulfanyl]-2-methylphenoxy}acetic acid |  |  |  |  |  |  |  | 0.896 |  |  |
| [DTXSID9044796](https://comptox.epa.gov/dashboard/dsstoxdb/results?search=DTXSID9044796) | (Acryloyloxy)(tributyl)stannane |  |  |  |  |  |  |  | 0.022 |  |  |
| [DTXSID9046992](https://comptox.epa.gov/dashboard/dsstoxdb/results?search=DTXSID9046992) | Nicardipine hydrochloride |  |  |  |  |  |  |  | 0.283 |  |  |
| [DTXSID9048512](https://comptox.epa.gov/dashboard/dsstoxdb/results?search=DTXSID9048512) | Ro 23-7637 |  |  |  |  |  |  |  | 1.365 |  |  |
| [DTXSID9058654](https://comptox.epa.gov/dashboard/dsstoxdb/results?search=DTXSID9058654) | BisOPP-A |  |  |  |  |  |  |  | 0.736 |  |  |

### Table S18. Descriptions of ToxCast / Tox21 assays

| Name | Active | Total | Description |
| --- | --- | --- | --- |
| ATG_RARa_TRANS_up | 80 | 3807 | Data from the assay component ATG_RARa_TRANS was analyzed into 1 assay endpoint. This assay endpoint, ATG_RARa_TRANS_up, was analyzed in the positive fitting direction relative to DMSO as the negative control and baseline of activity. Using a type of inducible reporter, measures of mRNA for gain-of-signal activity can be used to understand the reporter gene at the transcription factor-level as they relate to the gene RARA. Furthermore, this assay endpoint can be referred to as a primary readout, because this assay has produced multiple assay endpoints where this one serves a reporter gene function. To generalize the intended target to other relatable targets, this assay endpoint is annotated to the "nuclear receptor" intended target family, where the subfamily is "non-steroidal". |
| ATG_RARb_TRANS_up | 26 | 3807 | Data from the assay component ATG_RARb_TRANS was analyzed into 1 assay endpoint. This assay endpoint, ATG_RARb_TRANS_up, was analyzed in the positive fitting direction relative to DMSO as the negative control and baseline of activity. Using a type of inducible reporter, measures of mRNA for gain-of-signal activity can be used to understand the reporter gene at the transcription factor-level as they relate to the gene RARB. Furthermore, this assay endpoint can be referred to as a primary readout, because this assay has produced multiple assay endpoints where this one serves a reporter gene function. To generalize the intended target to other relatable targets, this assay endpoint is annotated to the "nuclear receptor" intended target family, where the subfamily is "non-steroidal". |
| ATG_RARb_TRANS_dn | 152 | 3807 | ATG_RARb_TRANS_dn was not developed or optimized to detect loss of signal. Use data with caution. |
| ATG_RARg_TRANS_up | 63 | 3807 | Data from the assay component ATG_RARg_TRANS was analyzed into 1 assay endpoint. This assay endpoint, ATG_RARg_TRANS_up, was analyzed in the positive fitting direction relative to DMSO as the negative control and baseline of activity. Using a type of inducible reporter, measures of mRNA for gain-of-signal activity can be used to understand the reporter gene at the transcription factor-level as they relate to the gene RARG. Furthermore, this assay endpoint can be referred to as a primary readout, because this assay has produced multiple assay endpoints where this one serves a reporter gene function. To generalize the intended target to other relatable targets, this assay endpoint is annotated to the "nuclear receptor" intended target family, where the subfamily is "non-steroidal". |
| ATG_RARg_TRANS_dn | 82 | 3807 | ATG_RARg_TRANS_dn was not developed or optimized to detect loss of signal. Use data with caution. |
| TOX21_RAR_LUC_Agonist | 305 | 7521 | Data from the assay component TOX21_RAR_LUC_Agonist was analyzed into 1 assay endpoint. This assay endpoint, TOX21_RAR_LUC_Agonist, was analyzed in the positive fitting direction relative to DMSO as the negative control and baseline of activity. Using a type of inducible reporter, gain-of-signal activity can be used to understand changes in the reporter gene as they relate to the gene RARA. Furthermore, this assay endpoint can be referred to as a primary readout, because the performed assay has only produced 1 assay endpoint. To generalize the intended target to other relatable targets, this assay endpoint is annotated to the nuclear receptor intended target family. |
| TOX21_RAR_LUC_Antagonist | 985 | 7871 | Data from the assay component TOX21_RAR_LUC_Antagonist was analyzed into 1 assay endpoint. This assay endpoint, TOX21_RAR_LUC_Antagonist, was analyzed in the positive fitting direction relative to DMSO as the negative control and baseline of activity. Using a type of inducible reporter, loss-of-signal activity can be used to understand changes in the reporter gene as they relate to the gene RARA. Furthermore, this assay endpoint can be referred to as a primary readout, because this assay has produced multiple assay endpoints where this one serves a reporter gene function. To generalize the intended target to other relatable targets, this assay endpoint is annotated to the nuclear receptor intended target family. |
| ATG_DR5_CIS_up | 339 | 3807 | Data from the assay component ATG_DR5_RAR_CIS was analyzed into 1 assay endpoint. This assay endpoint, ATG_DR5_CIS_up, was analyzed in the positive fitting direction relative to DMSO as the negative control and baseline of activity. Using a type of inducible reporter, measures of mRNA for gain-of-signal activity can be used to understand the reporter gene at the transcription factor-level as they relate to the gene RARA and RARB and RARG. Furthermore, this assay endpoint can be referred to as a primary readout, because this assay has produced multiple assay endpoints where this one serves a reporter gene function. To generalize the intended target to other relatable targets, this assay endpoint is annotated to the "nuclear receptor" intended target family, where the subfamily is "non-steroidal". |
| [ATG_DR5_CIS_dn](https://comptox.epa.gov/dashboard/assay_endpoints/ATG_DR5_CIS_dn) | 153 | 3807 | ATG_DR5_CIS_dn was not developed or optimized to detect loss of signal. Use data with caution |
| NVS_ADME_hCYP1A1 | 111 | 1124 | Data from the assay component NVS_ADME_hCYP1A1 was analyzed into 2 assay endpoints. This assay endpoint, NVS_ADME_hCYP1A1, was analyzed in the positive fitting direction relative to Acetonitrile as the negative control and baseline of activity. Using a type of enzyme reporter, loss-of-signal activity can be used to understand changes in the enzymatic activity as they relate to the gene CYP1A1. Furthermore, this assay endpoint can be referred to as a primary readout, because this assay has produced multiple assay endpoints where this one serves a enzymatic activity function. To generalize the intended target to other relatable targets, this assay endpoint is annotated to the "cyp" intended target family, where the subfamily is "xenobiotic metabolism". |
| NVS_ADME_hCYP2C8 | 24 | 315 | Data from the assay component NVS_ADME_hCYP2C8 was analyzed into 2 assay endpoints. This assay endpoint, NVS_ADME_hCYP2C8, was analyzed in the positive fitting direction relative to Acetonitrile as the negative control and baseline of activity. Using a type of enzyme reporter, loss-of-signal activity can be used to understand changes in the enzymatic activity as they relate to the gene CYP2C8. Furthermore, this assay endpoint can be referred to as a primary readout, because this assay has produced multiple assay endpoints where this one serves a enzymatic activity function. To generalize the intended target to other relatable targets, this assay endpoint is annotated to the "cyp" intended target family, where the subfamily is "xenobiotic metabolism". |
